# Deep integrative models for large-scale human genomics

**DOI:** 10.1093/nar/gkad373

**Published:** 2023-05-24

**Authors:** Arnór I Sigurdsson, Ioannis Louloudis, Karina Banasik, David Westergaard, Ole Winther, Ole Lund, Sisse Rye Ostrowski, Christian Erikstrup, Ole Birger Vesterager Pedersen, Mette Nyegaard, DBDS Genomic Consortium, Karina Banasik, Jakob Bay, Jens Kjærgaard Boldsen, Thorsten Brodersen, Søren Brunak, Kristoffer Burgdorf, Mona Ameri Chalmer, Maria Didriksen, Khoa Manh Dinh, Joseph Dowsett, Christian Erikstrup, Bjarke Feenstra, Frank Geller, Daniel Gudbjartsson, Thomas Folkmann Hansen, Lotte Hindhede, Henrik Hjalgrim, Rikke Louise Jacobsen, Gregor Jemec, Katrine Kaspersen, Bertram Dalskov Kjerulff, Lisette Kogelman, Margit Anita Hørup Larsen, Ioannis Louloudis, Agnete Lundgaard, Susan Mikkelsen, Christina Mikkelsen, Kaspar Rene Nielsen, Ioanna Nissen, Mette Nyegaard, Sisse Rye Ostrowski, Ole Birger Pedersen, Alexander Pil Henriksen, Palle Duun Rohde, Klaus Rostgaard, Michael Schwinn, Kari Stefansson, Hreinn Stefónsson, Erik Sørensen, Unnur Thorsteinsdóttir, Lise Wegner Thørner, Mie Topholm Bruun, Henrik Ullum, Thomas Werge, David Westergaard, Søren Brunak, Bjarni J Vilhjálmsson, Simon Rasmussen

**Affiliations:** Novo Nordisk Foundation Center for Protein Research, Faculty of Health and Medical Sciences, University of Copenhagen, 2200 Copenhagen N, Denmark; The Novo Nordisk Foundation Center for Genomic Mechanisms of Disease, Broad Institute of MIT and Harvard, Cambridge, MA 02142, USA; Novo Nordisk Foundation Center for Protein Research, Faculty of Health and Medical Sciences, University of Copenhagen, 2200 Copenhagen N, Denmark; Novo Nordisk Foundation Center for Protein Research, Faculty of Health and Medical Sciences, University of Copenhagen, 2200 Copenhagen N, Denmark; Novo Nordisk Foundation Center for Protein Research, Faculty of Health and Medical Sciences, University of Copenhagen, 2200 Copenhagen N, Denmark; Section for Cognitive Systems, Department of Applied Mathematics and Computer Science, Technical University of Denmark, 2800 Kgs. Lyngby, Denmark; Bioinformatics Centre, Department of Biology, University of Copenhagen, 2200 Copenhagen N, Denmark; Center for Genomic Medicine, Rigshospitalet (Copenhagen University Hospital), Copenhagen 2100, Denmark; Danish National Genome Center, Ørestads Boulevard 5, 2300 Copenhagen S, Denmark; DTU Health Tech, Department of Health Technology, Technical University of Denmark, 2800 Kgs. Lyngby, Denmark; Department of Clinical Immunology, Rigshospitalet, University of Copenhagen, 2200 Copenhagen N, Denmark; Department of Clinical Medicine, Faculty of Health and Medical Sciences, University of Copenhagen, 2200 Copenhagen N, Denmark; Department of Clinical Immunology, Aarhus University Hospital, 8000 Aarhus C, Denmark; Department of Clinical Medicine, Aarhus University, 8000 Aarhus C, Denmark; Department of Clinical Medicine, Faculty of Health and Medical Sciences, University of Copenhagen, 2200 Copenhagen N, Denmark; Department of Clinical Immunology, Zealand University Hospital, 4600 Køge, Denmark; Department of Health Science and Technology, Aalborg University, DK- 9260 Gistrup, Denmark; Novo Nordisk Foundation Center for Protein Research, Faculty of Health and Medical Sciences, University of Copenhagen, 2200 Copenhagen N, Denmark; National Centre for Register-Based Research (NCRR), Aarhus University, 8000 Aarhus C, Denmark; Lundbeck Foundation Initiative for Integrative Psychiatric Research (iPSYCH), 8210 Aarhus V, Denmark; Bioinformatics Research Centre (BiRC), Aarhus University, 8000 Aarhus C, Denmark; Novo Nordisk Foundation Center for Protein Research, Faculty of Health and Medical Sciences, University of Copenhagen, 2200 Copenhagen N, Denmark; The Novo Nordisk Foundation Center for Genomic Mechanisms of Disease, Broad Institute of MIT and Harvard, Cambridge, MA 02142, USA

## Abstract

Polygenic risk scores (PRSs) are expected to play a critical role in precision medicine. Currently, PRS predictors are generally based on linear models using summary statistics, and more recently individual-level data. However, these predictors mainly capture additive relationships and are limited in data modalities they can use. We developed a deep learning framework (EIR) for PRS prediction which includes a model, genome-local-net (GLN), specifically designed for large-scale genomics data. The framework supports multi-task learning, automatic integration of other clinical and biochemical data, and model explainability. When applied to individual-level data from the UK Biobank, the GLN model demonstrated a competitive performance compared to established neural network architectures, particularly for certain traits, showcasing its potential in modeling complex genetic relationships. Furthermore, the GLN model outperformed linear PRS methods for Type 1 Diabetes, likely due to modeling non-additive genetic effects and epistasis. This was supported by our identification of widespread non-additive genetic effects and epistasis in the context of T1D. Finally, we constructed PRS models that integrated genotype, blood, urine, and anthropometric data and found that this improved performance for 93% of the 290 diseases and disorders considered. EIR is available at https://github.com/arnor-sigurdsson/EIR.

## Graphical Abstract

**Graphical Abstract:**
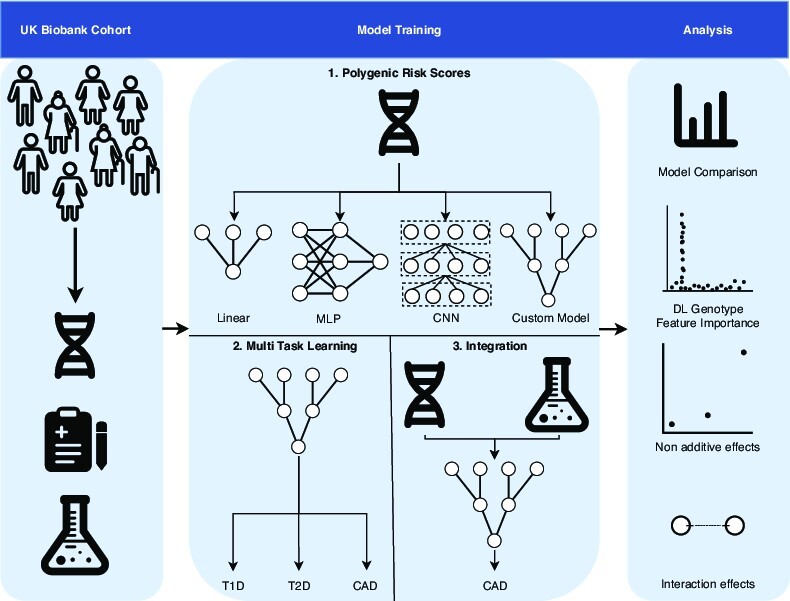

## INTRODUCTION

Polygenic risk scores (PRSs) are becoming increasingly relevant to public health due to larger cohorts and the development of more powerful prediction algorithms. Today, accurate PRS predictors have been trained to predict various human diseases such as type 2 diabetes, coronary artery disease and breast cancer (1–3). Such PRS predictors are expected to become pervasive in clinical human health and decision-making, hence playing a fundamental role in achieving personalized medicine (4–6). PRS predictors can generally be placed in two categories based on the type of training data used, those using summary statistics from genome-wide association studies (GWAS) and those using individual-level data (7). Today, the GWAS-based approach is more prevalent due to larger sample sizes. However, this is rapidly changing with individual-level human genetic variation data increasing in size, with cohorts comprising hundreds of thousands and even millions (8–12). These large individual-level cohorts increasingly offer the opportunity of training accurate predictors for estimating PRSs, which can outperform the combined GWAS-based approach (7). Today, many established methods exist for training predictors on summary statistics (13–17) and individual-level data (18–23), but these predictors generally explore linear relationships.

Deep learning (DL) has gained pace within the recent years and in particular within life sciences (24–30). However, DL frameworks for large discrete data, such as genome-wide data, have not been extensively developed in the field. A potential advantage of DL-based methods for PRS prediction is capturing complex non-linear effects, such as epistasis. Tree-based methods, such as Random Forest, also have the potential of capturing such non-linear effects and have been examined in the context of PRSs (31,32). However, they are limited in the size of data they can accommodate and do not easily extend to other modalities such as text and images. Previous work using neural networks (NNs) for predicting human traits and diseases directly from large-scale genomics has shown worse performance for NN models compared to linear ones (33,34). These results indicate that NNs were unable to capitalize on significant interaction effects, or that no significant interaction effects were present in the data. The latter is in contrast with studies focusing on model organisms, where significant interaction effects have been found (35–37). However, there remains both doubt and controversy regarding the role of complex interaction effects in human traits and diseases (38–42).

However, there are many challenges with building complex NN models that can be applied to human health data. A key challenge is the immense scale of biological data. For example, genomics data often contain millions of genetic variants genotyped for large sample sizes (8,43,44). Traditionally, supervised machine learning tasks are developed to accept one type of input, for instance classifying the main object in a given image. By contrast, health data can be comprised of multi-omics data such as genomics, transcriptomics and proteomics data coupled with targeted biochemical and clinical data, and even include ultra-high resolution imaging. To provide a comprehensive disease risk assessment, methods that can account for genetic, environmental and other risk factors can be advantageous.

Therefore, we developed a DL framework, called EIR, that supports large-scale genomics data and can integrate it with other omics or clinical data. A key feature is a new neural network model, genome-local-net (GLN), that we specifically developed for large-scale genomics data. This model is based on a custom locally-connected layer (LCL) (45–47) that we developed and was, compared to our implementation of least absolute shrinkage and selection operator (LASSO) (48) and other NN models, statistically better overall across 338 diseases, disorders, and traits in the UK Biobank. For eight benchmark traits, we further compared GLN with other established PRS methods and found that GLN outperformed the established methods for T1D. The improvement was particularly noteworthy given the complex genomic interaction effects known to be involved in autoimmune diseases such as T1D (49–51). We found extensive interaction among the most highly important (i.e., relevant for model prediction) T1D SNPs (single-nucleotide polymorphisms), even across chromosomes. Furthermore, all models in EIR extend to multi-task (MT) learning, and we trained one GLN model to predict 338 phenotypes simultaneously. Finally, we used EIR to integrate genotype data, age, and sex covariates, blood measurements, urine measurements and various anthropometrics across 290 diseases in the UK Biobank (UKBB). We found clear improvement with integration for almost all traits, highlighting the potential of deep integrative models for health-based predictions. By applying explainable AI, we identify relevant SNPs and clinical measurements concordant with disease literature.

## MATERIALS AND METHODS

### Processing of UK Biobank genotype and clinical data

The genotype data was processed using Plink (52), version v1.90b6.10. After processing, the genotype data was converted to 459 576 one-hot encoded (i.e. each genotype is encoded separately, with the fourth value representing a missing genotype) sample arrays of shape (4, 803 113) each, in the case of no quality control (NO-QC). The one-hot encoding was chosen to allow the DL models to more easily use non-additive effects such as dominance (rather than them having to learn it from an additive [0–2] encoding). Furthermore, the encoding should in theory allow the LASSO implementation to model on such effects, in contrast to encoding the genotypes with an additive prior. The NO-QC approach might include signals from rare variants that otherwise would be filtered out using a minor allele frequency threshold, and previous work have shown negligible differences between kinship filtered and unfiltered sets of the UKBB data (53). However, we did not filter for linkage disequilibrium (LD) which can dilute the signal across multiple SNPs, instead of being concentrated to one SNP representative of the LD block. When applying quality control (QC), we used the following parameters in Plink, –maf 0.001 –geno 0.03 –mind 0.1 as well as removing samples with a kinship of more than 0.1. After applying QC, there were 425 439 one-hot encoded sample arrays of shape (4, 662 143) each. X chromosomes were included in both cases. Unless otherwise specified, age, sex and the first 10 genotype principal components were included during training. For the tabular data, continuous columns were standardized using the training set statistics in all experiments, meaning that the values computed for the training set were applied to the validation and test sets. Missing biochemical measurement input values were imputed with the averages from the training set. Categorical columns were numerically encoded, missing values were marked as ‘NA’ before numerical encoding. ICD-10 codes were used to derive the disease phenotypes. For comparing DL and linear models, we selected 8 traits based on the authors’ perception of them being a common occurrence in the PRS literature. Recognizing the informal way in how these were chosen, we also analyzed DL, LASSO and covariate-only based models on 338 traits in the UKBB that had a case count over 1000 (hereafter referred to as ‘large-scale’ experiments). Only samples with a self-reported British, Irish, or any other Western European background were used for the main experiments, which amounted to 413 736 samples in the training/validation sets and 45 840 in the test set in the NO-QC case. In the QC case, this resulted in 382 894 and 42 545 samples in the training/validation and test sets respectively. Performance on the held-out tests set are reported as the average and 95% CIs after preforming 1000 bootstrap replicates, following a similar approach as applied before (14). For the integration experiments, we removed samples where the measurements used for integration were measured after a disease diagnosis. This was to avoid feature leakage (i.e. the model having access to features during training and evaluation which do not reflect real scenarios), where e.g. a drug for a certain disease influences measurement values. An alternative could be to mark the measurements as missing and allow them to be subsequently imputed with the train set statistics. However, this might bias the case data towards having all the measurements imputed, which the model might learn. Hence, it is not certain that such approaches would completely prevent feature leakage. Out of the 338 diseases, 48 did not have any time of diagnosis associated with them, and we therefore excluded these from the integration experiments and analysis.

### Training implementation and approach

All models, including the LASSO, were implemented using Pytorch (54), version 1.7.1. A held-out test set was used for all models to get a final performance after training and evaluating on train and validation sets, respectively. We used negative log likelihood loss during training for the classification tasks. All models were trained with a batch size of 64 except for the large MT model (i.e, predicting 338 traits simultaneously) which used a batch size of 32. During training, we used plateau learning rate scheduling to reduce the learning rate by a factor of 0.2 if the validation performance had not improved for 10 steps. The validation interval was calculated dynamically based on the number of cases for a given disease trait (*C*/*B* where *C* is case count and *B* is batch size, with thresholds of 100 and 2000 for the minimum and maximum intervals respectively), as was the number of validation samples used (max[10 000, −1.5 × *C* + 50 000] where *C* is case count). We used early stopping to terminate training when performance had not improved for a certain number of validation steps. We used 16 and 20 steps for traits with less than and more than or equal to 2500 cases, respectively. For the early stopping, we also used a buffer of a certain number of iterations before it was activated, using 1000 iterations for the 8 trait benchmark and the MT experiments and 2000 iterations for the large-scale experiments. Weighted sampling with respect to the target variable was used in all runs during training. All models were trained with the Adam optimizer (55). In the NN based models, we used a weight decay of 1 × 10^−3^ with decoupled weight decay regularization (56). All NN based models used a learning rate of 1 × 10^−4^, while the LASSO models used a learning rate of 5 × 10^−5^. We found that lower learning rate for LASSO gave better training stability and overall results. All neural network architectures used the SiLU (57,58) (also known as Swish (59)) activation function with a trainable parameter β inside the sigmoid function. When using weight decay, we did not apply it to the β parameter. For the neural network models, we augmented the input by randomly setting 40% of the SNPs as missing in the one-hot encoded array, this is similar to input dropout (60) and we found it to be important to prevent overfitting in the NN models. For the LASSO, we used L1 regularization with λ = 1 × 10^−3^ for traits that had more than 2500 cases and λ = 1 × 10^−2^ for traits that had less. All models were trained on a single 16GB NVIDIA® V100 Tensor Core GPU.

### Architectures

This section details how the model architectures were implemented, which are broadly depicted in Supplementary Figures S1 and S2. The LASSO implementation was fit on genotypes separately (i.e. instead of an additive [0–2] encoding as one feature, we use a categorical one-hot encoding for each SNP, meaning each genotype has its separate weight). This should in theory allow the LASSO implementation to utilize non-additive effects such as dominance. Interactions effects were not explicitly included in the model. The MLP feature extractor was one FC layer with 10 output nodes. The main building blocks of the CNN feature extractor were residual blocks, with the first block using full pre-activation (61,62). We added squeeze-and-excitation (SE) blocks (63) to the residual blocks, which we found both stabilized training and improved performance with minimal computational overhead. We used a dropout (60) of 0.5 between the convolutional layers in the residual blocks, as recommended in prior work (64). Before the residual blocks, the feature extractor used a single convolutional layer with a kernel size of (4, 39), a stride of (1, 10) along and 64 output channels. All the residual blocks used 64 input and output channels, a kernel size of either (1, 20) or (1, 19) and a stride of (1, 10) in the first convolutional layer and when downsampling the identity. The feature size after the convolutional blocks was 576, which went through BN-ACT-FC layers with an output feature size of 256. The feature extractor of the GLN model was similar to that of the CNN model, where the main difference was that we used LCLs instead of convolutional layers, only two residual blocks instead of four and no SE blocks. In the first LCL, we used a kernel width of 8 (covering two SNPs per group) and 4 output sets and in the subsequent residual layers, we used a larger kernel width of 32 and 4 output sets. The final output dimension from the feature extractor was 396. The tabular feature extractor used in all models used embeddings for categorical inputs and left continuous inputs unchanged. The tabular inputs were concatenated and passed through a single FC layer. The fusion model aggregated the intermediate representations from the individual feature extractors by simply concatenating them. For the CNN and GLN NN predictors, we used the fused features from the fusion module as input and propagated them through FC residual blocks. For the CNN and GLN models, the predictors used four residual blocks with 256 nodes in the FC layers and a dropout of 0.5 between the FC layers. After the final residual blocks, there was a BN-ACT-DO-FC which computed the final output for a given task. In the MLP case, we did not use residual blocks, but rather a classic feed forward network. The intermediate representation from the fusion model was propagated thorough five sets of BN-ACT-DO-FC layers. Excluding the last, all FC layers had 256 output nodes. We used a dropout of 0.5 before the FC hidden layers.

### A locally-connected layer for genome-wide data

For benchmarking the ability of the different candidate models to capture additive and non-linear XOR (interaction) effects, we analyzed how the models performed on simulated genotype data. Our primary aim with this simulation was to assess the models’ capabilities to handle additive and interaction effects, rather than to fully emulate the complexities of real-world genetic prediction. Here, we simulated three types of genotype-target relationships, with the target being a continuous value. The first set was a purely additive relationship, the second a mix of additive and interaction effects, and the third set purely interaction effects. Each simulation generated 12 000 simulated samples with 1000 simulated SNPs each. As expected, the MLP and CNN models were able to capture and model non-linear interaction effects with *R*^2^ of 0.95–0.98. However, the linear LASSO model had an *R*^2^ of 0.75 for a mix of additive and XOR effects, and completely failed to model pure XOR effects with an *R*^2^ of –0.03 (Supplementary Table S1). When scaling the NN based models to genome-wide genotype data or even to whole-genome sequencing data, the number of parameters when using fully connected (FC) layers increases dramatically. For instance, an FC layer with an input of 1 million one-hot encoded SNPs (i.e four elements per SNP) would require roughly 400 million weights to be connected to a hidden layer of 100 neurons. While convolutional layers can be much more parameter efficient, the computational complexity of training them on very high dimensional inputs can rival or exceed that of FC layers (46). Therefore, to have a model that was both parameter efficient and could take advantage of the local positional variance in genomics data, we implemented a locally connected layer (Supplementary Figure S3). The layer was sparsely connected through groups, which greatly reduces the number of parameters in comparison to an FC layer. The sparse connection allows for a larger intermediate representation while still keeping the parameter count relatively low. The GLN model was composed of multiple LCLs, and as was the case with the MLP and CNN models, it effectively captured both additive and non-linear effects in the simulated data (*R*^2^ = 0.98) while using fewer parameters (1.6× and 5.1× fewer than CNN and MLP respectively). To compare the CNN, MLP and GLN based models on real data, we performed a random neural architecture and hyperparameter search. For this comparison, we used type 1 diabetes, type 2 diabetes, atrial fibrillation and flutter, and hypothyroidism to compare CNN, MLP and GLN based models. For each model-trait combination, we performed 25 random samples of relevant parameters (such as kernel width for CNN models, first hidden layer size for MLP models, dropout for all models) and examined validation performance in ROC-AUC. Here, we found the GLN based architectures to perform overall favorably compared to the MLP and CNN-based models (Supplementary Figure S4 and Supplementary Data 1). While the MLP models performed markedly worse, the differences between GLN and CNN models were less pronounced. The CNN models had an average slight advantage for atrial fibrillation and flutter (ROC AUC 0.010), GLN models performed better for the other three traits (from ROC AUC 0.0055 for hypothyroidism to 0.016 for type 2 diabetes)). Notably, the best performing runs for each trait were all from GLN based models, with the greatest improvement being a gain of 0.036 ROC AUC for type 2 diabetes compared to the best performing CNN model. Thus, our results suggest that the GLN model offers an advantage over the CNN model implementation for certain traits, and the advantage is relatively robust across various combinations of hyperparameters.

### GLN was fast and robust to missing data

To simplify calculation of PRSs we, as mentioned above, implemented the models, including the LASSO, to automatically handle missing genotype data and thus removing the need to impute data before training. The genotype data was not pre-processed extensively before modelling. To investigate whether our results were consistent when using traditional pre-processing, we also trained GLN and LASSO on QC data. Besides reducing the number of SNPs and samples considered, the QC approach additionally resulted in a different train/test split. The NO-QC approach gave slightly better results on our eight benchmark traits (Supplementary Figure S5 and Supplementary Data 2). The overall trends were consistent whether using QC or NO-QC, e.g. with GLN performing markedly better on T1D (Supplementary Figure S5). For computational complexity, training the GLN model was slightly faster (32 h) compared with LASSO (34 h) for the eight benchmark traits (Supplementary Figure S6 and Supplementary Data 3). Even though the training latency of the LASSO model was lower than any of the NN based models, the total training time was higher due to using more steps before model convergence. Therefore, the framework was able to train large and deep neural networks on high dimensional individual-level genotype data in a reasonable time.

### Benchmarking with other PRS prediction tools

The bigstatsr training was done using a 5-fold cross-validation using a grid search α = [0.0001, 0.001, 0.01, 0.1, 1] for the elastic net mixing parameter, and the tool additionally tests various values for the λ penalization parameter. The tool then performs an ensemble-like procedure across the folds to produce the final model, which is evaluated on the test set. For the snpnet-2.0 benchmarking, SN-LASSO, SN-EN and SN-RIDGE refer to models trained with Lasso, Elastic Net and Ridge penalization respectively. All snpnet-2.0 models were trained with 2000 SNPs per batch, 100 iterations, 20 λ values in the first iteration, 10 extended λ values and a convergence threshold of 1 × 10^−7^. snpnet-2.0 training was first performed on the training set to find an optimal λ penalization parameter, and then refit on the training and validation set together using the found optimal λ value. Finally, the refit models were evaluated on the test set. For Asthma, the snpnet-2.0 Ridge regression model did not finish in the allotted 24 h runtime, and was therefore omitted from the results. The GLN training was done by using 10-fold Monte Carlo cross validation with the same model configuration for each fold. To get the final GLN results, an ensemble across all folds was performed. All models were adjusted for age, sex, and the first 10 genomic principal components (PCs).

### Replication in the Danish Blood Donor Study

To examine how transferable the trained DL models were between cohorts, we trained GLN-based DL models on the UKBB and used them to predict into the Danish Blood Donor Study (65). We used 64 827 autosomal SNPs that were present in both cohorts and three continuous (height, body mass index, lipoprotein(a)) and two categorical (type 2 diabetes, hypertension) phenotypes for the analysis. The covariates age, sex and genotype principal components were not used in this analysis, only genotype data. We used 488 263 individuals from the UKBB for training and validation, and the trained models applied to predict phenotypes for 99 704 participants in the DBDS external test set. In the DBDS, the case count for type 2 diabetes and hypertension was 1640 and 2728 respectively. For each phenotype, a 5-fold Monte Carlo cross validation was performed within the UKBB and an ensemble prediction performed in the DBDS.

### Multi-task prediction

We use multi-task to describe when we are predicting more than one disease diagnosis at the same time. In the setup of our experiments, this is very similar, and one could say conceptually the same as multi label prediction (where we are predicting multiple target values, where a sample can be assigned multiple labels at the same time, i.e. the labels are not mutually exclusive). However, we do use the term ‘multi-task’ here for a couple of reasons. Firstly, each task (i.e. disease diagnosis) is assigned a separate NN ‘head’ (here ‘head’ refers to a set of neural network layers specific for an output) that propagates the final fused hidden state to a prediction for that task. Secondly, each task is assigned a specific loss module (i.e. calculated according to binary cross entropy for categorical targets), for which the task loss weights are dynamically set according to the homoscedastic uncertainty of each task (66). For each task, the NN predictor was a sequence of four residual blocks with FC layers composed of 256 nodes in the two and eight task models, but 64 nodes in the 338 task model. The technique we use for our MT learning is known in the as hard-parameter sharing, where all tasks share a subset of the model parameters throughout the entire training procedure. To examine how well the default GLN model performed in MT learning compared to other NN models, we compared it with an MLP model and a GLN based model using a Multi-gate Mixture-of-Experts (MGMoE) (67) as the predictor on the 8 benchmark traits. We found the default GLN model to perform the best overall (Supplementary Figure S7 and Supplementary Data 4).

### Main and interaction effect identification

To examine the effects between SNPs, we used the 200 most highly important SNPs (according to average absolute SHAP values for each SNP on the validation set) by the GLN model as candidates for the analysis. Using those SNPs as inputs, we trained a gradient boosted decision trees model using the XGBoost framework (68). Tree-based models have previously been successfully applied in the context of PRS prediction (31,32). Both tree and DL-based models automatically handle missing genotype values, which can be advantageous when modelling on diverse populations, where missing genotypes cannot be reliably imputed. The trained decision trees used a learning rate of 0.002, maximum depth of 6, 10 000 boosting iterations and a 50% training set subsample for each boosting iteration. The same training, validation and tests sets were used as for the GLN model training and evaluation. After training, we subsampled a maximum of 2000 samples per class in the test set for the main and interaction effect analysis, for which we computed the SHAP effect values for analysis.

## RESULTS AND DISCUSSION

### GLN based genome-wide polygenic models can outperform linear models in the UKBB cohort

**Figure 1.**
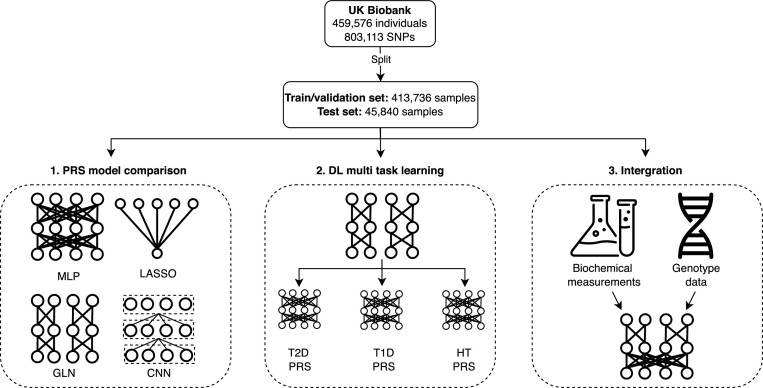
Study overview. A diagram showing the high-level steps taken for the study. First, deep learning models (MLP, CNN, GLN) were compared with linear methods (LASSO) to examine their feasibility in PRS prediction. Second, a single DL model trained to predict up to 338 disease traits at the same time from large-scale genotype data. Finally, DL models were used to integrate biochemical and genotype data for prediction.

**Figure 2.**
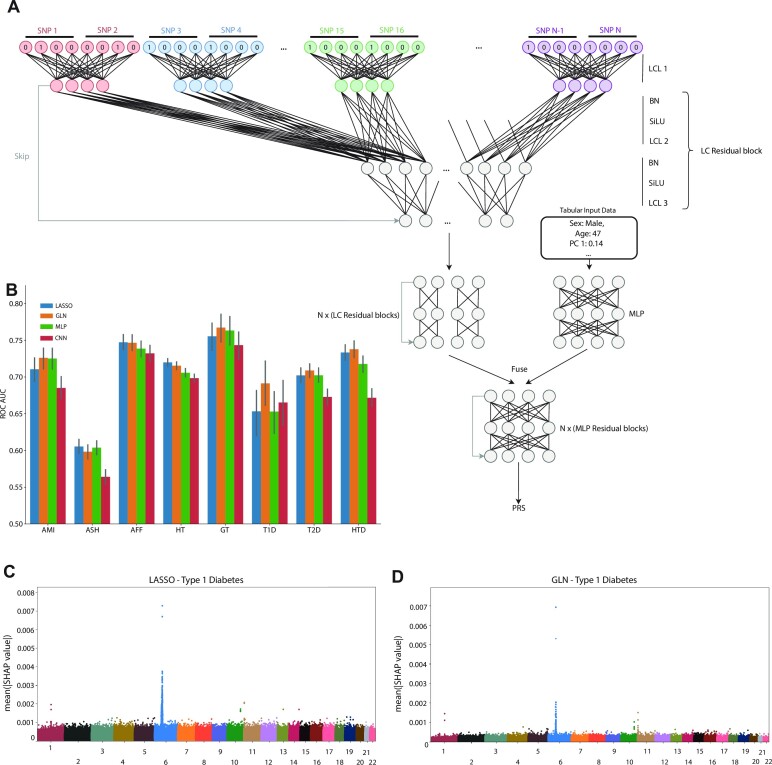
Genome-local-net (GLN) model architecture and performance. (**A**) Model architecture. The model uses a locally connected layer (LCL) with a kernel width covering two SNPs and four weight sets as the first layer. The output of the first layer subsequently goes through residual blocks composed of LCLs. The genomic representation is then fused with the tabular representation, which then is propagated through FC based residual blocks. A final set of BN-SiLU-FC layers is used to compute the final output. (**B**) Comparison of LASSO (blue), GLN (orange), MLP (green) and CNN (red) performance on the test set across eight traits in AUC-ROC. All models were adjusted for age, sex, and the first 10 genomic PCs. Bars represent the 95% CI from 1000 bootstrap replicates on the held-out test set. AMI: acute myocardial infarction, ASH: asthma, AFF: atrial fibrillation and flutter, HT: hypertension, GT: gout, T1D: type 1 diabetes, T2D: type 2 diabetes, HTD: hypothyroidism. (**C**) SNP feature importance distribution for type 1 diabetes for LASSO, showing high importance in and around the HLA region. The importance values represent average absolute SHAP values, and were aggregated across 10 randomly seeded training runs, computed on the validation set. Each point represents a variant, and the points are colored according to chromosomes. (**D**) SNP feature importance distribution for type 1 diabetes for GLN, showing high importance values in and around the HLA region. The importance values represent average absolute SHAP values, and were aggregated across 10 randomly seeded training runs, computed on the validation set. Each point represents a variant, and the points are colored according to chromosomes.

The analysis and results in this study can be divided into three major themes. Firstly, we examine the feasibility of training and interpreting DL models on high dimensional human genotype data, and compare them to linear models. Secondly, we explore to what extent DL models can be used for multi-task PRS prediction. Finally, we investigate the effect of integrating biochemical measurements with genotype data using DL for PRS prediction (Figure 1). When developing our DL models, we first established that the NN-based multilayer perceptron (MLP) (69) and convolutional neural network (CNN) (70,71) models were able to capture non-linear effects on simulated genotype data, whereas the LASSO model, as expected, could not (sec Materials and Methods). Additionally, we found our GLN model, which was composed of multiple LCLs (Materials and Methods and Figure 2A), to effectively capture additive and non-linear interactions (Supplementary Table S1). We then trained and validated PRS models using LASSO, MLP, CNNs or the GLN model for eight selected traits on 413 736 individuals with British, Irish, or other Western European background in the UKBB cohort (Figure 2B, Supplementary Figure S8 and Supplementary Data 5). Interestingly, we found that GLN was superior to using our LASSO model for T1D, with an improvement of 0.04 ROC-AUC on a held-out test set (MATERIALS AND METHODS). For the remaining traits, the differences were equal or less than 0.01 ROC-AUC. Additionally, the GLN had better performance compared to the MLP and CNN with average improvements of 0.01 and 0.03 ROC-AUC, respectively. This replicates previous results where CNN-based models did not show a consistent advantage for human trait prediction (34). To evaluate the DL architectures further, we used random neural architecture search and found the GLN based architectures consistently performed better than CNN and MLP based ones (Supplementary Figure S4 and Supplementary Data 1). To examine whether the gain of 0.04 ROC-AUC for T1D was due to the chosen hyperparameters for the LASSO model, we retrained the LASSO with various combinations of hyperparameters but did not find it to match the performance of the GLN model (Supplementary Table S2). To verify this even further, we compared the performance of the GLN model with bigstatsr (20) and snpnet-2.0 (21), both state-of-the-art methods for fitting additive models on individual-level genotype data. Here, we found bigstatsr and snpnet-2.0 to outperform the GLN model for 6 out of 8 traits when it came to ROC-AUC, although only with an average difference of 0.006 and 0.008 respectively (Supplementary Figure S9 and Supplementary Data 6). The ROC-AUC performance difference was in line with our results, where we did not see a strong trend of the NN models outperforming our LASSO, indicating that there might not be strong non-linear effects for these traits. Therefore, a model that assumes additive effects and is highly optimized to model on such effects is expected to perform favorably on those traits, compared to a complex DL model. However, we did find that we could replicate our results for T1D, where the GLN outperformed both bigstatsr and snpnet-2.0, strongly indicating that the GLN was able to identify and effectively using non-additive effects for prediction. To investigate and explain what the models had learned, we determined the SNPs that had the highest SHAP (72) effects and cross-referenced them to known associations for a particular trait. Specifically, for the T1D model we found that both LASSO and GLN assigned high importance values to SNPs in the HLA region of chromosome 6 (Figure 2C, D)—a region that has previously been associated with T1D (73). Furthermore, SNPs on chr11 (INS), chr1 (PTPN22) and chr10 (TCF7L2) had high feature importance values in both models (Figure 2D, Supplementary Figure S10 and Supplementary Tables S3 and S4). Examining the genotypic effects of the highly important SNPs, we found examples of both additive and non-additive effects. For example, for chr14 SNP rs2102484, the main effects of the heterozygote and homozygote alternative were opposite, with the CT genotype decreasing risk and the TT genotype increasing risk, an effect which purely additive models are not expected to capture. This pattern was not limited to this SNP only, as among the top 20 important SNPs, we found more examples (5 out of 20) of such a non-additive effect for T1D (Supplementary Figure S11). To examine this further, we fit a logistic regression model on these SNPs, where each genotype was assigned a separate parameter (i.e. non-additive encoding) and found them to match the effects closely (Supplementary Table S5). For example, the CC, CT and TT genotype of rs2102484 had odds of 0.082, 0.071 and 0.84 respectively. These results indicate that non-additive relationships are present in the UKBB between genotypes and some disease traits, and it is likely one effect that DL-based models capture which improves performance over additive models.

### GLN identifies disease relevant variants

When expanding the feature importance analysis to the other 7 traits, we found that in all cases a known association was found among the top 20 SNPs or the genes they reside in (Supplementary Figures S12–S19 and Supplementary Tables S3 and S6–S12). This is a strong indication of the models learning biologically relevant associations and that complex neural networks can be interpreted when modelled on extremely high dimensional genotype data. Even for diseases such as Acute Myocardial Infarction and Gout where the covariates alone (age, sex, and first 10 genotype principal components (PCs)) had a better performance compared with genotype and covariate data (Supplementary Figure S20 and Supplementary Data 7), we found that the GLN model was assigned high feature importance to numerous relevant SNPs and genes for both diseases (Supplementary Tables S6 and S10). The better performance of the covariate based models could be due to the covariates having much larger effects than the genotyped SNPs, e.g. if a disease was strongly affected by age or population stratification. Including the high-dimensional genotype data could increase overfitting, which then inflicts a performance trade-off against the much lower dimensionality of using only the covariates. Hence, a higher case count might be required to capture the SNP effects to such a degree that it boosts performance over the covariate based models (74).

### Transferability of GLN based PRSs across ancestries and cohorts

We evaluated the performance of the GLN model when trained and applied to a mixed population of individuals born in the UK and other countries (Supplementary Figures S21, S22, Supplementary Data 8 and 9). Our results indicated that the model generalized well for disease-ancestry combinations with a high sample count, such as hypertension prediction in individuals of African origin. However, for some other combinations, the results were mostly inconclusive, such as acute myocardial infarction in individuals of South American origin. This may be attributed to the limited number of origin countries apart from the UK in the UKBB study. For some combinations, the low case count causes large uncertainty in the performance and possible model overfitting on UK genotypes, which transfers poorly to more genetically distant samples (75). Finally, we evaluated the ability of GLN models trained on UKBB data to accurately predict phenotypes in another cohort, the DBDS. By utilizing a set of 64 827 autosomal SNPs common in both cohorts and no other covariates, we observed a slight reduction in performance (–0.010 to –0.027) for both quantitative and binary traits evaluated (Table 1). This suggests that the GLN-based models trained on the UKBB exhibit accurate transferability to the DBDS and potentially other populations.

**Table 1.** Transferability of GLN DL models trained on the UKBB and tested on 99 704 individuals in the Danish Blood Donor Study (DBDS). A set of 64 827 common autosomal SNPs between the two cohorts were used for the training and testing. Only genotype data was used for the training and prediction, meaning that age, sex and genomic principal components were not included at any stage. For quantitative phenotypes the Pearson correlation coefficient (PCC) was used, while ROC-AUC was used for binary phenotypes. **BMI:** body mass index, **LPA**: lipoprotein(a), **T2D**: type 2 diabetes, **HT**: hypertension

	Height	BMI	LPA	T2D	HT
Cohort	(PCC)	(PCC)	(PCC)	(ROC-AUC)	(ROC-AUC)
UKBB	0.2969	0.2294	0.5848	0.6042	0.5664
DBDS	0.2847	0.2193	0.5586	0.5768	0.5487

### Improved PRSs for autoimmune diseases

Knowing that the GLN model was competitive with the LASSO implementation on the eight traits, we applied a more data-driven approach of training GLN, LASSO and two covariate based models on 338 binary disease traits with at least 1000 cases in the UKBB cohort (Supplementary Data 10). Among the four models tested, the GLN performed best on 58 diseases (17% of the total traits), whereas the LASSO model performed best on 44 diseases (13% of the total traits). Comparing the ROC-AUCs between GLN and LASSO, we found GLN to perform better overall (Wilcoxon signed-rank test, one-sided, *P* = 4.9 × 10^−14^). Interestingly, using only covariates had the best performance for the remaining 236 traits (70% of the total traits), and overall, it performed better when compared to GLN (Wilcoxon signed-rank test, one-sided, *P* = 4.2 × 10^−15^ and *P* = 0.0013 for linear and NN based covariate models respectively) (Supplementary Data 11). The covariate based models performing better could be due to the low effective sample size (ESS), overfitting by the genotype models and the nature of some traits being more driven by environmental factors (Supplementary Figures S23 and S24). When filtering disease traits for where GLN and LASSO had better performance compared with covariates and difference of at least 0.01 ROC-AUC, we found 16 and 9 disease traits where GLN and LASSO had the best performance, respectively (Supplementary Figures S25 and S26). Interestingly, the GLN model performed markedly better on T1D, rheumatoid arthritis, multiple sclerosis, psoriasis and ulcerative colitis, all autoimmune traits in which studies have shown indication of interaction effects (49–50,76–80). For instance, for rheumatoid arthritis, the GLN model had a ROC-AUC of 0.664 while the LASSO had a ROC-AUC of 0.624 on the test set and the covariate only models achieved a ROC-AUC of 0.622 and 0.634 for the LASSO and NN based models, respectively (Supplementary Figure S27). When examining GLN and LASSO feature importance for rheumatoid arthritis, we found, as above, the models assigned high importance to relevant SNPs (Supplementary Figures S28 and S29 and Supplementary Tables S13 and S14). Taken together, our results therefore show an improvement of using NNs compared to LASSO for predicting disease risk from genome-wide genomics data.

### GLN identifies SNPs with widespread interaction effects

**Figure 3.**
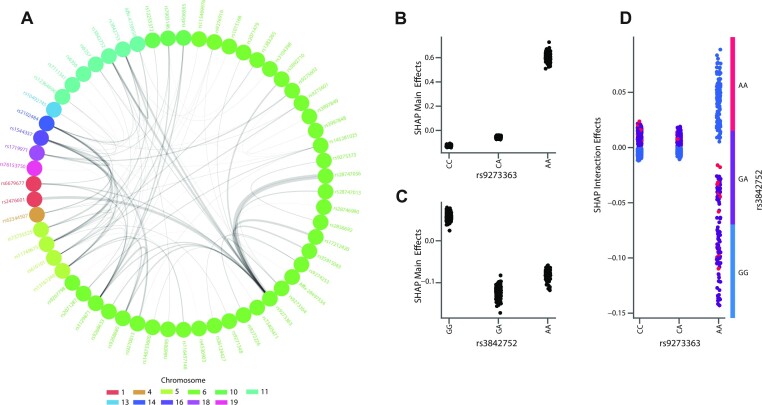
Interaction effects among highly important SNPs for type 1 diabetes (T1D). (**A**) A network showing the interaction between different SNPs for T1D. The top 200 important SNPs (according to average absolute SHAP values computed on the validation set) across 10 training runs with the GLN model were studied for interaction effects between them using gradient boosted decision trees (GBDT). The 200 strongest interaction effects among all SNP combinations are plotted in the graph. Each node represents a variant, and the edge widths represent the strength of the interaction between the connected variants. The node colors represent which chromosome variants reside in. The network shows particularly strong interaction effects between various SNPs on chr6, but also widespread interaction effects between chromosomes. (**B**) Main effects of chr6 SNP rs9273363 on T1D, with the AA genotype having a strong effect in increasing risk. The y-axis values represent the main effect influence of a given rs9273363 genotype on the trained GBDT model output logits. (**C**) Main effects of chr11 SNP rs3842752 on T1D, with the GG genotype having a moderate effect in increasing T1D risk. The y-axis values represent the main effect influence of a given rs3842752 genotype on the trained GBDT model output logits. (**D**) Interaction effects between chr6 SNP rs9273363 and chr11 SNP rs3842752. The x-axis represents the rs9273363 genotype, the y-axis represents the interaction effect influence on the trained GBDT model output logits and the colors represent the GG (blue), GA (purple) and AA (red) genotypes of rs3842752. The vertical dispersion seen for the AA genotype of rs9273363 indicates that genotype combinations explored have different effects for different samples. This can be due to other SNPs having an additional interaction effect on rs9273363 and rs3842752, which can be seen in Figure 2A where the SNPs not only interact with each other, but multiple other SNPs.

With results showing improved performance when using GLN for traits suggested having interaction effects, we decided to analyze the T1D SNPs important to the GLN model in more detail (see Materials and Methods). Using gradient boosted decision trees (GBDT), which previously have been used to identify nonlinear interaction effects (81), we identified the strongest 200 interactions spanning 11 different chromosomes. We found particularly strong effects within chr6 but also between SNPs on chr6, chr1 and chr11 (Figure 3A). In particular, we found the SNP rs9273363 located near HLA-DQB1 to have, as previously found, interaction with multiple other variants (82–85). One example was the interaction of rs9273363 with chr11 rs3842752 and rs3842753, which map to insulin (INS and INS-IGF2) and were in strong LD with each other. We focused on the interaction between rs3842752 and rs9273363 and found that individually, the AA genotype of rs9273363 (HLA-DQB1) and GG genotype of rs3842752 (INS) increased the model output towards a positive T1D prediction with effects of 0.6 and 0.1, respectively, whereas GA and AA of rs3842752 decreased risk (Figure 3B, C). Fitting a logistic regression model on the two SNPs with T1D as the target validated the direction of the main effects, with odds ratios (ORs) of 4.34 and 1.41 for rs9273363 and rs3842752 respectively (Supplementary Table S5). The ORs were close to those from a previous T1D study for the AA genotype of rs9273363 (OR 5.48) and the TT genotype of another INS SNP, rs3842727 (OR 1.53), which was in high LD with rs3842752 (*R*^2^ > 0.75) (86). However, when rs9273363 (HLA-DQB1) was homozygote for the risk genotype (AA) the presence of at least one protective genotype (GA or AA) of rs3842752 (INS and INS-IGF2) additionally decreased the risk of T1D (Figure 3d). This indicates that the GLN model was able to identify SNPs that have main and non-linear interaction effects, and that the interaction effects can be between chromosomes. Furthermore, we found the rs9273363 (HLA-DQB1) genotype to have the most high T1D ranking interactions. For instance, among the top 20 SNPs interacting most strongly with rs9273363, five of them were not on chr6. Of the 15 located on chr6, 10 were not in LD with rs9273363 (*R*^2^ < 0.1) and besides their own main effect modified the risk contribution of rs9273363 between 0.15 and –0.3 through interaction effects (Supplementary Figure S30). Examining the output distribution of the GBDT model, a value of 0.3 does have a relatively strong influence in shifting the model’s attributed risk for an individual (Supplementary Figure S31). Therefore, the total contribution across multiple interaction effects can have a strong influence in modulating the total risk of an individual, highlighting their importance for predictive modelling. Taken together, indicates a complex relationship between loci and genotypes in modulating T1D risk in the UKBB that can be discovered and modeled using EIR.

### Multi-task learning offers a trade-off between performance and complexity

**Figure 4.**
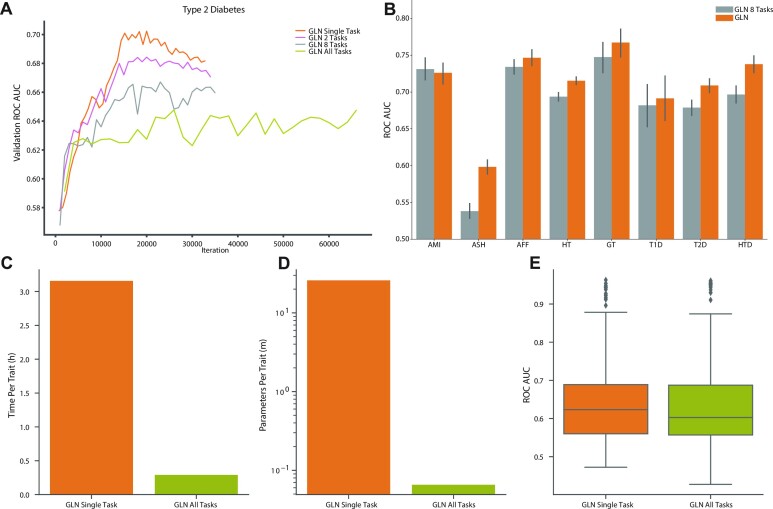
Genome-local-net (GLN) multi-task (MT) predictions. (**A**) Comparison of validation curves in ROC-AUC for type 2 diabetes (T2D) as more tasks are added alongside the single task type 2 diabetes prediction (orange). The two task model (pink) is trained jointly on T2D and hypertension. The eight task model (gray) is trained on the eight benchmark traits showed in Figure 2B. The model trained on all tasks (green) is trained on the total set of 338 diseases considered in this work. The single and two task runs show signs of overfitting, as validation performance peaks and starts to deteriorate around 20K iterations. The eight and all MT runs do not show as clear signs of overfitting, but overall performance is worse. (**B**) Comparison of single task (orange) and MT performance (gray) for the 8 benchmark traits on the held-out test set. Bars represent the 95% CI from 1000 bootstrap replicates on the held out test-set. AMI: acute myocardial infarction, ASH: asthma, AFF: atrial fibrillation and flutter, **HT:** hypertension, GT: gout, T1D: type 1 diabetes, T2D: type 2 diabetes, HTD: hypothyroidism. (**C**) Comparison of training time per trait for the all task MT model (green) and single task training (orange). (**D**) Comparison of number of parameters per trait for the all task MT model (green) and single task (orange) training. (**E**) Overall performance on the held-out test set of the all task GLN MT model (green) and single task (orange) training.

In multi-task (MT) learning, a single model is trained to solve multiple objectives at the same time, such as predicting height, disease liability and ethnicity. This can lead to improved predictive performance, reduced training time and better parameter efficiency (87,88). We therefore hypothesized that predicting multiple outcomes simultaneously could regularize and potentially improve prediction performance. Using type 2 diabetes (T2D) for comparison, we trained MT models to predict two, eight and 338 diseases jointly and found that maximum validation performance got progressively worse when increasing the number of tasks (Figure 4A and Supplementary Data 12). This indicates that the model capacity was not high enough to effectively capture the variance of multiple traits as well as the single task model, or that negative transfer between tasks degraded performance (89). Similarly, when comparing test set performance for the respective single task models and an MT model trained on the eight benchmark traits, we found that the MT model was slightly worse for seven diseases (average 0.024 ROC-AUC lower), with Acute Myocardial Infarction being the exception (0.0055 ROC-AUC improvement) (Figure 4B). However, despite being slightly worse for most of the traits, the MT models were remarkably effective. For example, the 8 trait MT model had a test ROC-AUC of 0.68 for T1D, which was considerably higher than the 0.58-0.59 ROC-AUC when using only covariates. To examine how well the framework scaled and whether we could effectively train very large-scale MT models, we trained one GLN model to jointly predict 338 traits simultaneously. As expected, modelling on all traits jointly significantly reduced the training time (11×) and number of parameters per trait (395×) (Figure 4C, D). As in the other MT experiments, this came at the cost of reduced performance compared to the single task setting (Wilcoxon signed-rank test, one-sided, *P* = 0.03), however only with an average difference of 0.0054 ROC-AUC (Figure 4E and Supplementary Data 13). Compared to the best performing covariate based models for each trait, the large MT model performed better for 63 traits, indicating that it was able to effectively capture genotype variance for some traits and not only using the covariates.

### Integrating genomics with clinical data improves predictive performance

**Figure 5.**
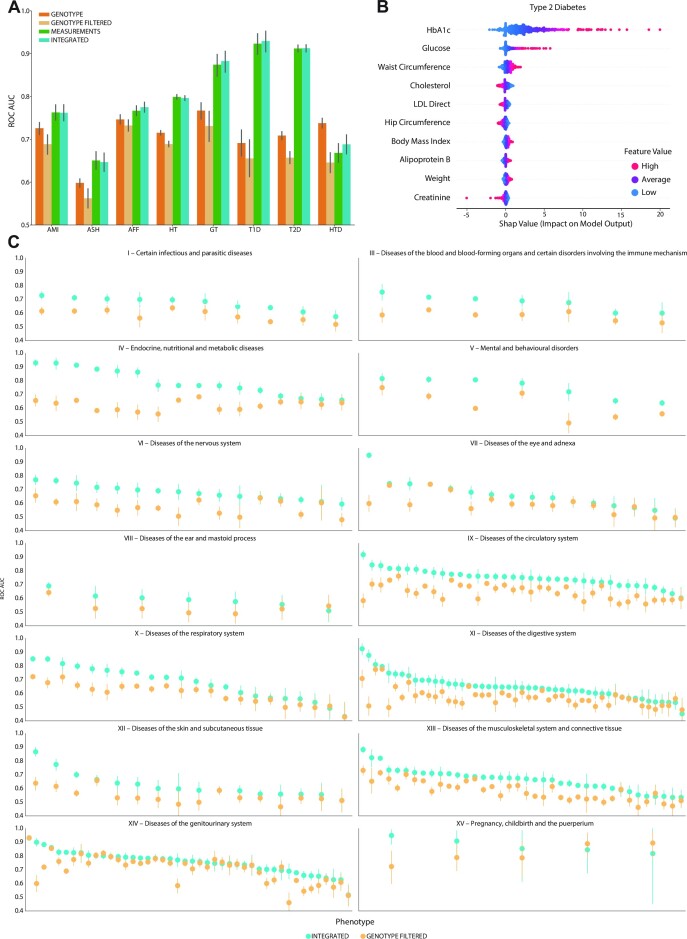
Integrating genotype and clinical data with genome-local-net (GLN). (**A**) Comparison of model performance using genotype (orange), genotype filtered (light orange), measurement (green) and integrated (teal) data in ROC-AUC on the held-out test set. Bars represent the 95% CI from 1000 bootstrap replicates on the held-out test set. AMI: acute myocardial infarction, ASH: asthma, AFF: atrial fibrillation and flutter, HT: hypertension, GT: gout, T1D: type 1 diabetes, T2D: type 2 diabetes, HTD: hypothyroidism. (**B**) Feature importance and impact of integration measurement values on the GLN model prediction for T1D. For a given feature, each dot represents a sample in the test set. The colors indicate the actual feature value. For example, a strong trend of high glycated haemoglobin value influencing the model to make a positive prediction for T2D can be seen. (**C**) Summary of ROC-AUC performance on the held-out test set across all the 290 traits that had a time measured column associated with them, with Integrated data (teal) compared with genotype filtered data (light orange), filtered for time of diagnosis. Each subplot represents an ICD10 chapter. Bars represent the 95% CI from 1000 bootstrap replicates on the held-out test set.

Although genetic data has proved to be a powerful predictor of various traits and diseases, there are other factors such as environmental effects that can play an important part (90). With the increased digitization in the healthcare industry, clinical and electronic health data is only expected to become more widely available. Among these are factors that are relatively easy and non-invasive to measure, such as anthropometrics, and other measurements included in the UKBB, such as blood and urine measurements. To examine the benefit of using these in our models we trained GLN models using only genotype data and covariates, and compared this with using genotype, covariates, physical, blood, and urine sample measurements (denoted ‘Integrated’). Additionally, to minimize feature leakage (see Materials and Methods), we filtered out samples where the diagnosis occurred before biochemical measurements. This is expected to reduce the likelihood of the model predicting previously diagnosed conditions, rather than future diagnoses. Therefore, when including the measurements the number of cases was for most traits reduced, leading to a trade-off between the gain of including measurements and the loss of removing samples. To examine this trade-off more precisely, we compared to two genotype datasets, one where the matching individuals (i.e. those that had biochemical measurements taken after disease diagnosis, in this case even though the measurements were not included as inputs) were removed (denoted ‘Genotype Filtered’), and another set where all individuals were included (denoted ‘Genotype’) (Figure 5A and Supplementary Data 14). For all eight benchmark traits, as expected, removing samples reduced performance with ROC-AUC of 0.014–0.092 (Wilcoxon signed-rank test, one-sided, *P* = 0.0039). Another contributing factor could be that sample removal was likely biased towards individuals with a high genetic load, and therefore diagnosed early. Compared to Genotype Filtered data, we found that using Integrated data greatly improved performance, with ROC-AUC increasing by 0.043–0.27 (Wilcoxon signed-rank test, one-sided, *P* = 0.0039) (Figure 5A). This was also the case when using MCC as metric, which improved between 0.010-0.35 (Supplementary Figure S32). The improved performance when including measurements was also reflected in prevalence plots, where T2D and hypertension both had >50% prevalence in the top PRS percentile (Supplementary Figure S33). However, compared to the unfiltered Genotype data, the results were more disease dependent. For instance, filtering hypothyroidism for time of diagnosis reduced case count from 16 894 to 4663 in the training set, which was reflected in ROC-AUC performance reduction of 0.091. Including measurements therefore did not outweigh the performance reduction of discarding cases. Interestingly, we found that using Integrated data had superior ROC-AUCs for five of the traits compared to using measurements and covariates only (denoted ‘Measurements’), highlighting the benefit of including genotype data.

### Integration of clinical and genomics data improve prediction of T2D

When investigating the feature importance of the integrative predictor, we found that the model was assigned high importance values to relevant clinical measurement features such as glycated hemoglobin (HbA1c) and blood glucose for T2D (Figure 5B). However, for some diseases, such as T2D, predictors using Measurements data and the Integrated data had very similar ROC-AUC performances. This does not necessarily indicate that the genetic component of the traits was low, perhaps the more likely explanation is that the measurements can act as a proxy for the genomics effects. For example, in the case of T2D, high genomics risk will in numerous instances manifest itself in high levels of glycated haemoglobin, and when it can be measured directly, there is perhaps not much extra variance gained when including the genotype data. However, as above, when we investigated the genotype feature importance, we found that the model assigned high importance values to relevant SNPs even when measurement data was included (Supplementary Figure S34 and Supplementary Table S15). While the ROC-AUC showed little difference, we found that including the genotype data resulted in a predictor for T2D with higher MCC (0.43) compared with using only the measurements (0.33) (Supplementary Figure S32). Interestingly, this was particularly due to better classification of true negatives and indicates the usefulness of the integration.

### Large scale integrative modeling

We then, as before, performed large-scale analysis of 290 traits that included time of diagnosis. Integration of the measurements showed a large increase in performance for almost all the traits compared with the Genotype Filtered predictor (Wilcoxon signed-rank test, one-sided, *P* = 4.5 × 10^−46^ and *P* = 9.2 × 10^−39^ for ROC-AUC and MCC, respectively) (Figure 5c and Supplementary Data 15). Interestingly, we had expected improvements for endocrine, nutritional and metabolic diseases but found improvements in ROC-AUC and MCC for other classes of diseases such as mental and behavioral disorders (Supplementary Figure S35). Compared with the Genotype predictors we observed the same overall trend that including measurements improved performance, but the effect was less pronounced due to the Genotype models using more samples (Wilcoxon signed-rank test, one-sided, *P* = 5.3 × 10^−37^ and *P* = 1.5 × 10^−08^ for ROC-AUC and MCC, respectively) (Supplementary Figures S36, S37 and Supplementary Data 15). To examine the effect of including genotype data when measurements were available, we compared the Measurements based models to models using Integrated data and found that the difference was small for most traits (Wilcoxon signed-rank test, one-sided, *P* = 0.044 and *P* = 0.053 for ROC-AUC and MCC, respectively) (Supplementary Figures S38, S39 and Supplementary Data 15). This could be due to low ESS for many of the traits, traits being driven more by environmental effects or high genomic risk being reflected in the measurements (Supplementary Figures S23, S24 and S40).

## CONCLUSION

Here, by developing DL models specifically for large scale individual-level genotype data, we show that they can achieve competitive performance for a wide range of diseases, and that the performance of these models can generalize over ancestries and cohorts. For some traits within the UKBB, they can outperform linear models, and the gain could be due to capturing non-additive and interaction effects. While interaction effects are often overlooked in PRS studies, we found them cumulatively to have surprisingly strong effects in some cases, e.g. 52% of the total effect of rs9273363 on T1D risk. Accounting for them can therefore provide better predictive performance for some traits and could lead to valuable biological insights. We expect that finding such complex effects will become more common in the future, especially with the development of larger, better phenotyped cohorts. Interpreting such associations should be done with care, however, as computational associations are not guaranteed to capture true biological effects (91). Interpreting complex machine learning and deep learning models is an active area of research and although current methods perform well in many scenarios, they are not always guaranteed to be correct (92–94). Here, we have only focused on SNP-SNP interactions in our analysis of interaction effects. A more thorough analysis, such as the extent gene-environment interaction contributes to non-linear model gain is an interesting avenue of research. Furthermore, while complex non-linear models can be used to uncover such effects and provide a relative comparison of their strength, once identified, linear methods could be used to explicitly model and quantify the effects. Additionally, we showcase the flexibility that DL architectures offer by training a single model to predict 338 disease traits at the same time with minimal loss in performance. An interesting research direction could be to examine MT learning with focus on related tasks (e.g. pleiotropic traits in the context of PRS prediction) and applying more recently developed MT learning NN architectures, which might yield better results compared to our approach.

We found clear advantage of integrating additional measurements with genotype data. However, if including the measurements poses a feature leakage risk and subsequent removal of samples, one must consider whether the trade-off in samples and additional features is acceptable. Nonetheless, we saw a strong indication that inclusion of measurements outweighed the removal of samples for many disorders. Future work includes comparing non-linear models such as NNs to linear models to examine to what extent non-linear effects in the clinical and genetic data together contribute to increased predictive performance. We only considered data from individual-level cohorts, but it will be straightforward to integrate PRSs from predictors trained using summary statistics or genome-wide data and addition of these could potentially improve performance. Finally, we only considered two input modalities, genotype and tabular data for integration. However, more types of health data, such as high-resolution imaging, multi-omics and electronic health data, will be commonly measured in the future. Therefore, the development of accurate predictors that can model on various types of data, whether structured or unstructured, will be important for achieving precision medicine in the future.

## DATA AVAILABILITY

The EIR tool is available on GitHub at https://github.com/arnor-sigurdsson/EIR and the current version has been archived at Zenodo (https://doi.org/10.5281/zenodo.7866205). Documentation and instructions for use are provided within the repository. The data underlying this article are available in the UK Biobank resource, at https://www.ukbiobank.ac.uk and the Danish Blood Donor Study resource, at https://bloddonor.dk/bloddonorstudiet/the-danish-blood-donor-study-eng/, which researchers can apply for access to.

## Supplementary Material

gkad373_Supplemental_Files
