# Supplementary material for: Deep integrative models for large-scale human genomics
Source: Nucleic Acids Res. 2023 May 24;51(12):e67. doi: 10.1093/nar/gkad373 (PMC10325897; doi:10.1093/nar/gkad373)
Supplement: gkad373_Supplemental_Files [file gkad373_supplemental_files.zip › ukbb_supervised_supplementary.pdf]

SUPPLEMENTARY INFORMATION

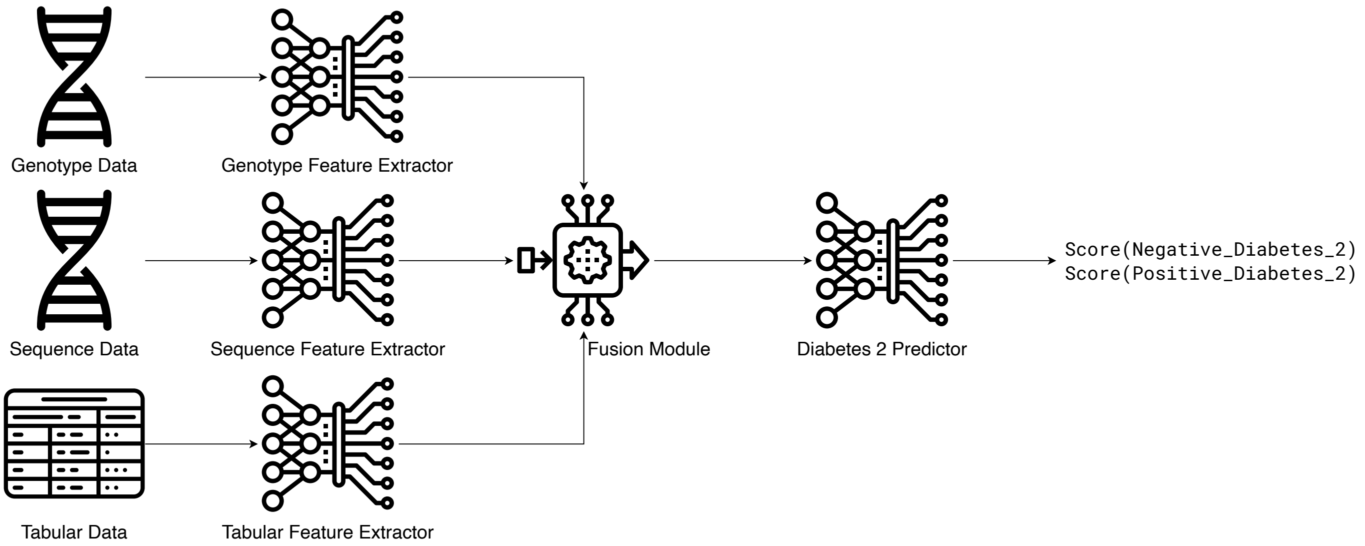

Supplementary Figure 1. Example overall architecture. Different sources and modalities can go through different NN feature extractors, which generate different intermediate representation for each input source. The representations get fused in a fusion module. The fused representation finally goes through a NN classifier, which generates the final prediction output.

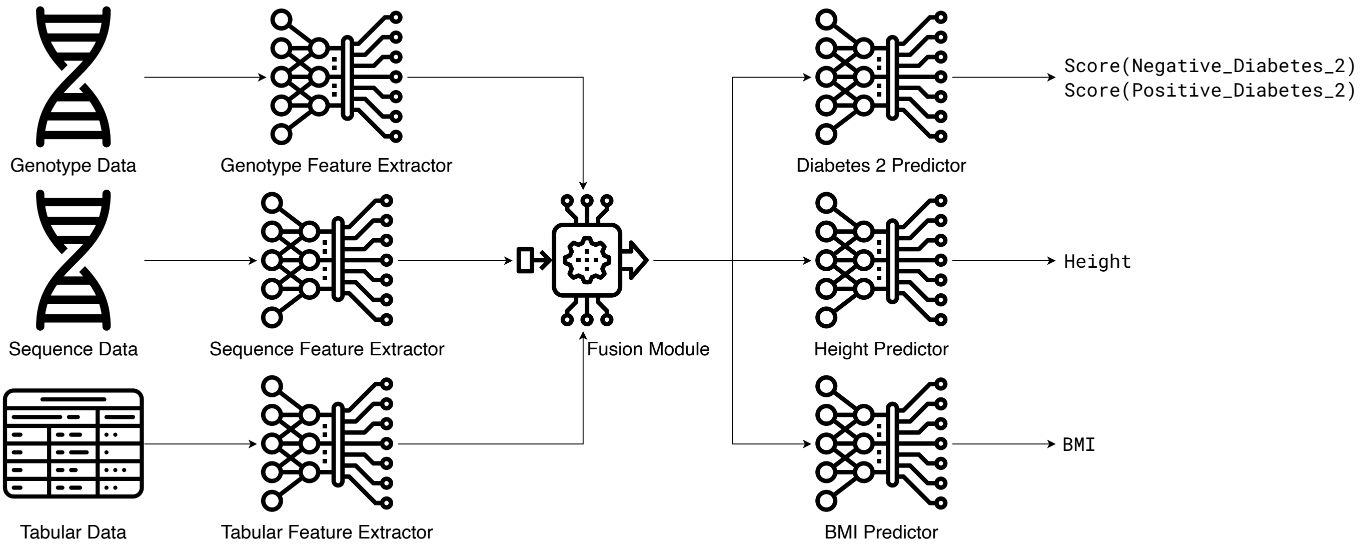

Supplementary Figure 2. Example overall architecture for multi-task learning. The figure is analogous to Supplementary Figure 1, but instead of predicting one output variable from the fused representation, it is passed to multiple predictors.

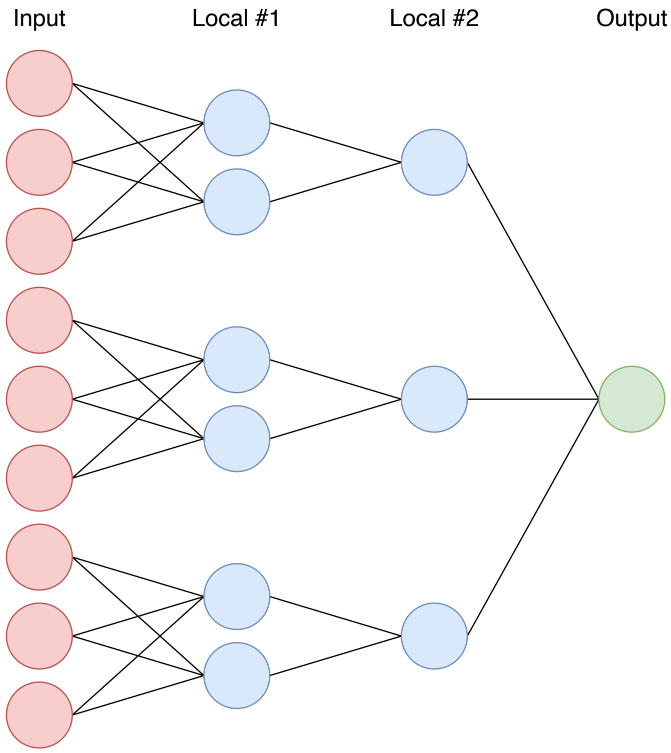

Supplementary Figure 3. Diagram showing how the locally-connected-layers (LCLs) are structured. The input (red) is connected to the first LCL layer, which has a kernel width of three in the first layer and two output sets, resulting in an intermediary representation with 6 nodes (light blue). The second LCL layer has a kernel width of two with one output set, resulting in an intermediary representation of 3 nodes (light blue). The intermediate representations from the second LCL go through an FC layer to generate the final output (green).

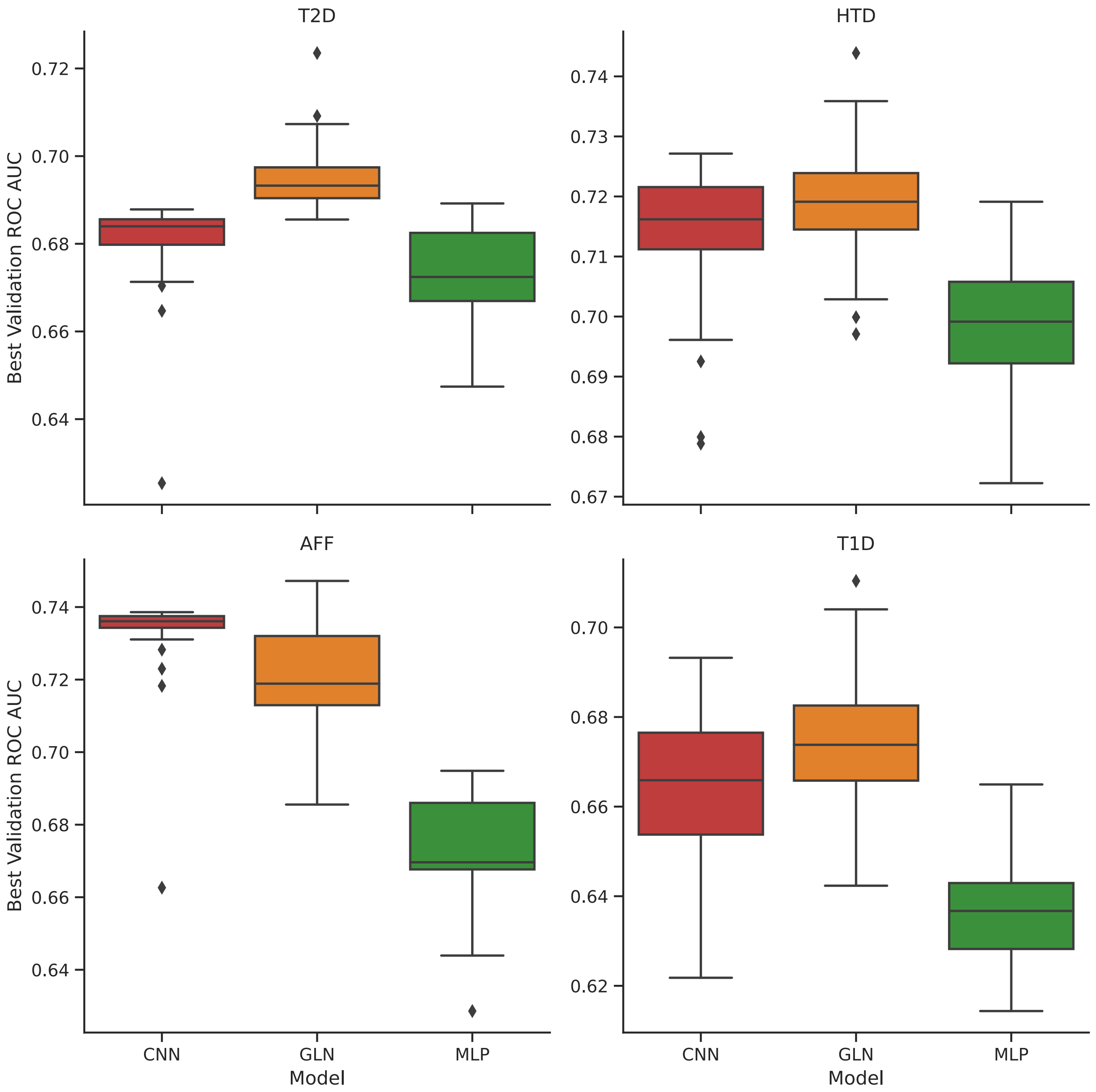

Supplementary Figure 4. Performance of CNN (red) GLN (orange), MLP (green) neural-network architectures, measured in ROC AUC. For each model type, 25 randomly sampled architecture combinations were trained and evaluated , and the iteration with the best performance on the validation set used. **T2D:** Type 2 diabetes, **HTD:** Hypothyroidism, **AFF:** Atrial fibrillation and flutter, **T1D:** Type 1 diabetes.

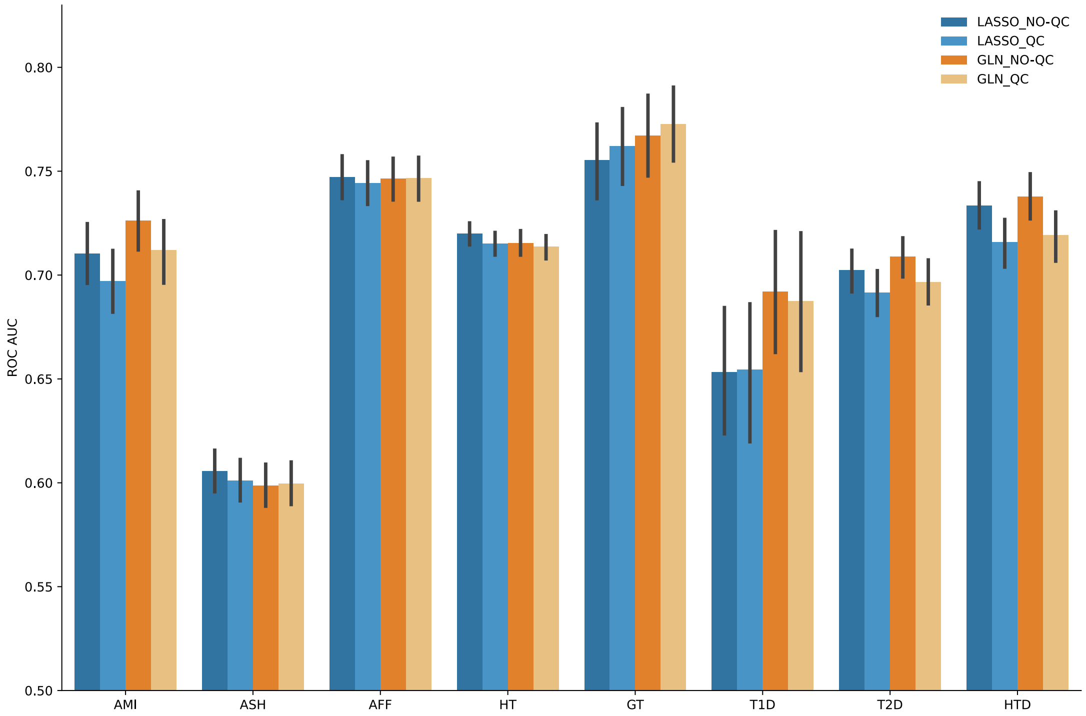

Supplementary Figure 5. Comparison of using traditional genotype pre-processing (QC) or not (NO-QC) (Methods) for 8 benchmark traits using the LASSO model trained with NO-QC data (blue), LASSO trained with QC data (light blue), GLN trained with NO-QC data (orange) and GLN trained with QC data (light orange). Bars represent the 95% CI from 1,000 bootstrap replicates on the held-out test set. **AMI:** Acute myocardial infarction, **ASH:** Asthma, **AFF:** Atrial fibrillation and flutter, **HT**: Hypertension, **GT:** Gout, **T1D:** Type 1 diabetes, **T2D:** Type 2 diabetes, **HTD:** Hypothyroidism.

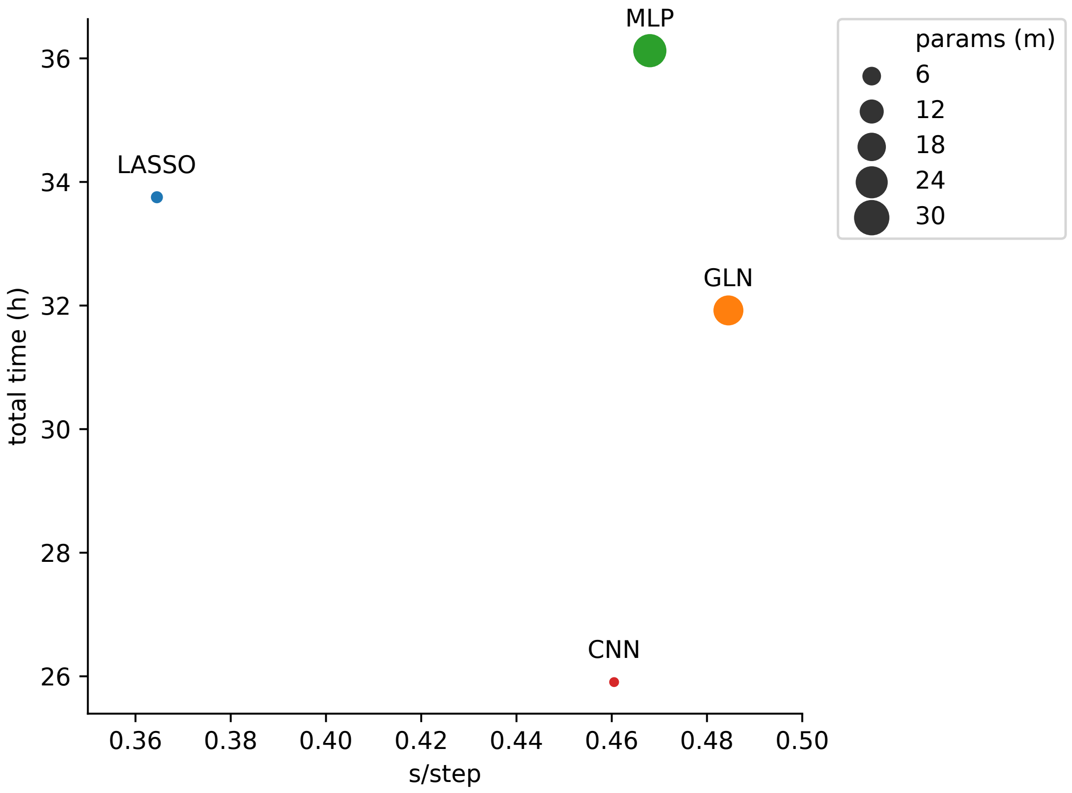

Supplementary Figure 6. Comparison of model total training time, training latency (seconds per batch of 64 samples) and number of parameters for the LASSO (blue), GLN (orange), MLP (green) and CNN (red) models. The y-axis represents total training time for all 8 benchmark traits, and the x-axis the time it took for a given model to do one forward and backwards pass of 64 samples. The point size is according to the number of model parameters.

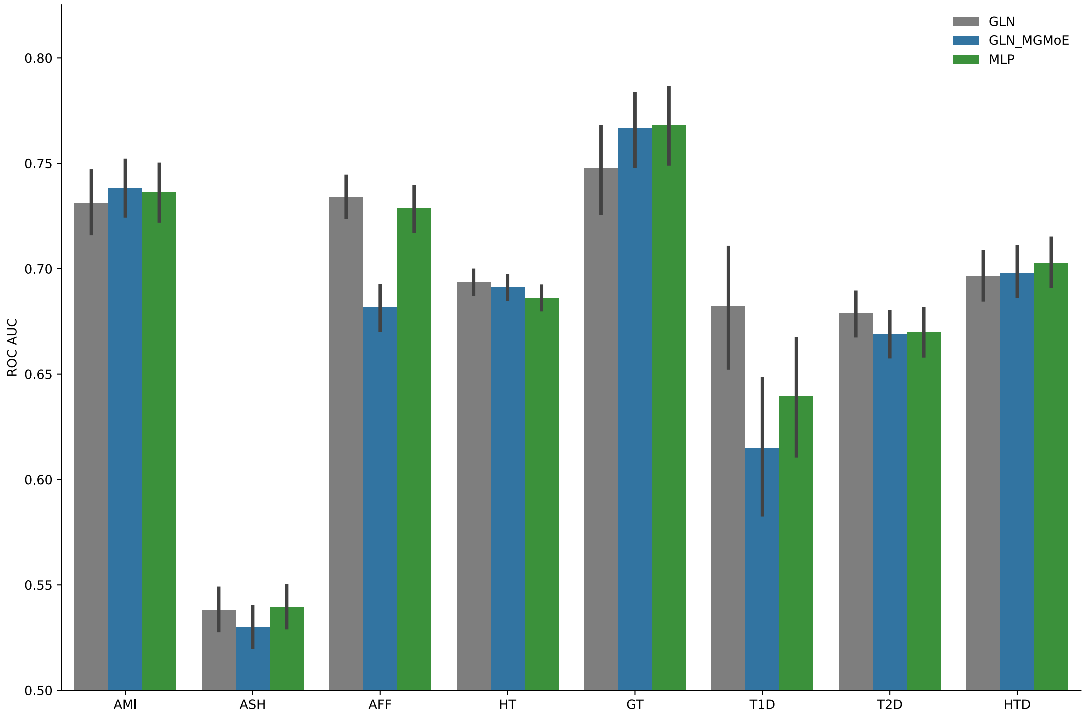

Supplementary Figure 7. Comparison of different MT architectures on the 8 benchmark traits in ROC AUC on the held-out test set. The GLN (gray), GLN_MGMoE (blue) and MLP (green) models had an average ROC-AUC of 0.688, 0.674 and 0.684 respectively. Bars represent the 95% CI from 1,000 bootstrap replicates on the held-out test set.

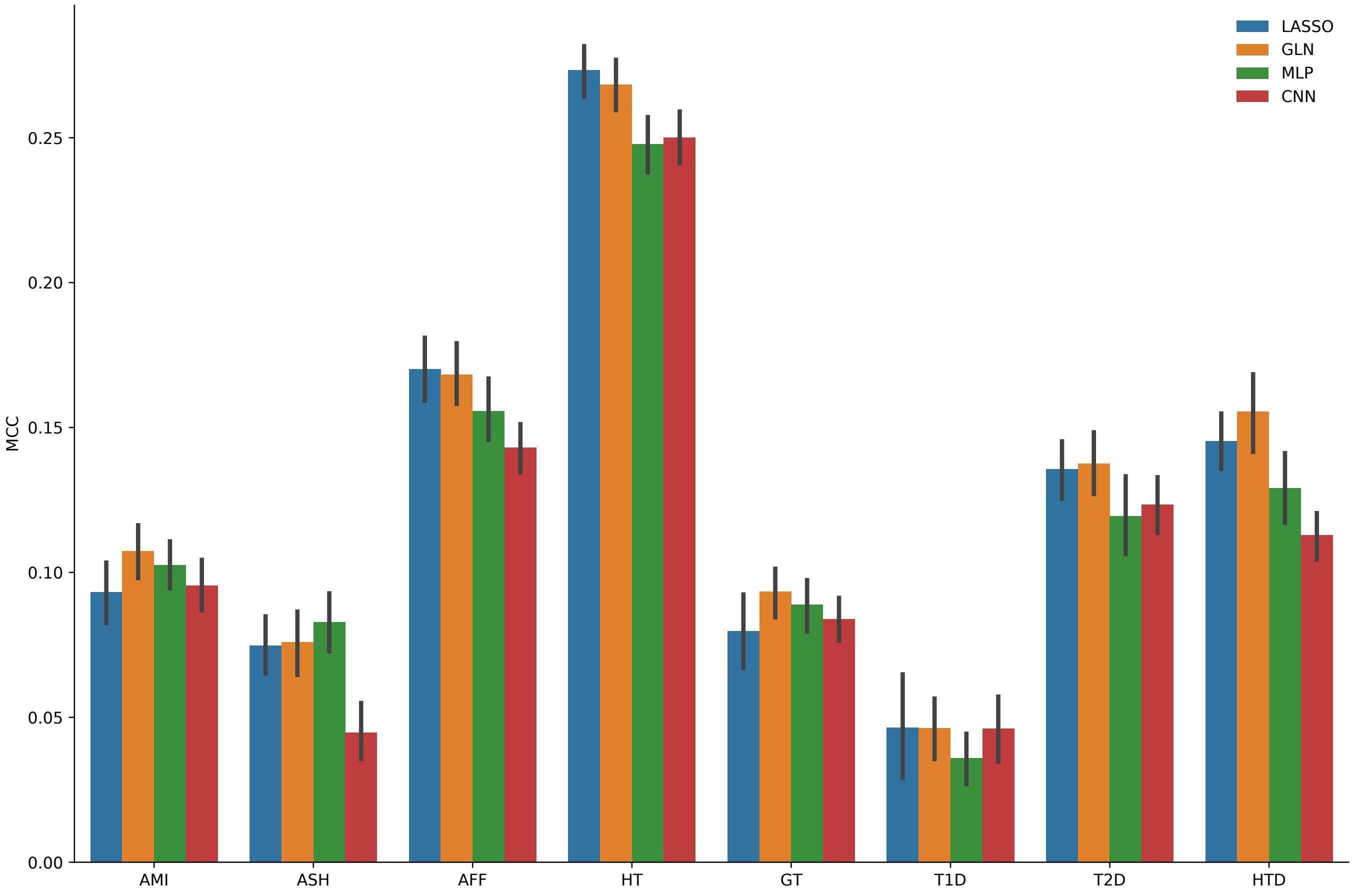

Supplementary Figure 8. Comparison of LASSO (blue), GLN (orange), MLP (green) and CNN (red) performance on the held-out test set across 8 traits reported in Matthews correlation coefficient (MCC). All models were adjusted for age, sex, and the first 10 genomic principal components (PCs). It should be noted that the model checkpoint used was the best performing one on the validation set using the ROC-AUC. Hence, there might not be a full correlation between the chosen model checkpoint being the one that performs best with respect to MCC. Bars represent the 95% CI from 1,000 bootstrap replicates on the held-out test set. **AMI:** Acute myocardial infarction, **ASH:** Asthma, **AFF:** Atrial fibrillation and flutter, **HT**: Hypertension, **GT:** Gout, **T1D:** Type 1 diabetes, **T2D:** Type 2 diabetes, **HTD:** Hypothyroidism.

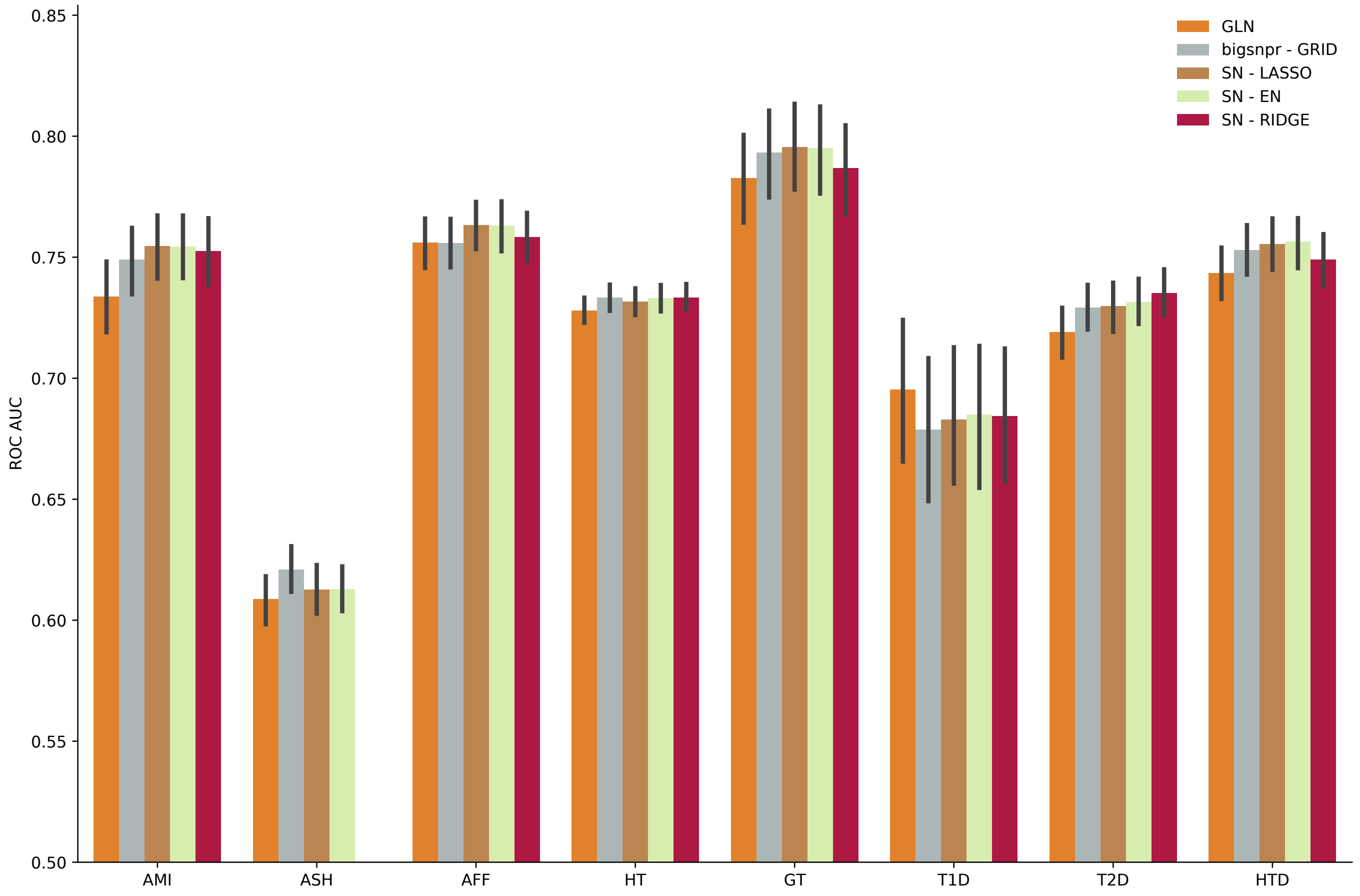

Supplementary Figure 9. Comparison of GLN (orange) and bigstatsr (gray) and snpnet-2.0 (brown, beige, and red) performance on the held-out test set across 8 traits reported in ROC AUC. All models were adjusted for age, sex, and the first 10 genomic principal components (PCs). Bars represent the 95% CI from 1,000 bootstrap replicates on the held-out test set. **AMI:** Acute myocardial infarction, **ASH:** Asthma, **AFF:** Atrial fibrillation and flutter, **HT**: Hypertension, **GT:** Gout, **T1D:** Type 1 diabetes, **T2D:** Type 2 diabetes, **HTD:** Hypothyroidism.

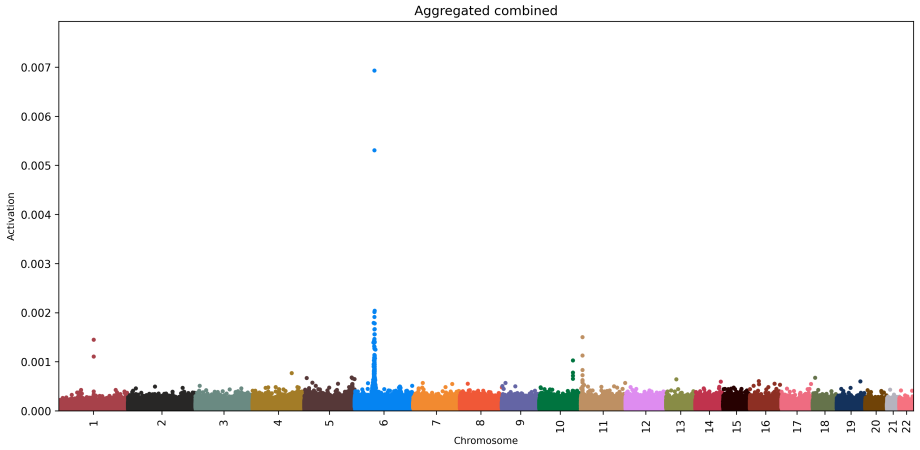

(a) Aggregated.

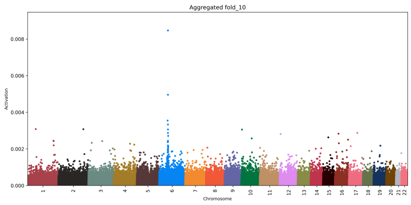

(b) Run 1.

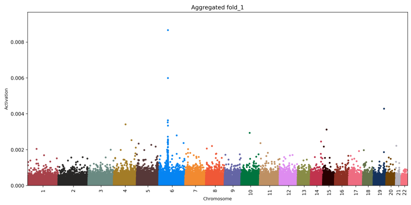

(c) Run 2.

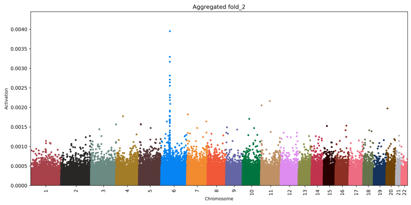

(d) Run 3.

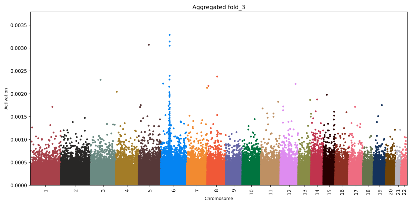

(e) Run 4.

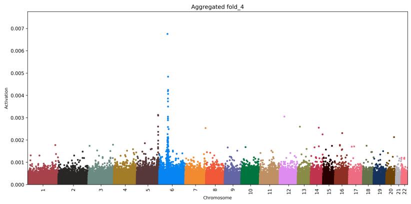

(f) Run 5.

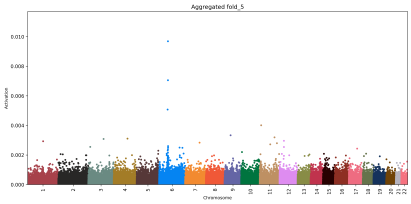

(g) Run 6.

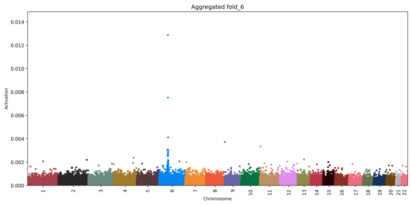

(h) Run 7.

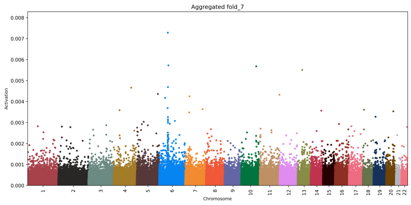

(i) Run 8.

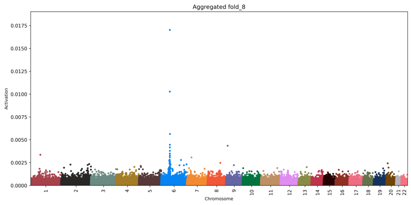

(j) Run 9.

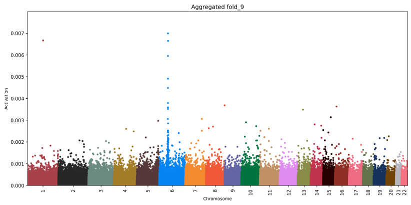

(k) Run 10.

Supplementary Figure 10. Aggregated and individual activations for type 1 diabetes (T1D) across 10 training runs with different random seeds when using the genome-local-net (GLN) model. The values on the y-axis represent a given SNP’s absolute influence on the model’s raw output score (logit) for T1D (. While major patterns can be identified in the single runs, such as high activation in the HLA region, there is some variance in which SNPs are highly activated in other parts of the genome, where linkage disequilibrium (LD) and stochasticity during model training can contribute to the variance. By averaging the activations across single runs (aggregated), a clearer picture of which SNPs play the most important part can emerge.

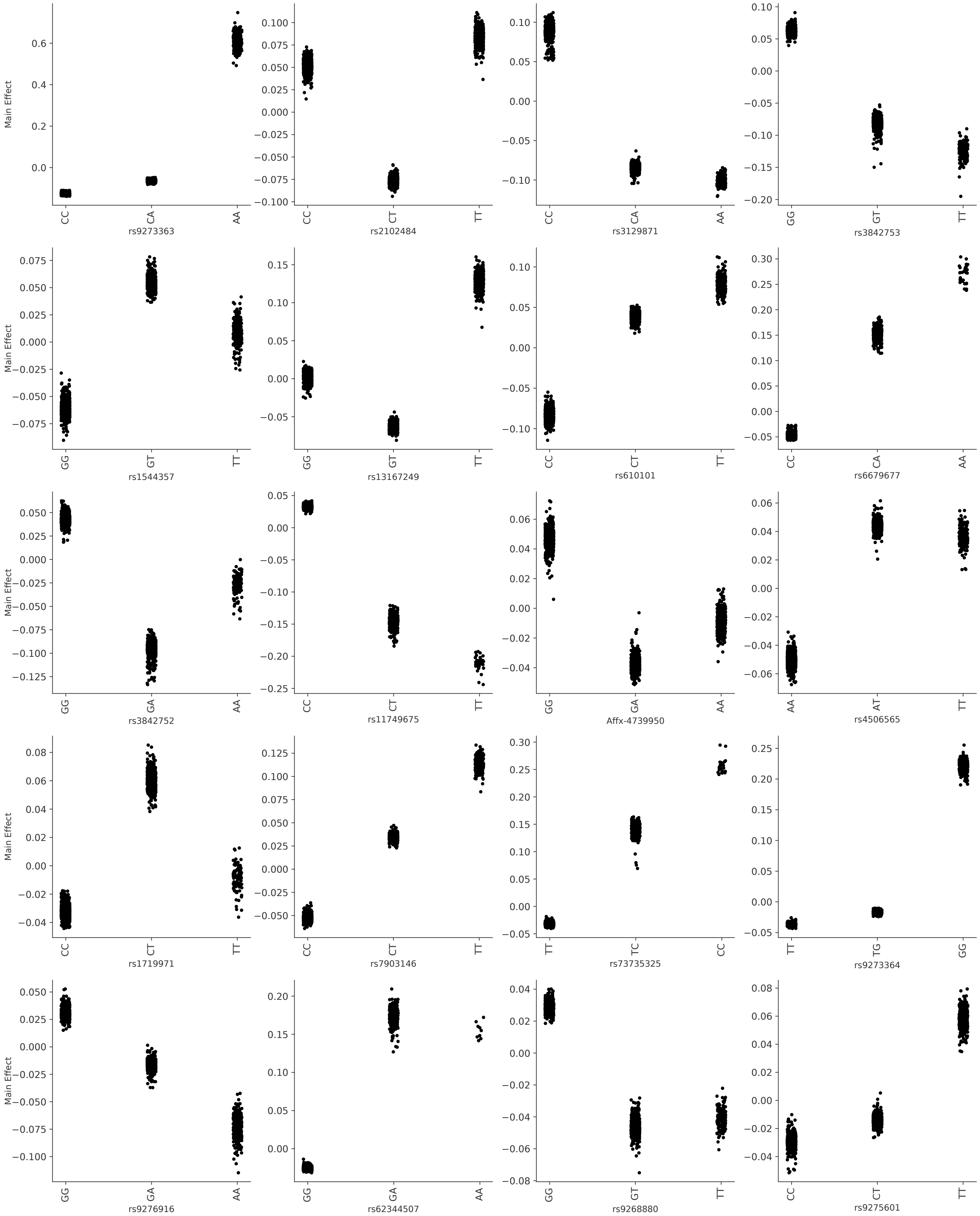

Supplementary Figure 11. Main effects of the top 20 most important identified from multiple GLN training runs for type 1 diabetes. Ten training runs with different seeds were performed with the GLN model, and the top 200 SNPs across all runs (on the validation set) used as candidates for the analysis. A gradient boosted decision trees model was trained on the top SNPs and analyzed for both main and interaction effects. The SNPs show both additive effects also non-additive effects where the heterozygote and homozygote show opposite effects.

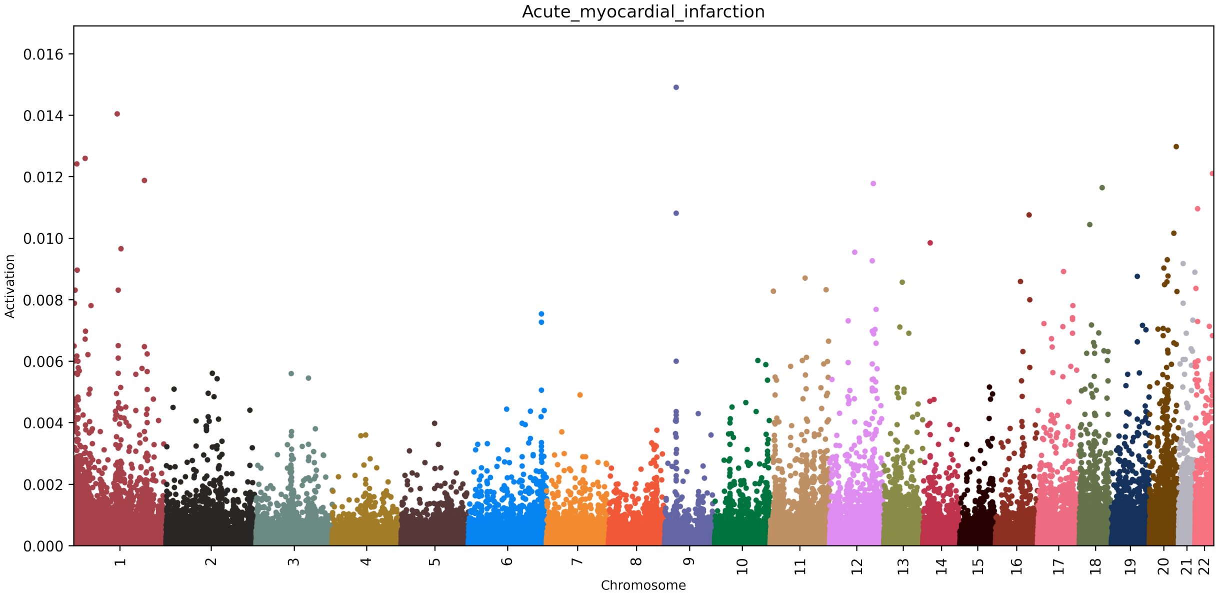

Supplementary Figure 12. SNP feature importance distribution using the GLN model for acute myocardial infarction. The values on the y-axis represent a given SNP’s absolute influence on the model’s raw output score (logit) for asthma.

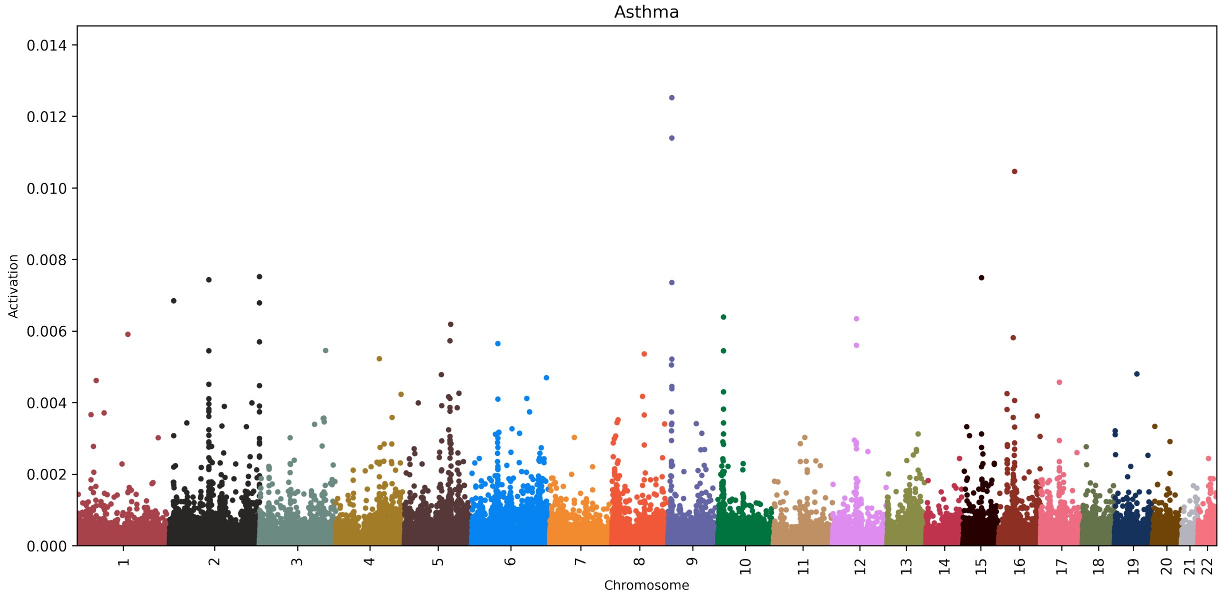

Supplementary Figure 13. SNP feature importance distribution using the GLN model for asthma. The values on the y-axis represent a given SNP's absolute influence on the model's raw output score (logit) for asthma.

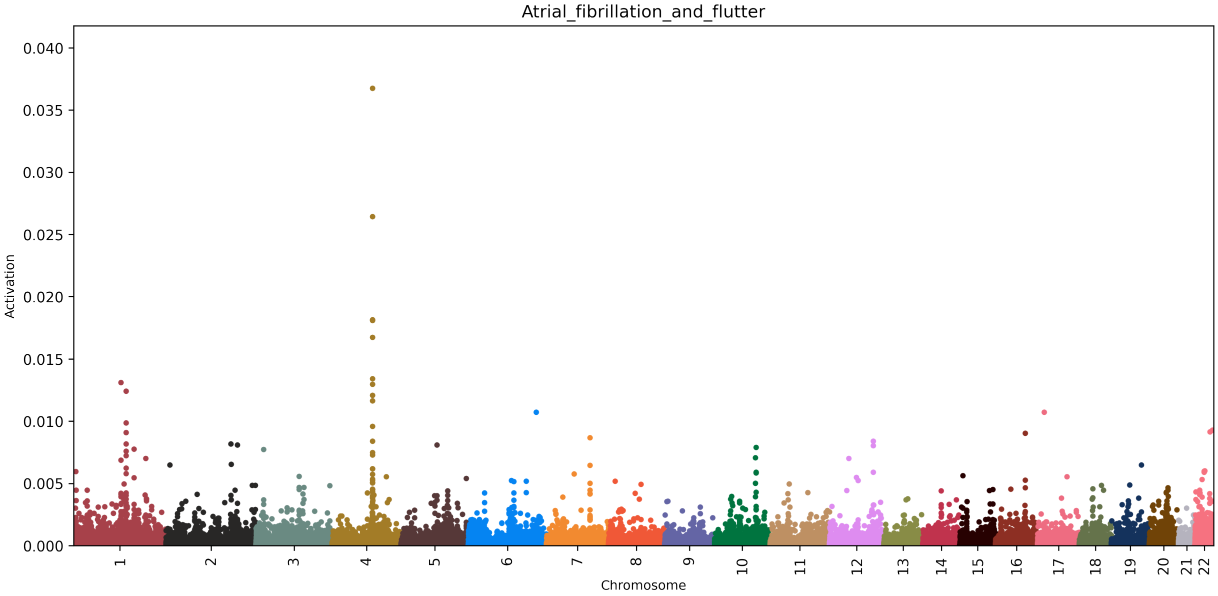

Supplementary Figure 14. SNP feature importance distribution using the GLN model for atrial fibrillation and flutter. The values on the y-axis represent a given SNP's absolute influence on the model's raw output score (logit) for atrial fibrillation and flutter.

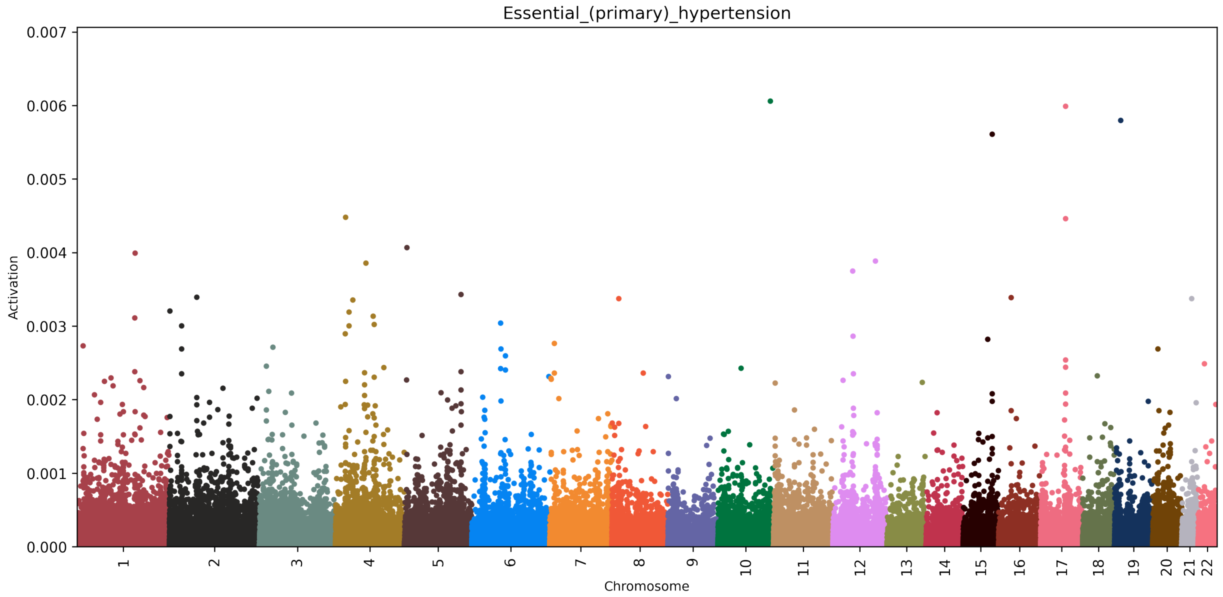

Supplementary Figure 15. SNP feature importance distribution using the GLN model for hypertension. The values on the y-axis represent a given SNP's absolute influence on the model's raw output score (logit) for hypertension.

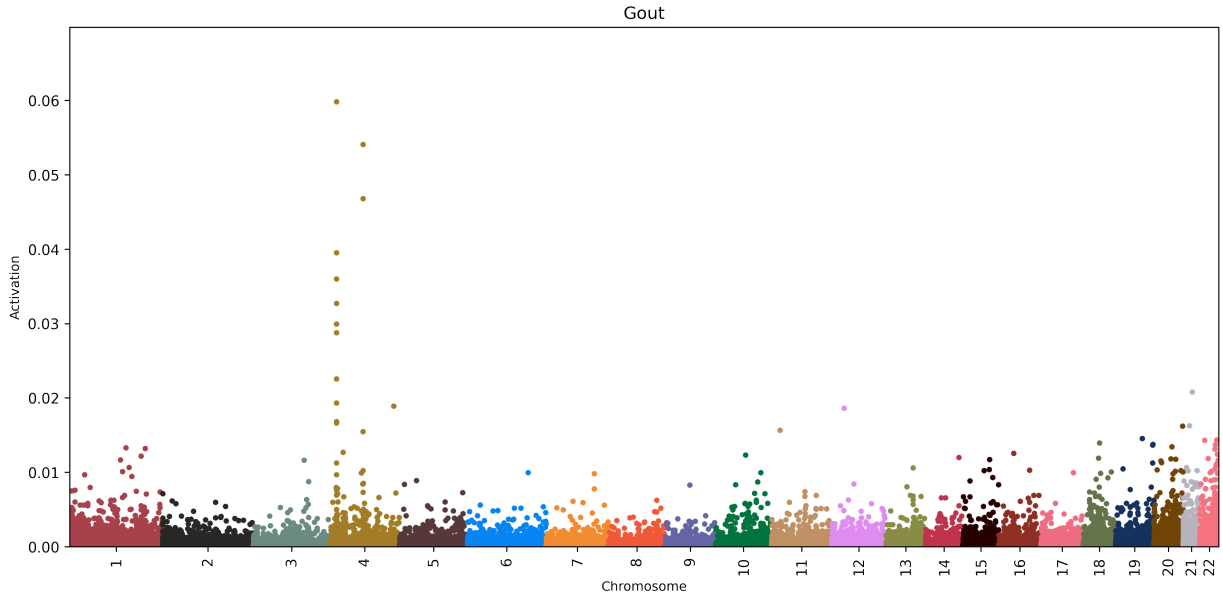

Supplementary Figure 16. SNP feature importance distribution using the GLN model for gout. The values on the y-axis represent a given SNP's absolute influence on the model's raw output score (logit) for gout.

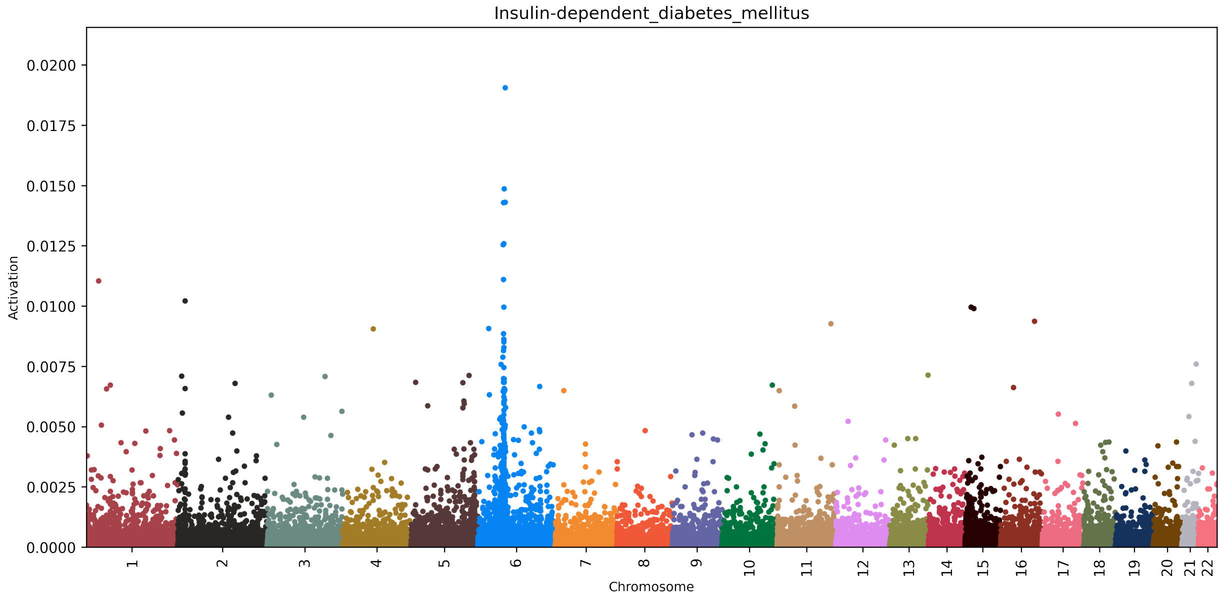

Supplementary Figure 17. SNP feature importance distribution using the GLN model for type 1 diabetes. The values on the y-axis represent a given SNP's absolute influence on the model's raw output score (logit) for Type 1 Diabetes.

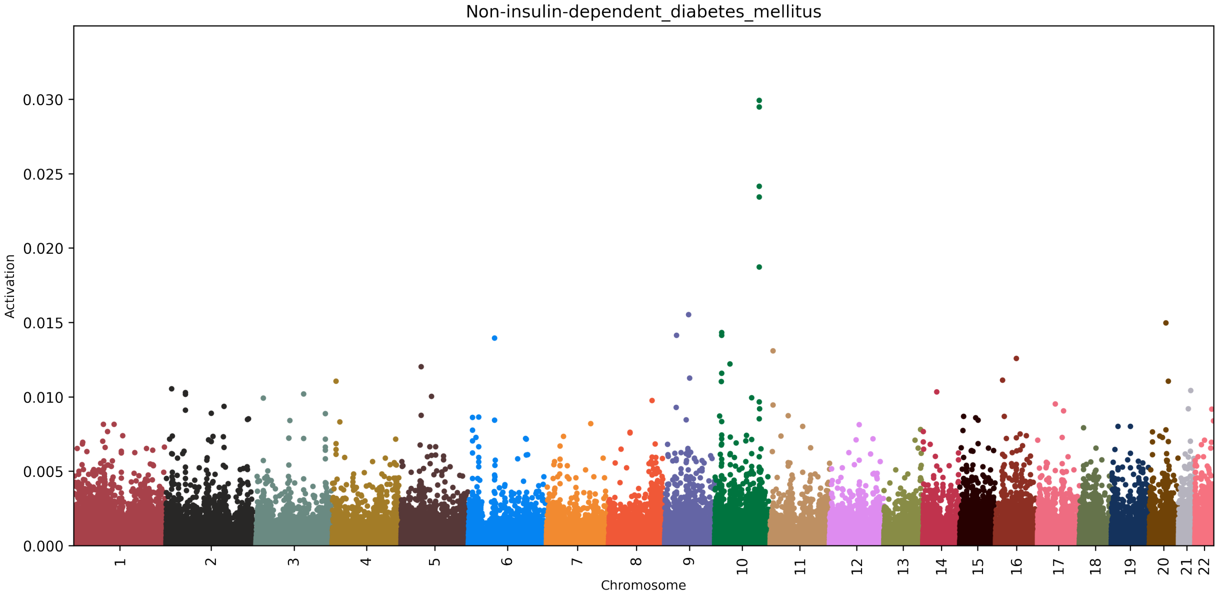

Supplementary Figure 18. SNP feature importance distribution using the GLN model for type 2 diabetes. The values on the y-axis represent a given SNP's absolute influence on the model's raw output score (logit) for Type 2 Diabetes.

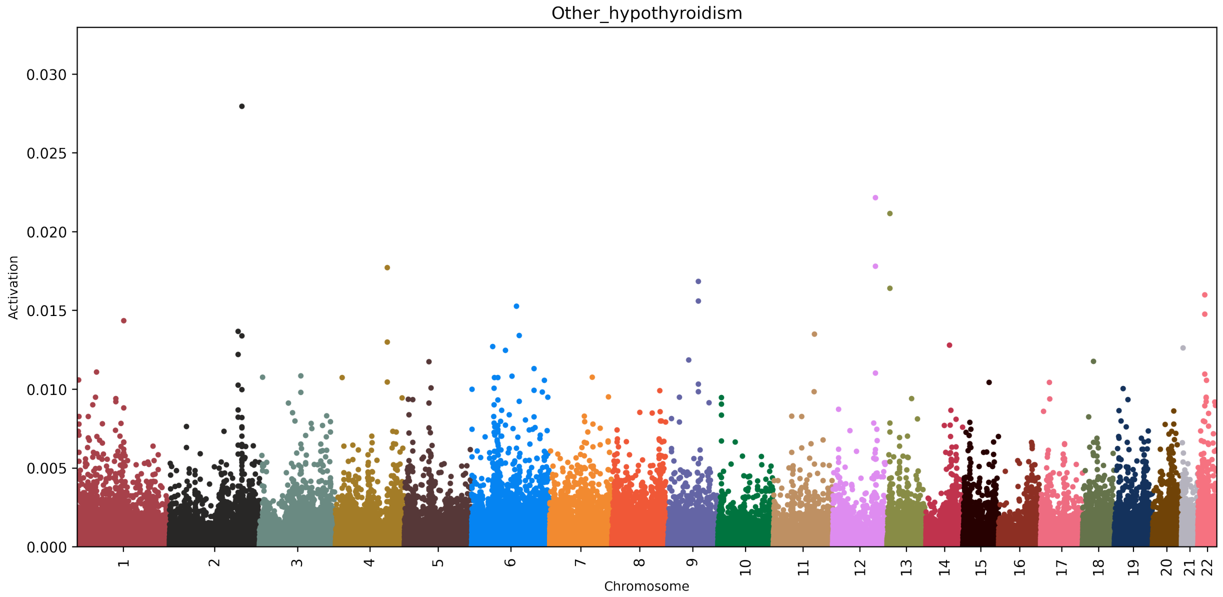

Supplementary Figure 19. SNP feature importance distribution using the GLN model for hypothyroidism. The values on the y-axis represent a given SNP's absolute influence on the model's raw output score (logit) for hypothyroidism.

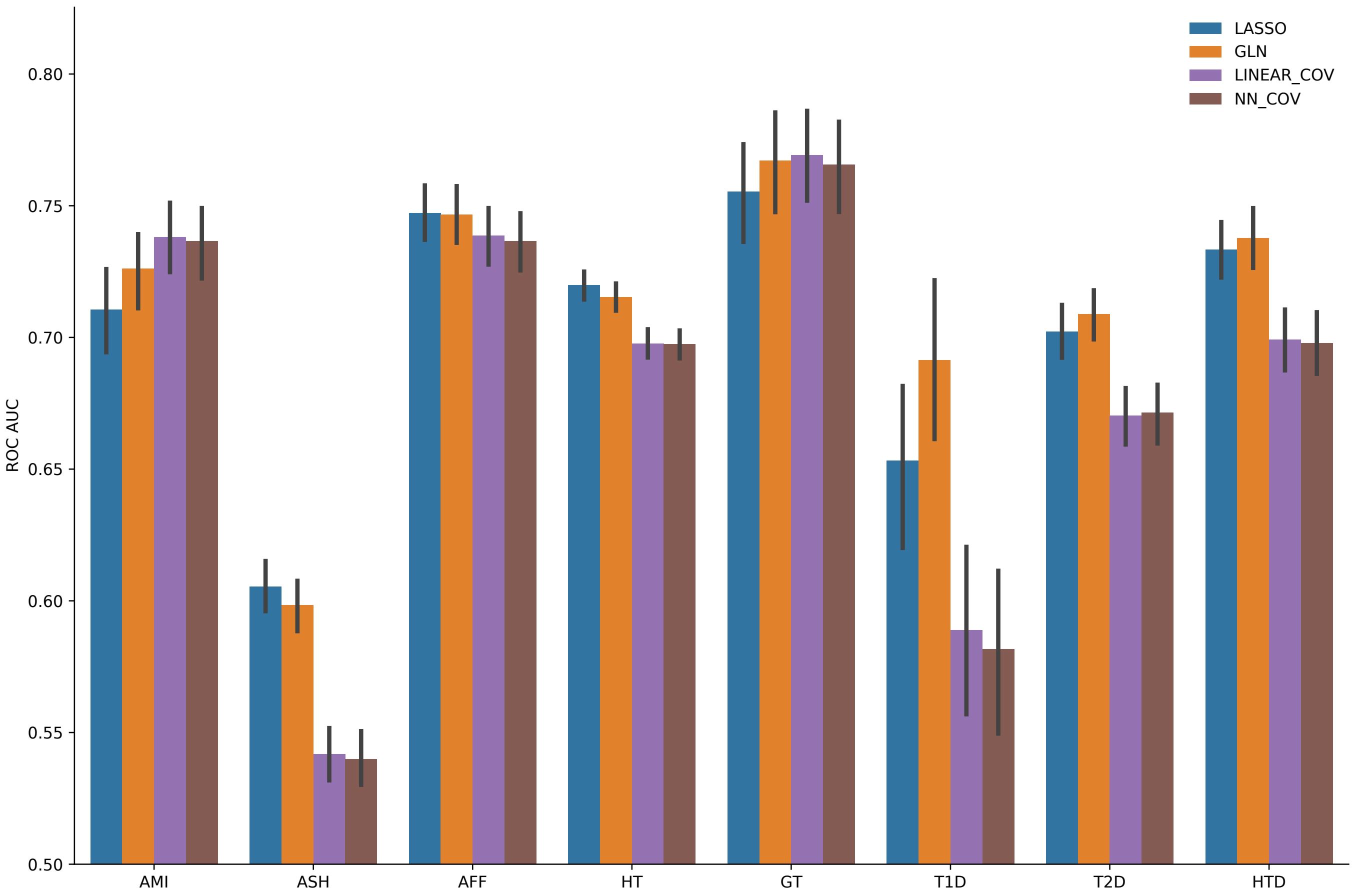

Supplementary Figure 20. Comparison of using the LASSO (blue) and GLN (orange) models with the covariates age, sex, and first 10 PCs and genotype against only using the covariates modelled with a linear (purple) and neural network-based model (brown), measured in ROC-AUC for the 8 benchmark traits. When only using the covariates, the same model architecture is used but with the genotype modality omitted. Bars represent the 95% CI from 1,000 bootstrap replicates on the held-out test set. **AMI:** Acute myocardial infarction, **ASH:** Asthma, **AFF:** Atrial fibrillation and flutter, **HT**: Hypertension, **GT:** Gout, **T1D:** Type 1 diabetes, **T2D:** Type 2 diabetes, **HTD:** Hypothyroidism.

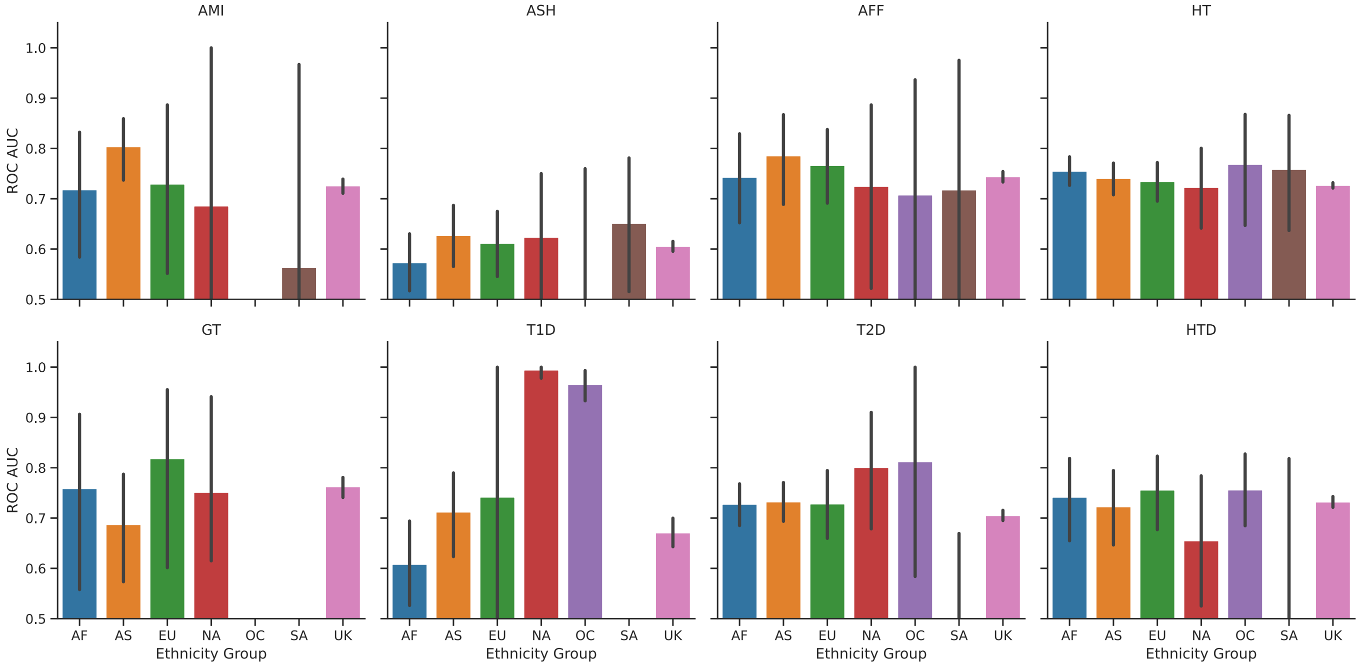

Supplementary Figure 21. Comparison of GLN performance on the held-out test set across 8 traits reported in ROC AUC, stratified by continent of origin groups Africa (AF, blue), Asia (AS, orange), Europe (EU, green), North-America (NA, red), Oceania (OC, purple), South-America (SA, brown). For AMI, no test-set cases were in the OC group. For GT, no test-set cases were present for OC and SA. Therefore, no bars were drawn for those combinations. The GLN training was done by using 5-fold Monte Carlo cross validation with the same model configuration for each fold. To get the final GLN results, an ensemble across all folds was performed. All models were adjusted for age, sex, and the first 10 genomic principal components (PCs). Bars represent the 95% CI from 1,000 bootstrap replicates on the held-out test set. **AMI:** Acute myocardial infarction, **ASH:** Asthma, **AFF:** Atrial fibrillation and flutter, **HT**: Hypertension, **GT:** Gout, **T1D:** Type 1 diabetes, **T2D:** Type 2 diabetes, **HTD:** Hypothyroidism.

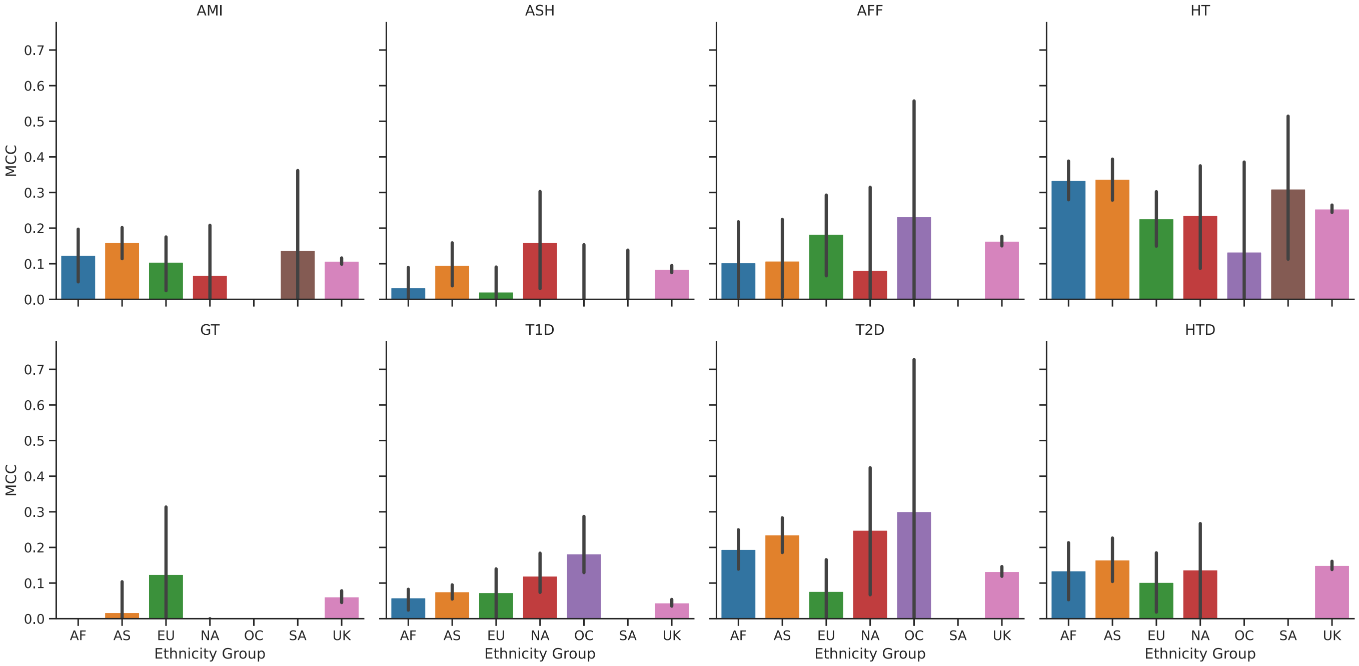

Supplementary Figure 22. Comparison of GLN performance on the held-out test set across 8 traits reported in ROC AUC, stratified by continent of origin groups Africa (AF, blue), Asia (AS, orange), Europe (EU, green), North-America (NA, red), Oceania (OC, purple), South-America (SA, brown). For AMI, no test-set cases were in the OC group. For GT, no test-set cases were present for OC and SA. Therefore, no bars were drawn for those combinations. The GLN training was done by using 5-fold Monte Carlo cross validation with the same model configuration for each fold. To get the final GLN results, an ensemble across all folds was performed. All models were adjusted for age, sex, and the first 10 genomic principal components (PCs). Bars represent the 95% CI from 1,000 bootstrap replicates on the held-out test set. No cases **AMI:** Acute myocardial infarction, **ASH:** Asthma, **AFF:** Atrial fibrillation and flutter, **HT**: Hypertension, **GT:** Gout, **T1D:** Type 1 diabetes, **T2D:** Type 2 diabetes, **HTD:** Hypothyroidism.

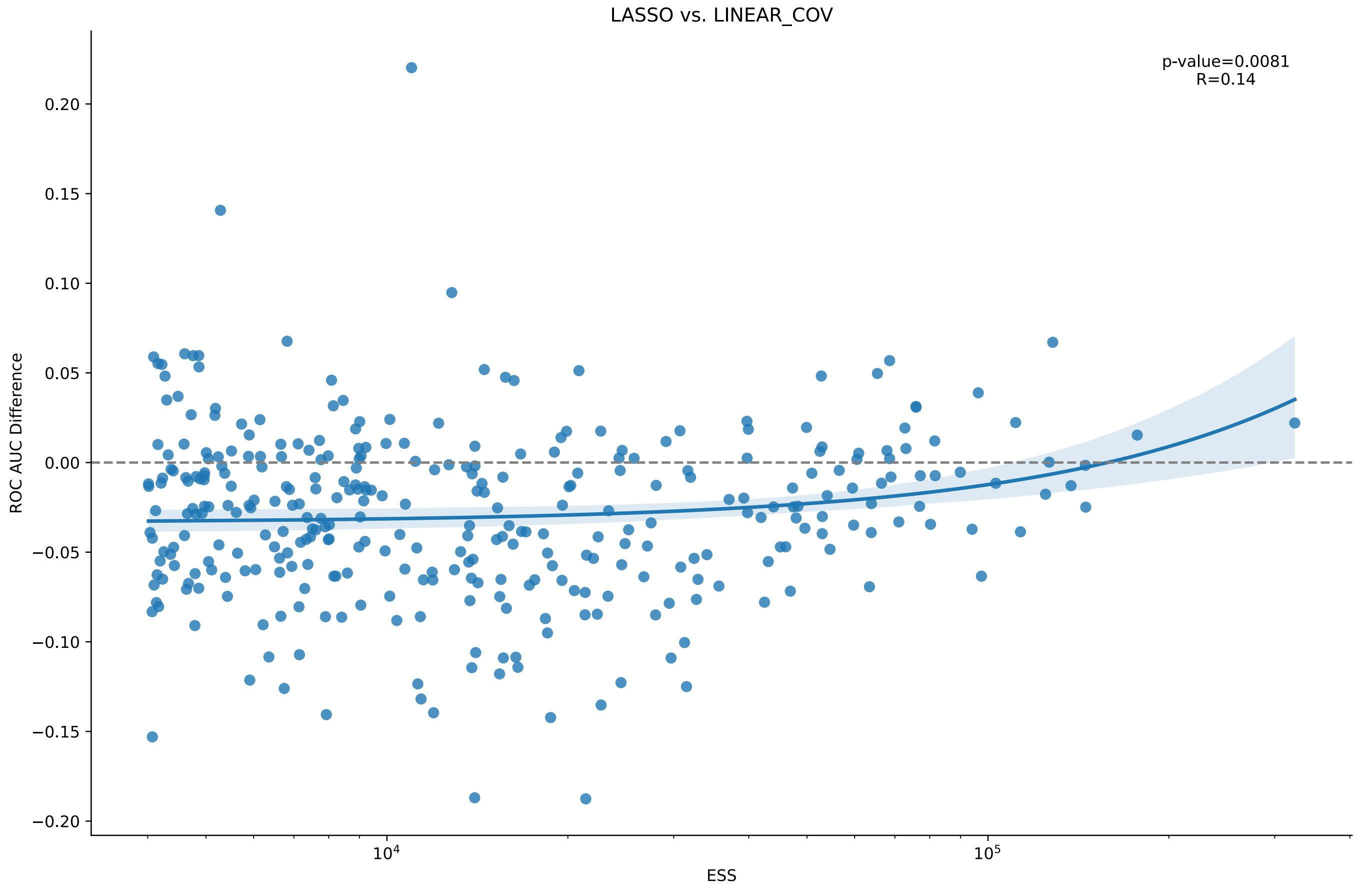

Supplementary Figure 23. Difference in performance of LASSO model versus linear covariate based model (using sex, age, and the first 10 genotype principal components) as a function of effective sample size (ESS). A positive difference indicates that the LASSO model performed better on the test set compared to the linear covariate based model.

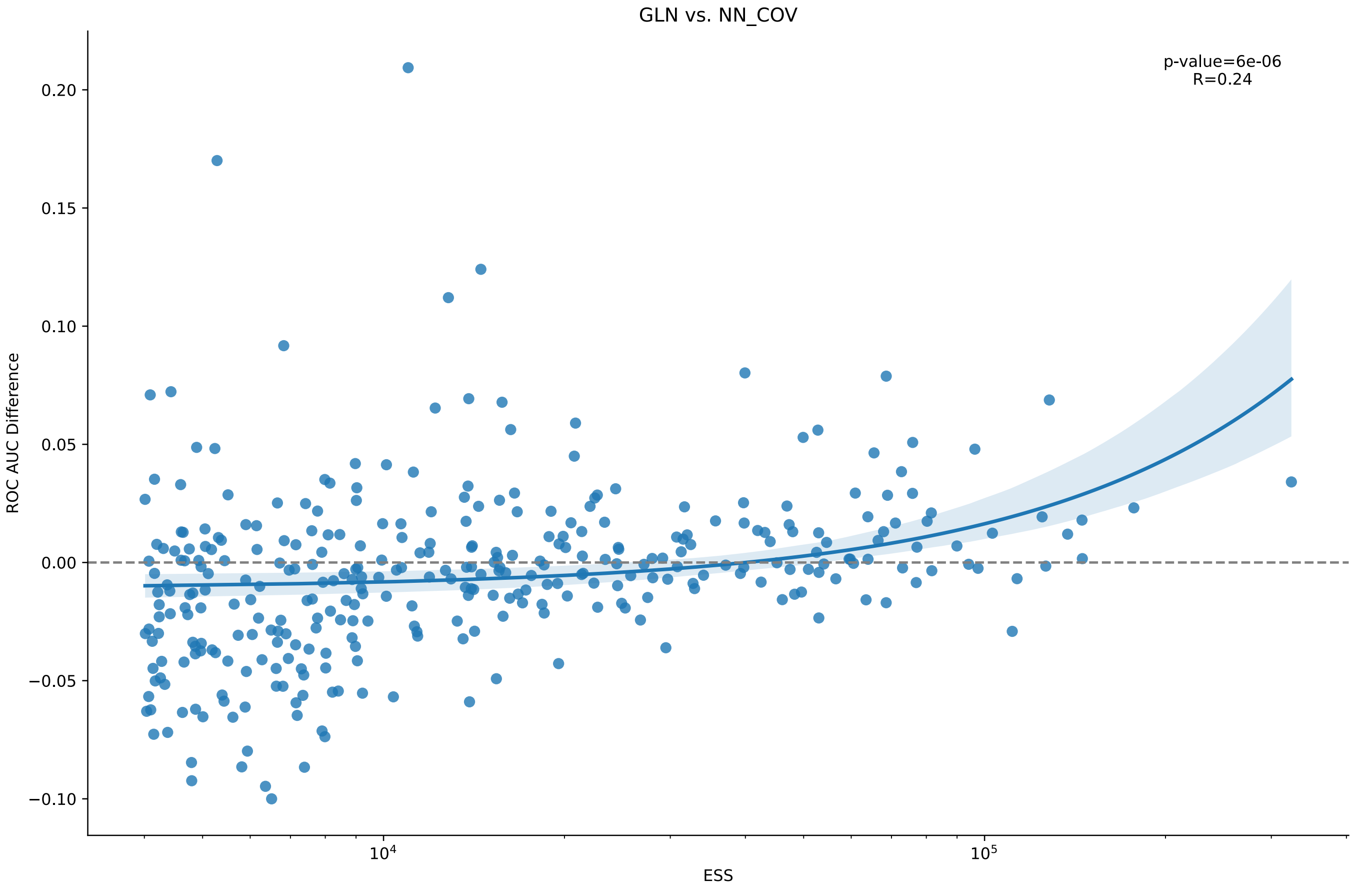

Supplementary Figure 24. Difference in performance of GLN model versus a neural network (NN) covariate based model (using sex, age, and the first 10 genotype principal components) as a function of effective sample size (ESS). A positive difference indicates that the GLN model performed better on the test set compared to the NN covariate based model.

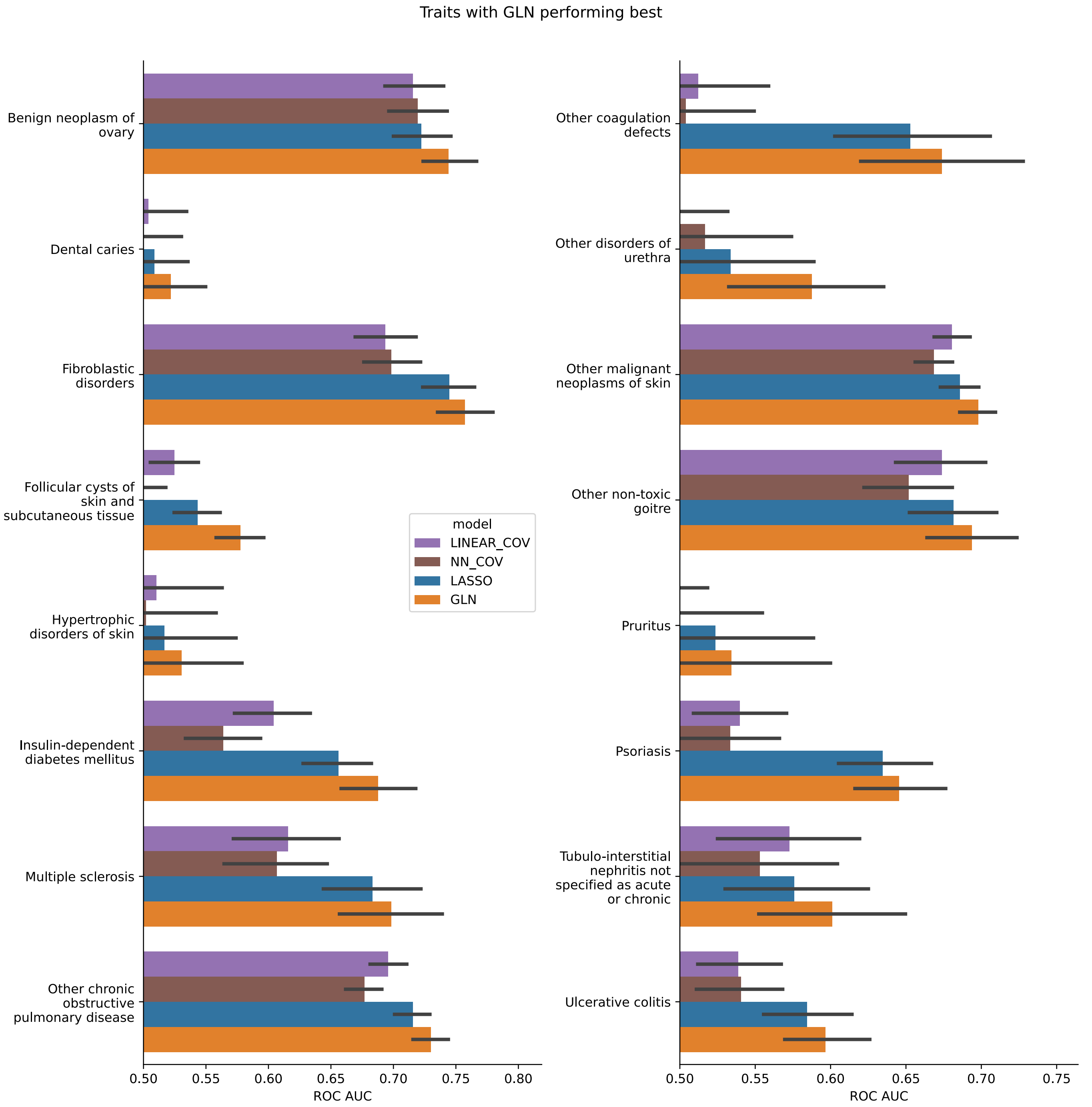

Supplementary Figure 25. Traits in the large-scale training where the GLN (orange) model showed the best performance (with a ROC-AUC difference greater than 0.01) and both GLN and LASSO (blue) had a better performance than either of the linear (purple) or neural network (brown) base covariates models on the held-out test set. All models were adjusted for sex, age, and the first 10 principal components. Bars represent the 95% CI from 1,000 bootstrap replicates on the held-out test set.

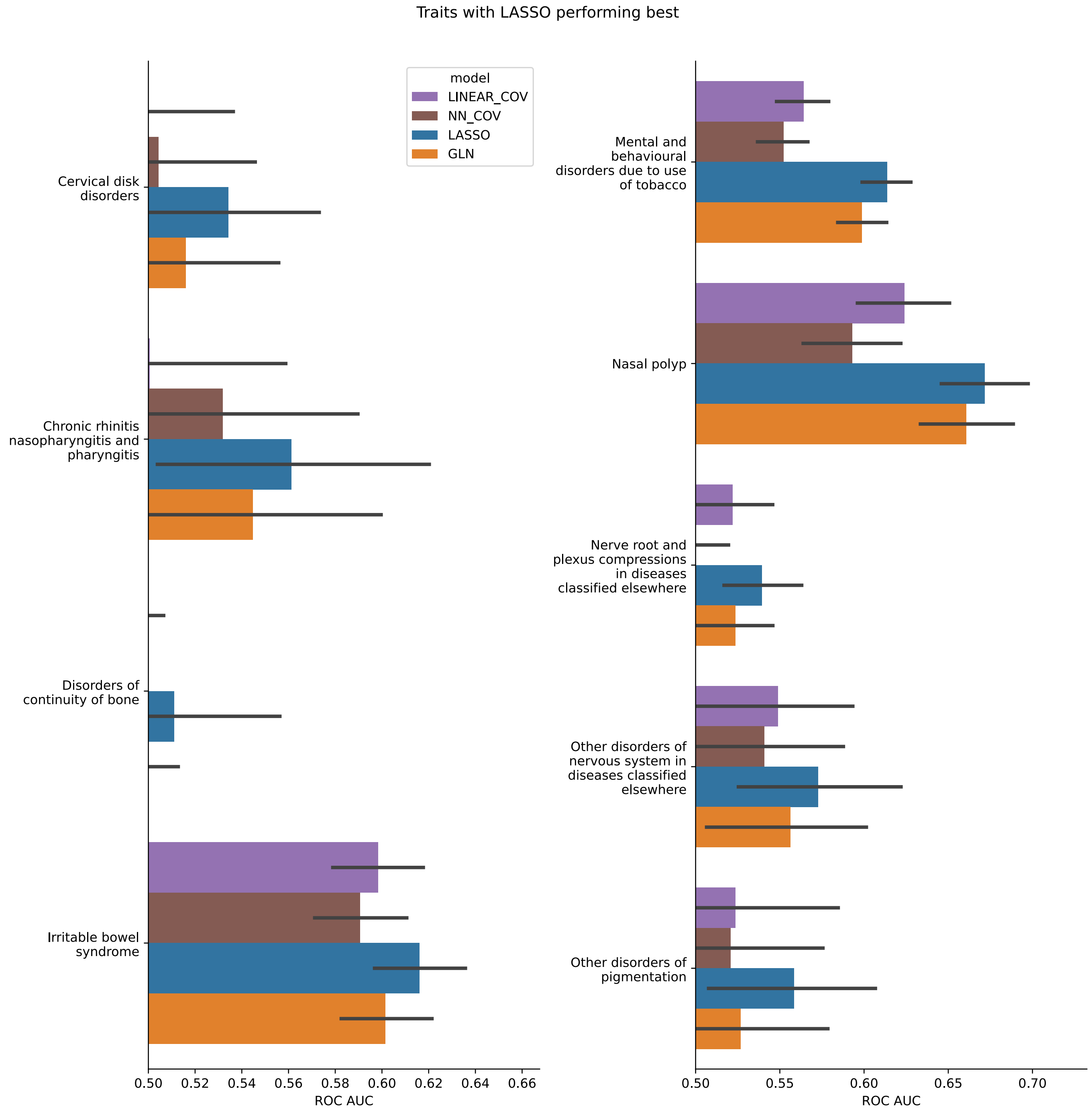

Supplementary Figure 26. Traits in the large-scale training where the LASSO (blue) model showed the best performance (with a ROC-AUC difference greater than 0.01) and both GLN (orange) and LASSO had a better performance than either of the linear (purple) or neural network (brown) base covariates models on the test set. All models were adjusted for sex, age, and the first 10 principal components. Bars represent the 95% CI from 1,000 bootstrap replicates on the held-out test set.

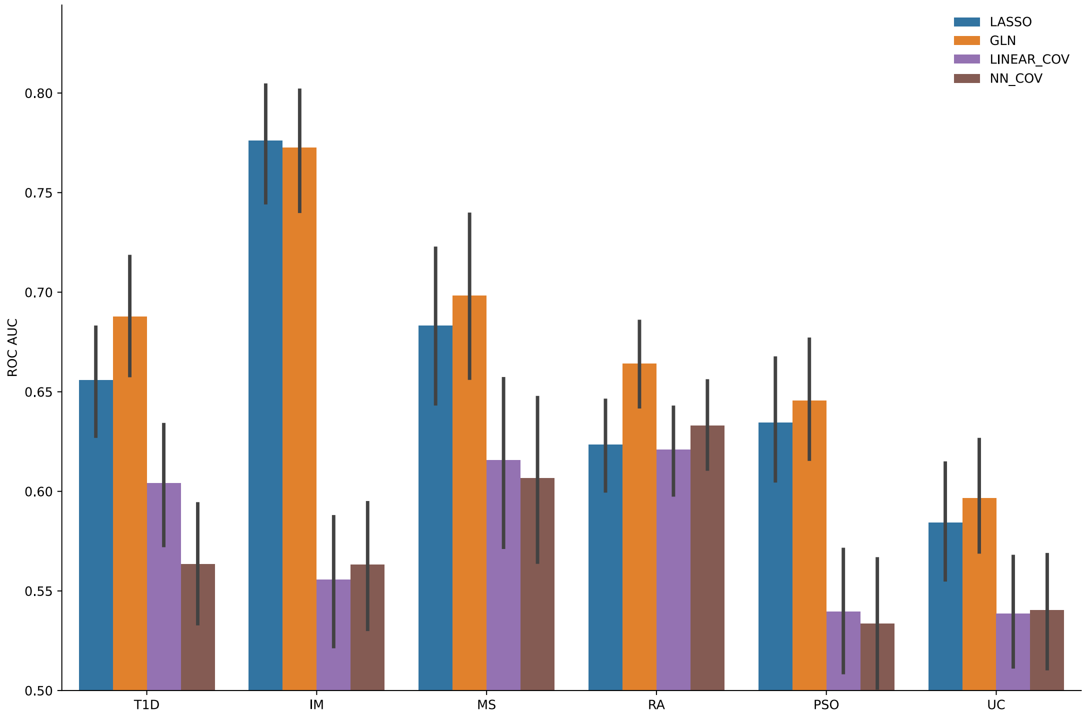

Supplementary Figure 27. Comparison of using the LASSO (blue) and GLN (orange) models with the covariates age, sex, and first 10 PCs and genotype against only using the covariates modelled with a linear (purple) and neural network-based model (brown), for autoimmune traits previously researched for interaction effects. When only using the covariates, the same model architecture is used but with the genotype modality omitted. Performance is measured in ROC-AUC on the held-out test set. Bars represent the 95% CI from 1,000 bootstrap replicates on the held-out test set. **T1D:** Type 1 Diabetes, **IM:** Intestinal Malabsortion, **MS:** Multiple Sclerosis, **RA**: Rheumatoid Arthritis, **PSO:** Psoriasis, **UC:** Ulcerative Colitis.

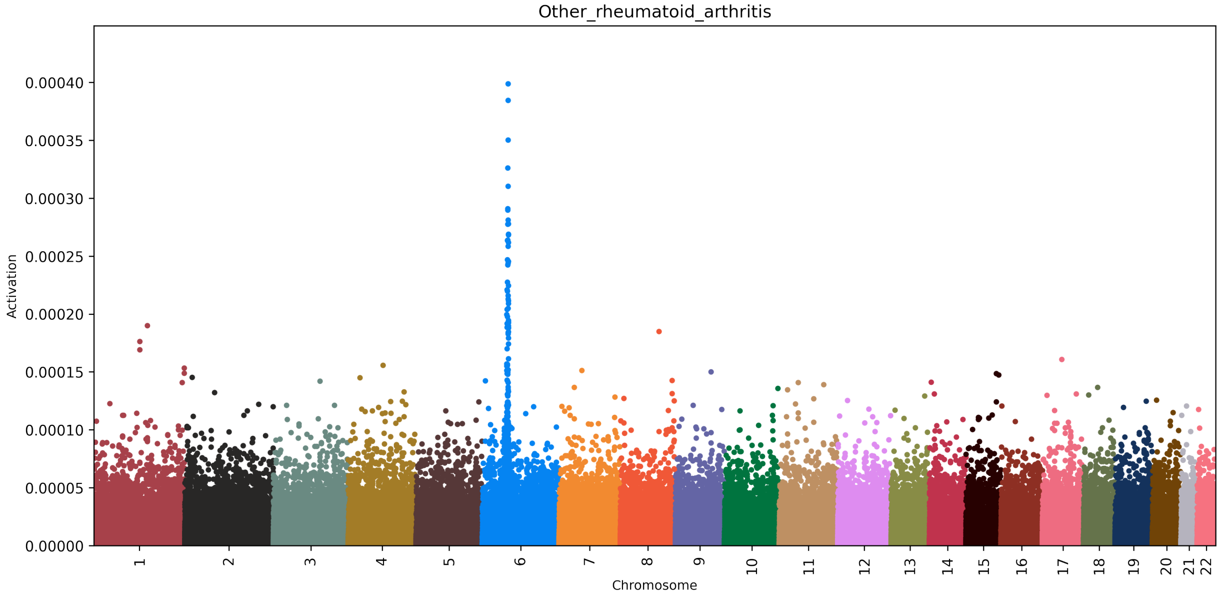

Supplementary Figure 28. SNP feature importance distribution using the LASSO model for rheumatoid arthritis. The values on the y-axis represent a given SNP’s absolute influence on the model’s raw output score (logit) for rheumatoid arthritis.

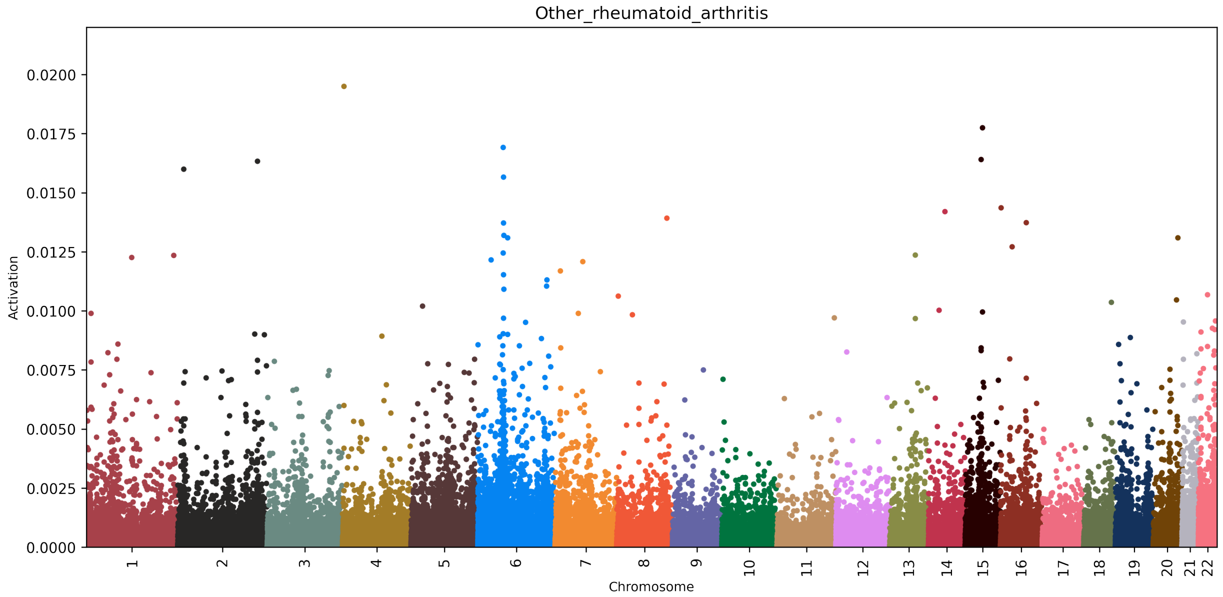

Supplementary Figure 29. SNP feature importance distribution using the GLN model for rheumatoid arthritis. The values on the y-axis represent a given SNP's absolute influence on the model's raw output score (logit) for rheumatoid arthritis.

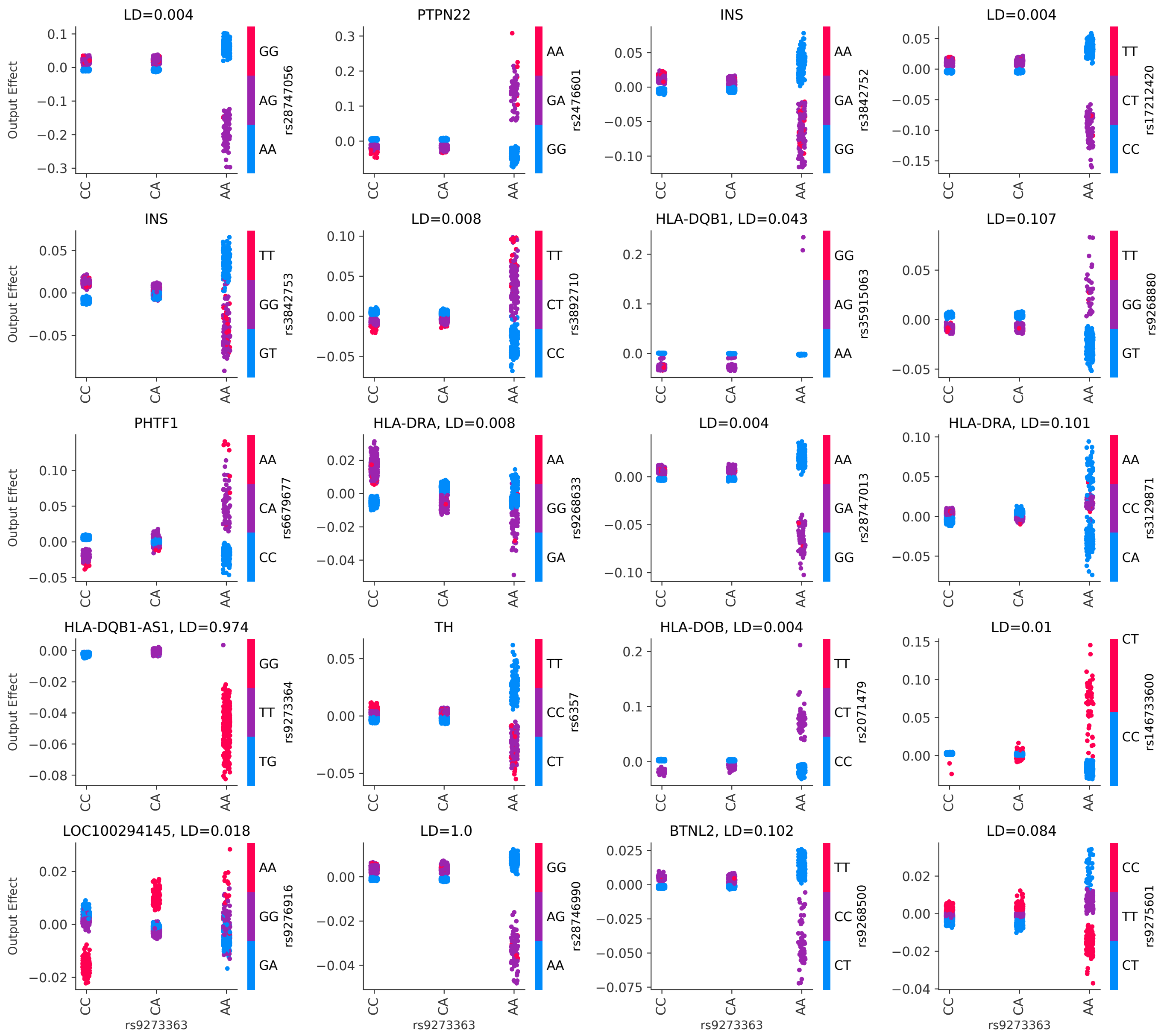

Supplementary Figure 30. Interaction effects between rs9273363 and the other top 20 most important SNPs (according to interaction effects, excluding main effects) identified when training the gradient boosted decision trees on the top 200 SNPs from the GLN model for T1D. The x-axis corresponds to the rs9273363 alleles, while the y-axis corresponds to the effect of the interaction on the raw gradient boosted decision trees (GBDT) model output (logit). Each dot in the figure represents one sample, and the dot color indicates the allele of the SNP interacting with rs9273363. If the SNP interacting with rs9273363 was mapped to a gene, it is shown above the relevant sub-figure. Additionally, if the interacting SNP resides on chr6 as rs9273363 does, the linkage disequilibrium (LD) value was computed (in R^2) based on the British in England and Scotland population of LDLink (95) is shown.

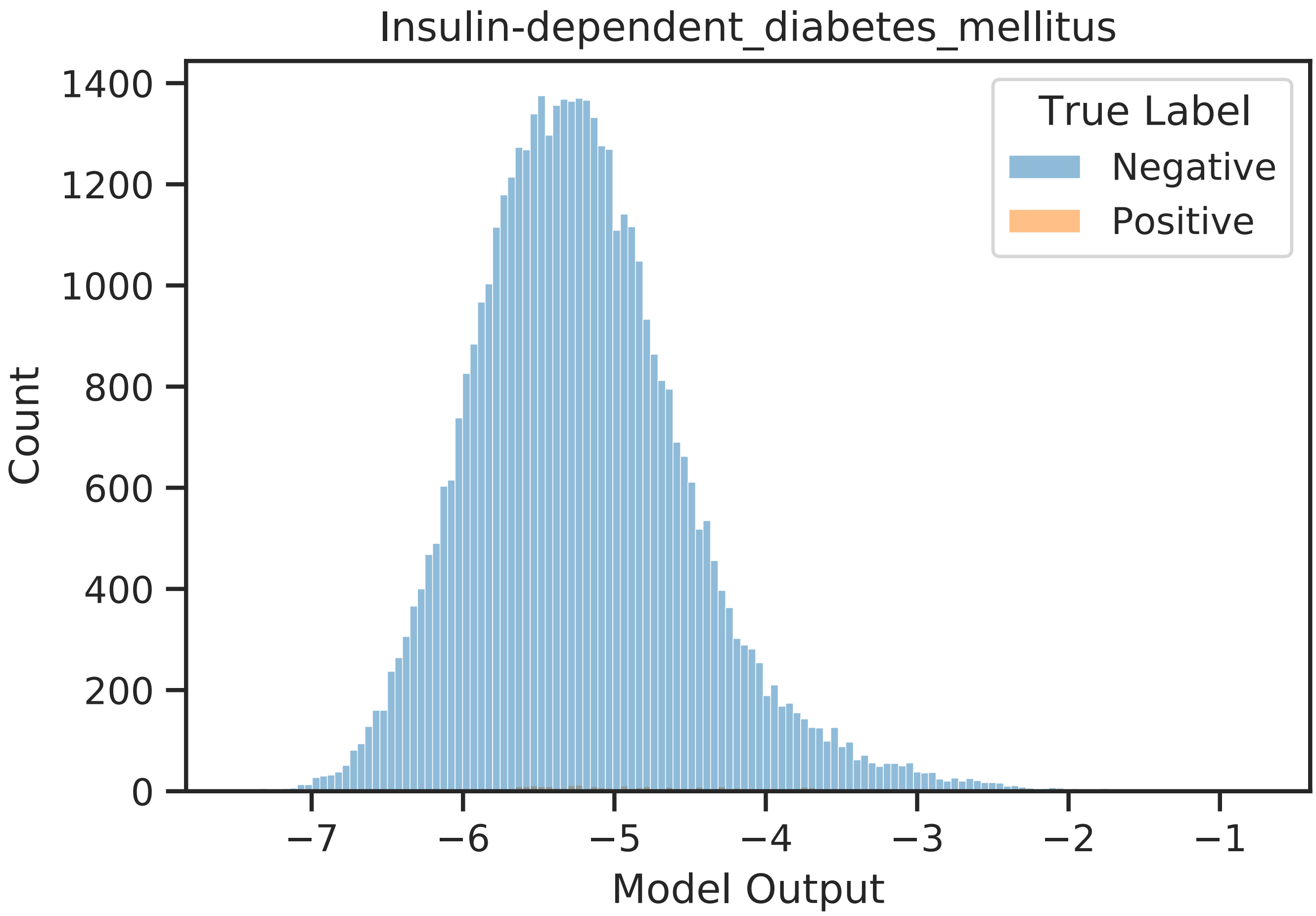

Supplementary Figure 31. Output distribution of the gradient boosted decision trees (GBDT) model trained on the top 200 GLN important SNPs, where the top 200 SNPs are chosen from the average feature importance assigned across 10 GLN training runs with different seeds each.

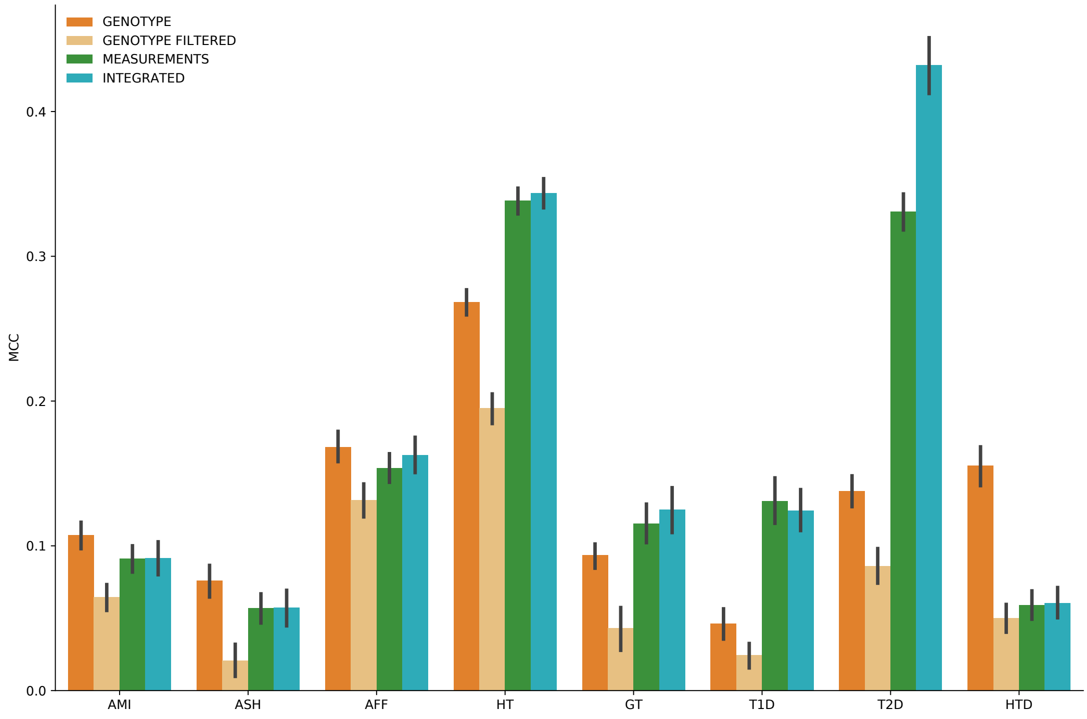

Supplementary Figure 32. Comparison of model performance using Genotype (orange), Genotype Filtered (light orange), Measurement (green) and Integrated (teal) data in MCC on the held-out test set. Bars represent the 95% CI from 1,000 bootstrap replicates on the held-out test set. **AMI:** Acute myocardial infarction, **ASH:** Asthma, **AFF:** Atrial fibrillation and flutter, **HT**: Hypertension, **GT:** Gout, **T1D:** Type 1 diabetes, **T2D:** Type 2 diabetes, **HTD:** Hypothyroidism.

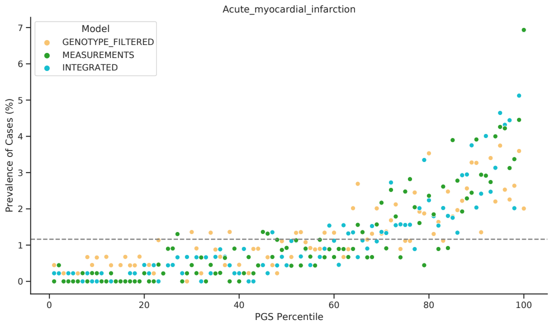

(a)

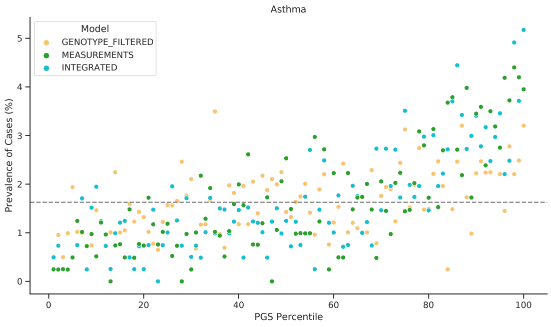

(b)

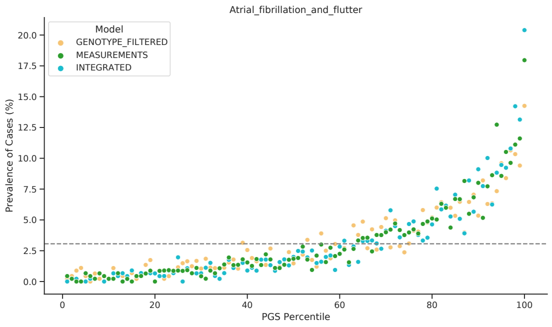

(c)

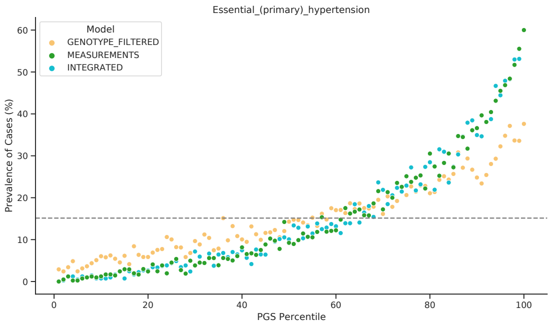

(d)

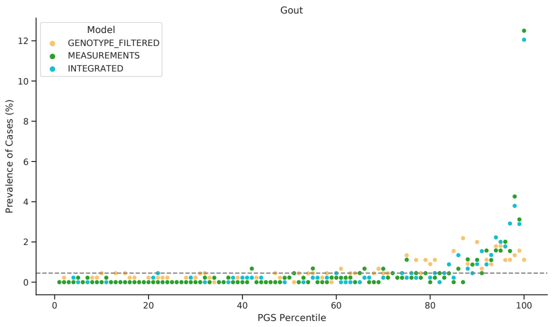

(e)

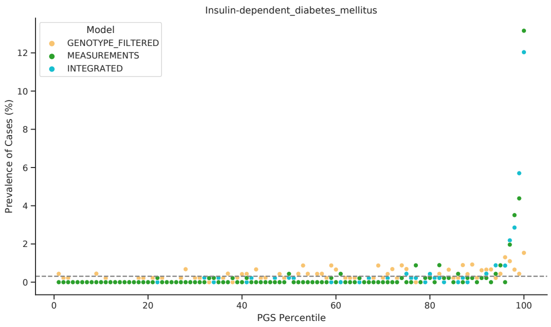

(f)

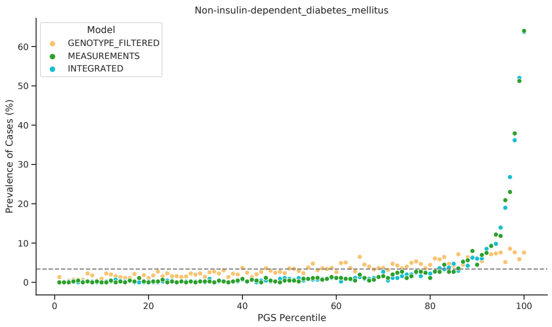

(g)

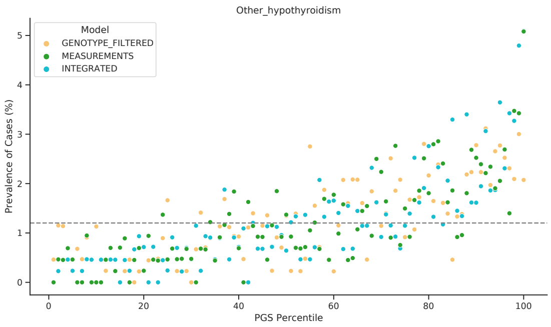

(h)

Supplementary Figure 33. Prevalence plots comparing models using Genotype Filtered (light orange), Measurement (green) and Integrated (teal) data, using the GLN model. The dashed line represents the average prevalence in the test set, after filtering for time of diagnosis.

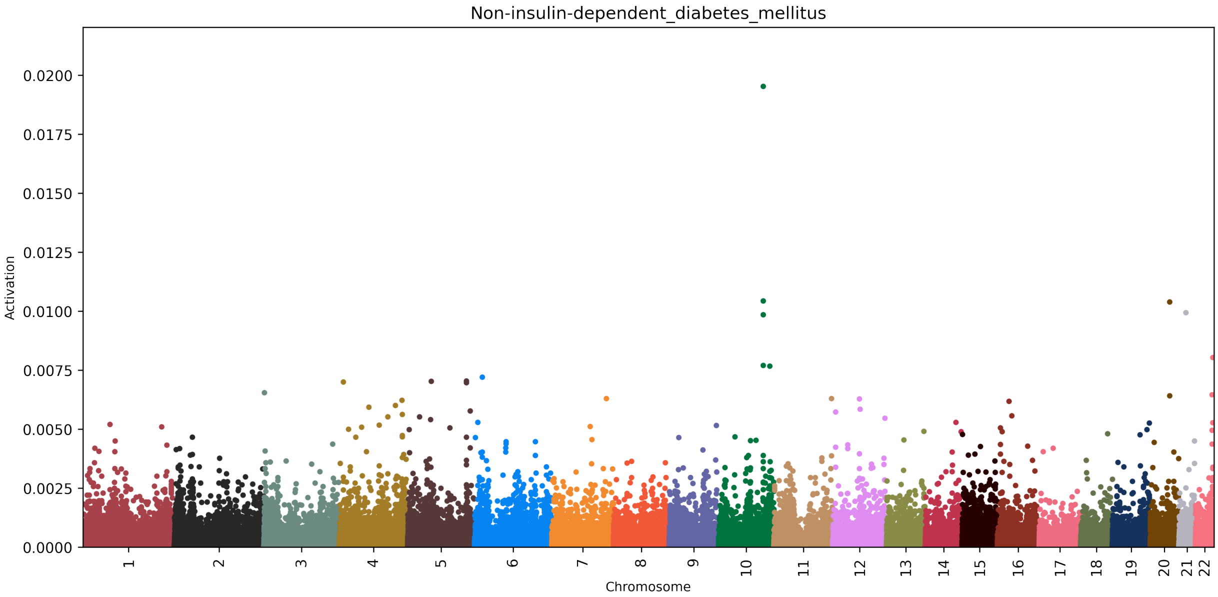

Supplementary Figure 34. SNP feature importance distribution using the GLN model for type 2 diabetes, when including clinical and biochemical measurements. The values on the y-axis represent a given SNP’s absolute influence on the model’s raw output score (logit) for type 2 diabetes.

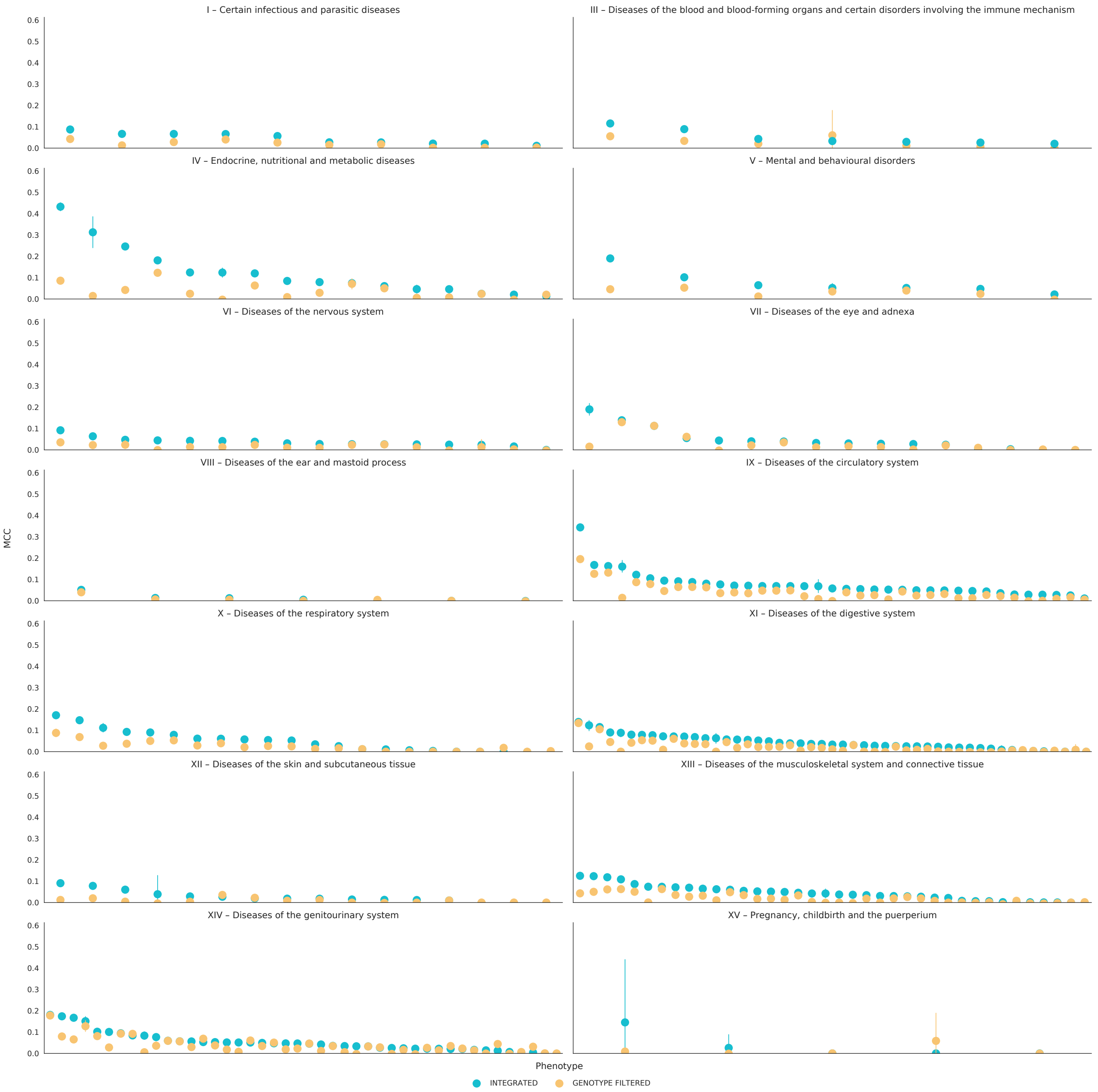

Supplementary Figure 35. Summary of MCC performance on the held-out test set across all the 290 traits that had a time measured column associated with them, with Integrated data (teal) compared with Genotype Filtered data (light orange), filtered for time of diagnosis. Bars represent the 95% CI from 1,000 bootstrap replicates on the held-out test set.

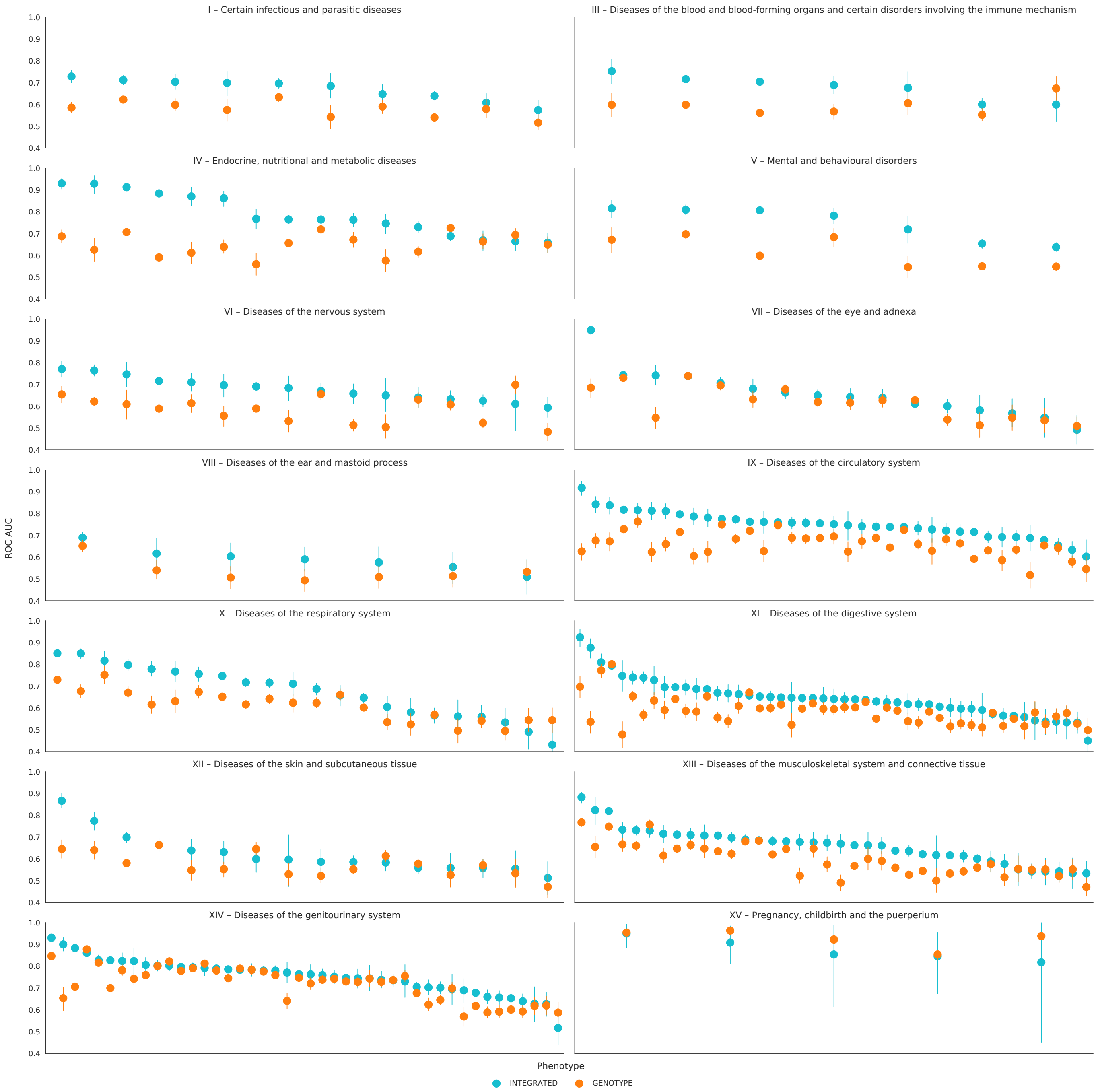

Supplementary Figure 36. Summary of ROC-AUC performance on the held-out test set across all the 290 traits that had a time measured column associated with them, with Integrated data (teal) compared with Genotype data (orange), filtered for time of diagnosis. Bars represent the 95% CI from 1,000 bootstrap replicates on the held-out test set.

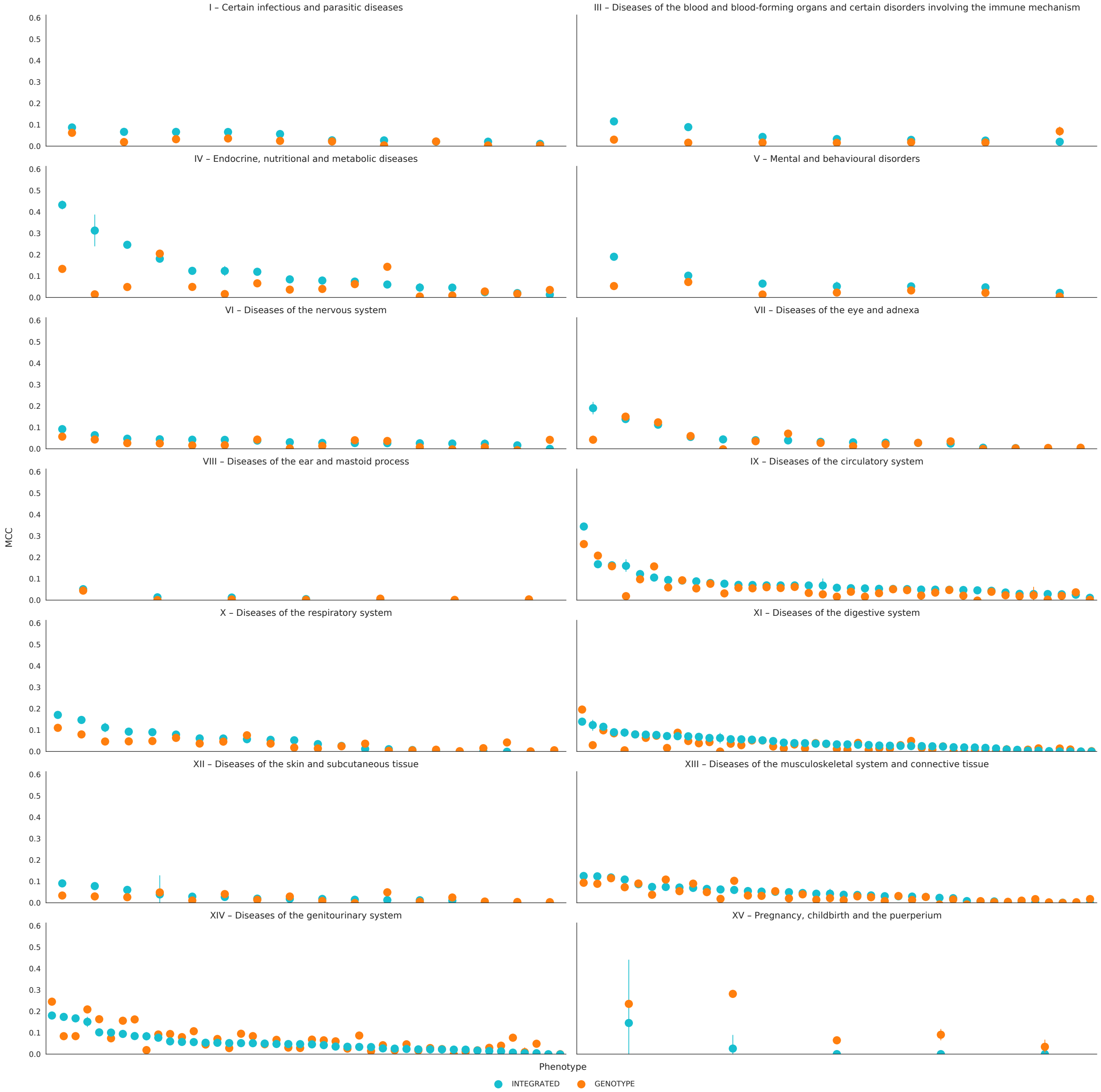

Supplementary Figure 37. Summary of MCC performance on the held-out test set across all the 290 traits that had a time measured column associated with them, with Integrated data (teal) compared with Genotype data (orange), filtered for time of diagnosis. Bars represent the 95% CI from 1,000 bootstrap replicates on the held-out test set.

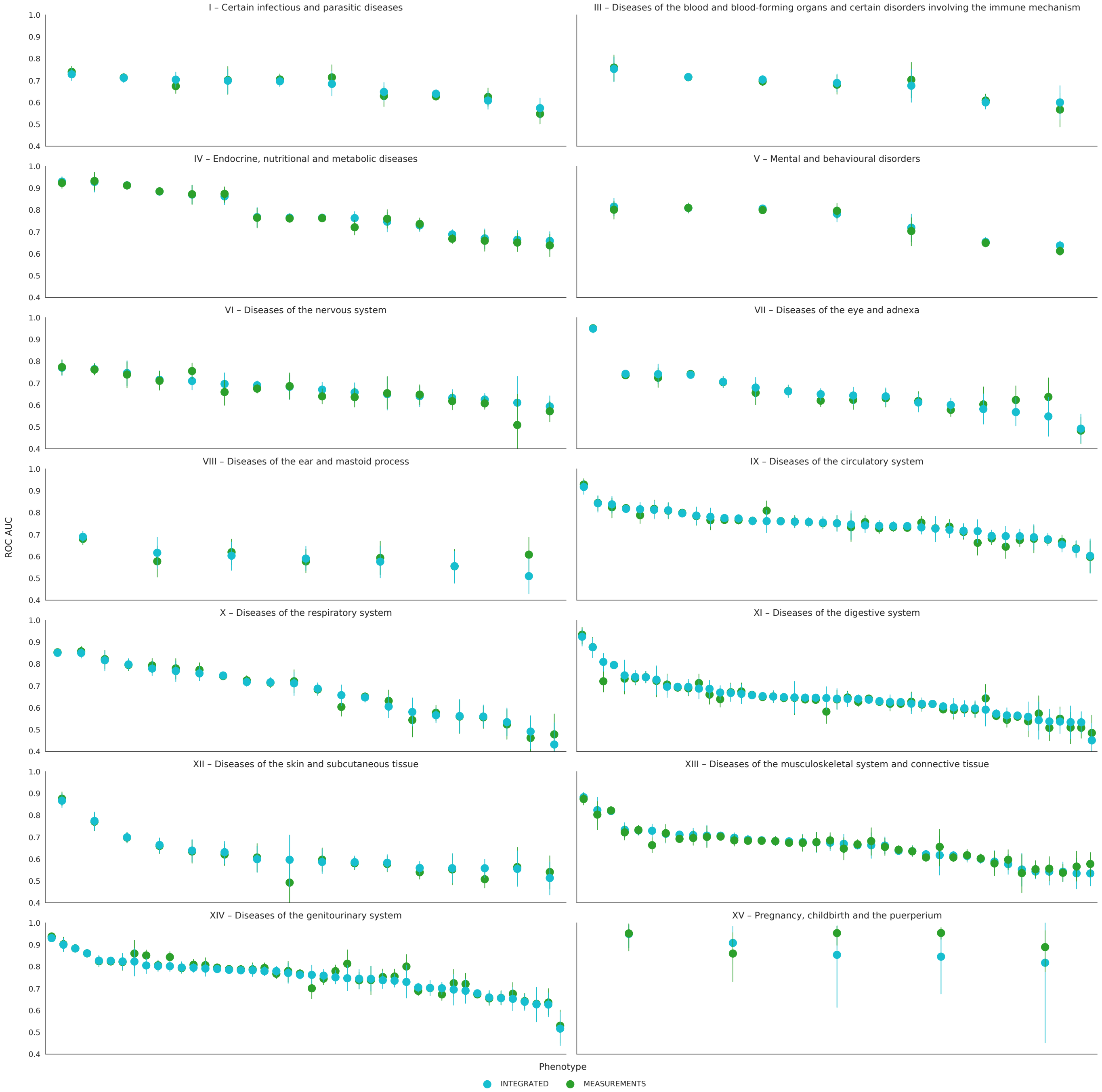

Supplementary Figure 38. Summary of ROC-AUC performance on the held-out test set across all the 290 traits that had a time measured column associated with them, with Integrated data (teal) compared with Measurement data (green), filtered for time of diagnosis. Bars represent the 95% CI from 1,000 bootstrap replicates on the held-out test set.

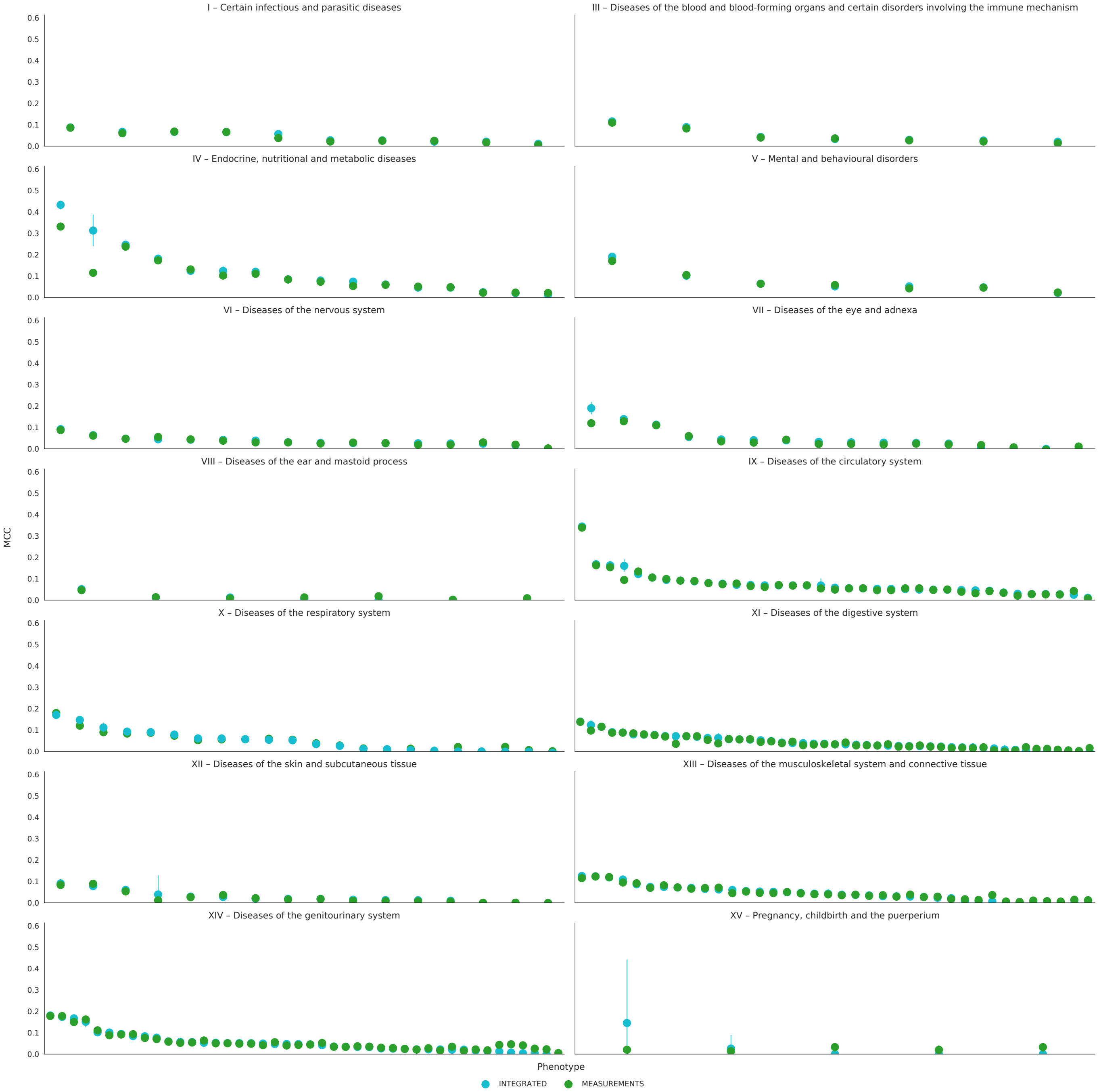

Supplementary Figure 39. Summary of MCC performance on the held-out test set across all the 290 traits that had a time measured column associated with them, with Integrated data (teal) compared with Measurement data (green), filtered for time of diagnosis. Bars represent the 95% CI from 1,000 bootstrap replicates on the held-out test set.

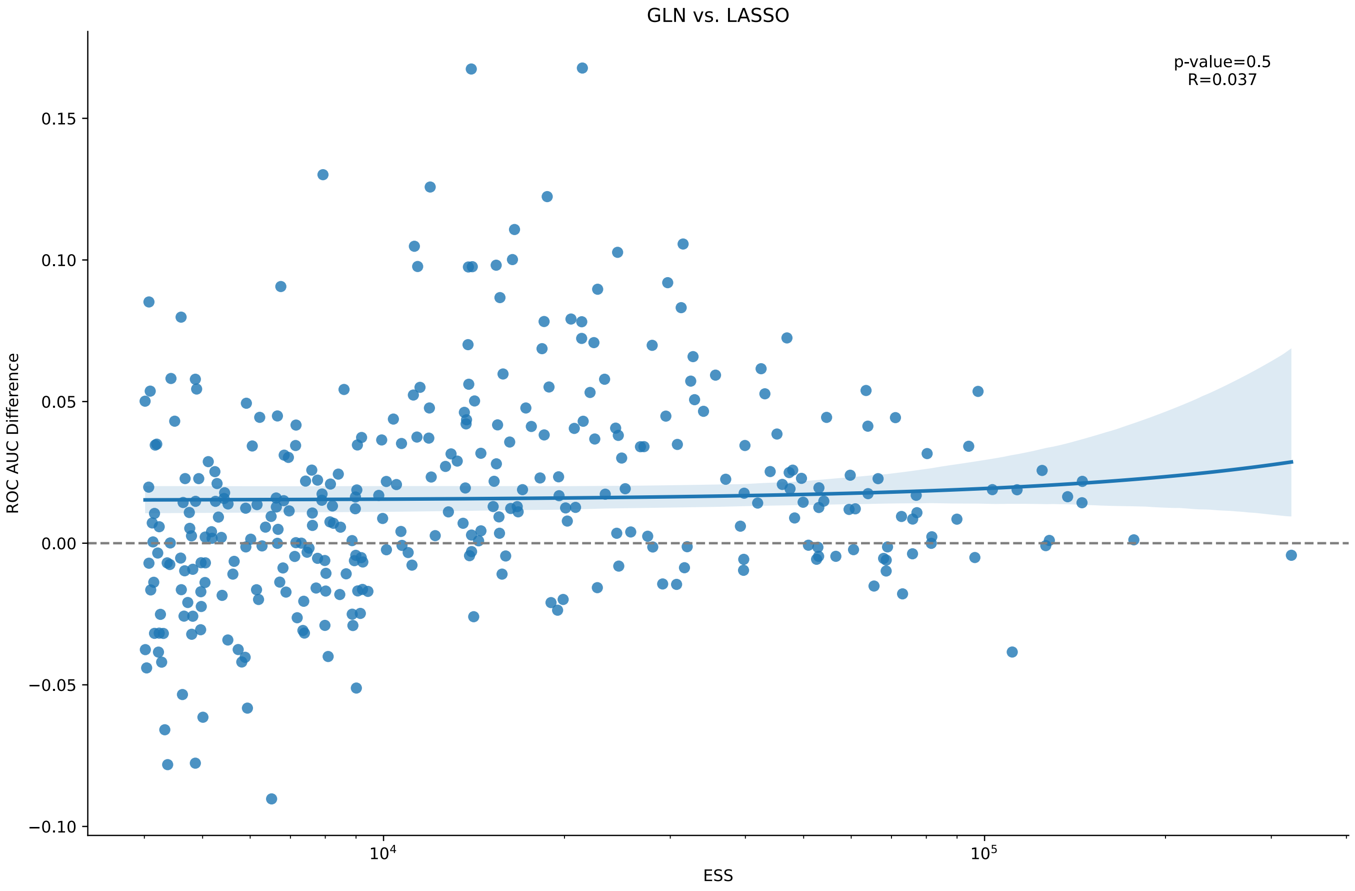

Supplementary Figure 40. Difference in performance of GLN model versus the LASSO model as a function of effective sample size (ESS). A positive difference indicates that the GLN model performed better on the test set compared to the LASSO.

Simulated Data Type	Model	Validation R^2	Parameters
Additive	Linear	0.9999	4001
Additive	CNN	0.9977	20877
Additive	MLP	0.9988	65732
Additive	GLN	0.9978	12810
Mix	Linear	0.7517	4001
Mix	CNN	0.9779	20877
Mix	MLP	0.9787	65732
Mix	GLN	0.9792	12810
XOR	Linear	-0.0291	4001
XOR	CNN	0.9611	20877
XOR	MLP	0.9479	65732
XOR	GLN	0.958	12810

Supplementary Table 1. Model comparison using simulated data. Mix refers to mixed effects of additive and XOR simulated SNP interactions. 12000 samples with 1,000 SNPs each were simulated.

L1	LR	NA	Val. ROC AUC	Test ROC AUC	Test 2.5%	Test 97.5%
1e-04	5e-06	0.0	0.6638			
1e-04	5e-05	0.0	0.6617			
1e-04	5e-04	0.0	0.6604			
1e-04	5e-03	0.0	0.5058			
1e-03	5e-06	0.0	0.6707			
1e-03	5e-05	0.0	0.6685			
1e-03	5e-04	0.0	0.6404			
1e-03	5e-03	0.0	0.5057			
1e-02	5e-06	0.0	0.6511			
1e-02	5e-05	0.0	0.6535			
1e-02	5e-04	0.0	0.6319			
1e-02	5e-03	0.0	0.5974			
1e-01	5e-06	0.0	0.5907			
1e-01	5e-05	0.0	0.5811			
1e-01	5e-04	0.0	0.5544			
1e-01	5e-03	0.0	0.534			
1e-03	5e-05	0.1	0.6742			
1e-03	5e-05	0.2	0.6736			
1e-03	5e-05	0.4	0.6765	0.6593	0.6301	0.6879

Supplementary Table 2. Comparison of various LASSO hyperparameter combinations tested when modelling on T1D. To reduce the chance of overfitting on the test set, only the model with the best performance on the validation set was considered, and its performance measured on the test set. CIs represent the 95% CI from 1,000 bootstrap replicates on the held-out test set.

rsID	Chr.	Gene	Top 3 LitVar	Top 3 DisGeNET
rs115469976	6			
rs3892710	6		Inflammation, Zellweger Syndrome, Coronary Artery Disease	
rs2281390	6			
rs58495148	6	HLA-DRB6		Child Development Disorders, Pervasive, Lymphoma, Follicular, Multiple Sclerosis
rs73405471	6	HLA-DQA1		Esophageal Achalasia, Celiac Disease, Diabetes Mellitus, Insulin-Dependent
rs984778	6			
rs146733600	6			
Affx-7384398	1			
rs7583093	2			
rs16954314	15	LOC105370766		
Affx-37072118	6			
rs79213347	15	SNAP23		Liver Cirrhosis, Experimental, Myocardial Ischemia, Diabetes Mellitus, Non-Insulin-Dependent
rs72811854	16			
rs2298483	11	CHEK1		Malignant Neoplasm Of Breast, Breast Carcinoma, Mammary Neoplasms
rs196023	6	CASC15		Neuroblastoma, Astigmatism, Regular Astigmatism - Corneal
rs4693440	4			
rs9270656	6			
rs9273364	6	HLA-DQB1-AS1		Chronic Obstructive Airway Disease
rs35915063	6	HLA-DQB1		Narcolepsy, Narcolepsy-Cataplexy Syndrome, Esophageal Achalasia
rs9273363	6	HLA-DQB1-AS1	Diabetes Mellitus, Arthritis, Rheumatoid, Autoimmune Diseases	Chronic Obstructive Airway Disease

Supplementary Table 3. Cross-reference of the 20 most highly important SNPs and known variant and gene associations for the GLN model trained on type 1 diabetes. The top 3 LitVar values were selected according to the most co-occuring disease entities. For DisGeNET, the top three scoring gene-disease DisGeNET association scores were used.

rsID	Chr.	Gene	Top 3 LitVar	Top 3 DisGeNET
rs3129871	6	HLA-DRA	Parkinson Disease, Multiple Sclerosis, Supranuclear Palsy, Progressive	Parkinson Disease, Multiple Sclerosis, Alcoholic Intoxication, Chronic
rs3104415	6			
rs3129716	6		Retinoschisis	
rs9273364	6	HLA-DQB1-AS1		Chronic Obstructive Airway Disease
rs115469976	6			
rs3892710	6		Inflammation, Zellweger Syndrome, Coronary Artery Disease	
rs9275334	6			
rs73405471	6	HLA-DQA1		Esophageal Achalasia, Celiac Disease, Diabetes Mellitus, Insulin-Dependent
rs28371206	6			
rs3135335	6			
rs3135006	6		Brain Stem Infarctions, N syndrome	
rs3957146	6		Diabetes Mellitus	
rs3842752	11	INS	Necrosis, Xeroderma Pigmentosum, Breast Neoplasms	Diabetes Mellitus, Permanent Neonatal, Maturity Onset Diabetes Mellitus In Young, Diabetes Mellitus
rs17555038	5	SGCD		Limb-Girdle Muscular Dystrophy Type 2F, Cardiomyopathy, Dilated, 1L, Cardiomyopathies
rs9273088	6	HLA-DQA1		Esophageal Achalasia, Celiac Disease, Diabetes Mellitus, Insulin-Dependent
rs9273363	6	HLA-DQB1-AS1	Diabetes Mellitus, Arthritis, Rheumatoid, Autoimmune Diseases	Chronic Obstructive Airway Disease
rs9275373	6		Parkinson Disease, Isobutyryl-CoA dehydrogenase deficiency	
rs3135395	6			
rs3104413	6		Autoimmune Diseases	
rs9275356	6			

Supplementary Table 4. Cross-reference of the 20 most highly important SNPs, on average across multiple training runs, and known variant and gene associations for the GLN models trained on type 1 diabetes. The top SNPs are chosen according to the average of highest imporant SNPs across 10 runs with different random seeds each. The top 3 LitVar values were selected according to the most co-occuring disease entities. For DisGeNET, the top three scoring gene-disease DisGeNET association scores were used.

**Allele**	β	**Std. Err.**	**Z**	$P > \|z\|$	0.025 **CI**	0.975 **CI**	**Odds**
rs9273364 TT	-5.274	0.033	-158.4	0	-5.339	-5.208	0.005125
rs9273364 TG	-4.878	0.029	-166.1	0	-4.935	-4.82	0.007615
rs9273364 GG	-3.808	0.038	-100	0	-3.882	-3.733	0.0222
rs9275601 CC	-5.033	0.037	-137.6	0	-5.105	-4.961	0.00652
rs9275601 CT	-4.888	0.027	-181.3	0	-4.941	-4.835	0.007539
rs9275601 TT	-4.609	0.038	-122.8	0	-4.682	-4.535	0.009964
rs4506565 AA	-4.993	0.029	-172	0	-5.049	-4.936	0.006788
rs4506565 AT	-4.794	0.028	-173.3	0	-4.848	-4.74	0.008279
rs4506565 TT	-4.662	0.054	-85.81	0	-4.769	-4.556	0.009447
rs9268880 GG	-4.672	0.025	-186.9	0	-4.721	-4.623	0.009352
rs9268880 GT	-5.188	0.037	-140.6	0	-5.26	-5.116	0.005583
rs9268880 TT	-5.408	0.093	-57.87	0	-5.591	-5.225	0.00448
rs7903146 CC	-4.987	0.028	-176.3	0	-5.042	-4.931	0.006829
rs7903146 CT	-4.792	0.028	-168.7	0	-4.848	-4.736	0.008295
rs7903146 TT	-4.626	0.058	-79.47	0	-4.74	-4.512	0.009795
rs3129871 CC	-4.492	0.024	-184.4	0	-4.54	-4.444	0.0112
rs3129871 CA	-5.22	0.033	-158.2	0	-5.285	-5.155	0.005407
rs3129871 AA	-5.363	0.067	-79.55	0	-5.496	-5.231	0.004685
rs72830085 GG	-4.893	0.024	-208.1	0	-4.939	-4.847	0.007498
rs72830085 GA	-4.732	0.06	-79.39	0	-4.849	-4.615	0.008811
rs72830085 AA	-5.017	0.379	-13.23	5.831×10^{-40}	-5.761	-4.274	0.006622
rs11749675 CC	-4.834	0.021	-233.3	0	-4.875	-4.794	0.007952
rs11749675 CT	-5.009	0.046	-108.2	0	-5.1	-4.918	0.006677
rs11749675 TT	-5.133	0.201	-25.59	1.937×10^{-144}	-5.526	-4.74	0.005898
rs62344507 GG	-4.894	0.02	-239.3	0	-4.935	-4.854	0.007488
rs62344507 GA	-4.671	0.05	-93.33	0	-4.769	-4.573	0.009364
rs62344507 AA	-4.91	0.317	-15.47	5.421×10^{-54}	-5.533	-4.288	0.00737
rs1719971 CC	-4.9	0.024	-205.5	0	-4.947	-4.854	0.007444
rs1719971 CT	-4.804	0.032	-149	0	-4.867	-4.741	0.008199
rs1719971 TT	-4.892	0.097	-50.41	0	-5.082	-4.702	0.007507
rs9273363 CC	-5.278	0.033	-160.2	0	-5.343	-5.214	0.005102
rs9273363 CA	-4.876	0.029	-167.9	0	-4.933	-4.819	0.007631
rs9273363 AA	-3.81	0.037	-101.9	0	-3.883	-3.736	0.02216
rs13167249 GG	-4.857	0.034	-140.8	0	-4.925	-4.79	0.007773
rs13167249 GT	-4.935	0.028	-179.1	0	-4.989	-4.881	0.007192
rs13167249 TT	-4.734	0.039	-122.6	0	-4.81	-4.658	0.008792
rs73735325 TT	-4.901	0.021	-235.1	0	-4.942	-4.86	0.007441
rs73735325 TC	-4.721	0.045	-105.6	0	-4.808	-4.633	0.008909
rs73735325 CC	-4.624	0.197	-23.46	9.925×10^{-122}	-5.01	-4.238	0.009815
rs2102484 CC	-4.8	0.035	-138.1	0	-4.868	-4.732	0.008228
rs2102484 CT	-4.954	0.028	-178.4	0	-5.009	-4.9	0.007054
rs2102484 TT	-4.777	0.038	-126	0	-4.851	-4.702	0.008423
Affx-4739950 GG	-4.748	0.029	-164.3	0	-4.805	-4.692	0.008665
Affx-4739950 GA	-4.941	0.028	-174.9	0	-4.997	-4.886	0.007145
Affx-4739950 AA	-4.968	0.051	-97.13	0	-5.068	-4.867	0.006961
rs6679677 CC	-4.948	0.022	-227.5	0	-4.991	-4.905	0.007098
rs6679677 CA	-4.601	0.039	-119	0	-4.677	-4.526	0.01004
rs6679677 AA	-4.296	0.14	-30.77	7.044×10^{-208}	-4.569	-4.022	0.01363
rs3842752 GG	-4.735	0.023	-208.1	0	-4.78	-4.691	0.008781
rs3842752 GA	-5.111	0.036	-142.8	0	-5.181	-5.04	0.006033
rs3842752 AA	-5.083	0.092	-55.04	0	-5.264	-4.902	0.006203
rs610101 CC	-4.955	0.031	-158.2	0	-5.016	-4.893	0.007049
rs610101 CT	-4.822	0.027	-179.6	0	-4.875	-4.769	0.008051
rs610101 TT	-4.791	0.049	-98.59	0	-4.886	-4.696	0.008304
rs3842753 GG	-4.695	0.026	-182.6	0	-4.745	-4.644	0.009143
rs3842753 GT	-5.071	0.034	-148.3	0	-5.138	-5.004	0.006279
rs3842753 TT	-5.138	0.083	-61.91	0	-5.301	-4.976	0.005867
rs9271348 AA	-4.58	0.025	-184.3	0	-4.628	-4.531	0.01026
rs9271348 AG	-5.316	0.039	-137.3	0	-5.392	-5.24	0.004911
rs9271348 GG	-5.364	0.082	-65.1	0	-5.525	-5.202	0.004683

Supplementary Table 5. Results from fitting a logistic regression model on the top 20 most highly important SNPs identified by the DL GLN model for type 1 diabetes prediction. Ten training runs with different seeds were performed with the GLN model, and the top 20 SNPs across all runs (measured by absolute average SHAP effect on the validation set) used as candidates for the analysis. For each SNP, a logistic regression model was fit with the different genotypes encoded in a one-hot format. The fitted coefficients show indication of both additive and non-additive effects.

rsID	Chr.	Gene	Top 3 LitVar	Top 3 DisGeNET
rs3731239	9	CDKN2A	Breast Neoplasms, Neoplasms, N syndrome	Esophageal Neoplasms, Lung Neoplasms, Melanoma
rs17017475	1			
rs2427221	20	CDH4		Longevity, Sarcoidosis, Polysomnography
rs12405608	1			
rs34410881	1			
rs2858584	22			
rs7355020	1	NAV1		Schizophrenia, Body Height, Cardiovascular Diseases
rs1043811	12	RBM19		Diabetes Mellitus, Non-Insulin-Dependent, Response To Simvastatin, Blepharoptosis
rs10871782	18			
rs75602167	22	TOP3B	Alzheimer Disease	Schizophrenia, Autistic Disorder, Impaired Cognition
rs4977574	9	CDKN2B-AS1	Coronary Disease, Coronary Artery Disease, Myocardial Infarction	Endometriosis, Glaucoma, Open-Angle, Nasopharyngeal Carcinoma
rs2549505	16	MAF		Cataract 21, Multiple Types, Cataracts, Congenital, With Sensorineural Deafness, Down Syndrome-Like Facial Appearance, Short Stature, And Mental Retardation, Cataract, Pulverulent, Juvenile-Onset
rs17705635	18	CHST9		Malignant Neoplasm Of Breast, Adolescent Idiopathic Scoliosis, Breast Carcinoma
rs6128184	20			
rs8005039	14	AKAP6		Atrial Fibrillation, Intellectual Disability, Malignant Neoplasm Of Breast
rs72699511	1			
rs10784085	12			
rs149144163	20			
rs3184504	12	SH2B3	Diabetes Mellitus, Arthritis, Rheumatoid, Celiac Disease	Precursor Cell Lymphoblastic Leukemia Lymphoma, Thrombocythemia, Essential, Diabetes Mellitus, Insulin-Dependent
rs71331632	21			

Supplementary Table 6. Cross-reference of the 20 most highly important SNPs and known variant and gene associations for the GLN model trained on acute myocardial infarction. The top 3 LitVar values were selected according to the most co-occuring disease entities. For DisGeNET, the top three scoring gene-disease DisGeNET association scores were used.

rsID	Chr.	Gene	Top 3 LitVar	Top 3 DisGeNET
rs10975479	9			
rs340921	9			
rs8056488	16			
rs71430382	2	D2HGDH		D-2-Hydroxyglutaric Aciduria 1, Combined D-2- And L-2-Hydroxyglutaric Aciduria, Amino Acid Metabolism, Inborn Errors
rs17293632	15	SMAD3	Crohn Disease, Inflammatory Bowel Diseases, Arthritis, Rheumatoid	Loeys-Dietz Syndrome 3, Colorectal Carcinoma, Loeys-Dietz Syndrome
rs2160203	2	IL1RL1	Diabetes Mellitus, Asthma, Rhinitis	Asthma, Arthritis, Adjuvant-Induced, Acute Lung Injury
rs343476	9			
rs72777284	2			
rs111543205	2	D2HGDH		D-2-Hydroxyglutaric Aciduria 1, Combined D-2- And L-2-Hydroxyglutaric Aciduria, Amino Acid Metabolism, Inborn Errors
rs2589559	10			
rs167769	12	STAT6	Asthma, Alzheimer Disease, Pain	Solitary Fibrous Tumor, Asthma, Dermatitis, Atopic
Affx-37000939	5			
rs9050	1	TCHH	Alzheimer Disease, Properdin deficiency, X-linked	Uncombable Hair Syndrome, Dermatitis, Atopic, Curly Hair (Finding)
rs11642659	16	LCMT1		Blastocyst Disintegration, Embryo Resorption, Embryo Death
rs2706347	5	RAD50	Asthma, Dermatitis, Atopic, Rhinitis	Nijmegen Breakage Syndrome-Like Disorder, Asthma, Breast Cancer, Familial
rs870301	2	BOK-AS1		Forced Expiratory Volume Function, Inflammatory Bowel Diseases, Vital Capacity
rs17612633	6	HLA-DQA1		Esophageal Achalasia, Celiac Disease, Diabetes Mellitus, Insulin-Dependent
Affx-8225028	12			
rs62298921	3			
rs3771180	2	IL1RL1	Asthma, Rhinitis, Allergic, Seasonal, Obesity	Asthma, Arthritis, Adjuvant-Induced, Acute Lung Injury

Supplementary Table 7. Cross-reference of the 20 most highly important SNPs and known variant and gene associations for the GLN model trained on asthma. The top 3 LitVar values were selected according to the most co-occuring disease entities. For DisGeNET, the top three scoring gene-disease DisGeNET association scores were used.

rsID	Chr.	Gene	Top 3 LitVar	Top 3 DisGeNET
rs17042171	4		Atrial Fibrillation, Diabetes Mellitus, Hypertension	
rs3853445	4		Atrial Fibrillation, Hypertension, Properdin deficiency, X-linked	
rs17042081	4			
rs13141190	4			
rs10033464	4		Atrial Fibrillation, Stroke, Cerebral Infarction	
rs1448817	4		Atrial Fibrillation	
rs4074536	1	CASQ2	Tachycardia, Ventricular, Long QT Syndrome, Atrial Fibrillation	Stress-Induced Polymorphic Ventricular Tachycardia, Ventricular Tachycardia, Catecholaminergic Polymorphic, 1 (Disorder), Tachycardia, Ventricular
rs1906610	4			
rs13376333	1	KCNN3	Atrial Fibrillation, Hypertension, Diabetes Mellitus	Zimmerman Laband Syndrome, Atrial Fibrillation, Schizophrenia
rs7667461	4			
rs521511	4			
rs72811957	17	NTN1		Mirror Movements 4, Subarachnoid Hemorrhage, Cerebral Edema
rs117984853	6		Atrial Fibrillation	
rs10908444	1	KCNN3		Zimmerman Laband Syndrome, Atrial Fibrillation, Schizophrenia
rs1386389	4			
rs17825726	22			
rs5765546	22			
rs4845695	1	PMVK	Drug-Related Side Effects and Adverse Reactions, 13q deletion syndrome	Porokeratosis Of Mibelli, Porokeratosis, Linear, Porokeratosis
rs2106261	16	ZFHX3	Atrial Fibrillation, Hypertension, Inflammation	Atrial Fibrillation, Cerebrovascular Accident, Malignant Neoplasm Of Prostate
rs5886821	7	LOC107986838		

Supplementary Table 8. Cross-reference of the 20 most highly important SNPs and known variant and gene associations for the GLN model trained on atrial fibrillation and flutter. The top 3 LitVar values were selected according to the most co-occuring disease entities. For DisGeNET, the top three scoring gene-disease DisGeNET association scores were used.

rsID	Chr.	Gene	Top 3 LitVar	Top 3 DisGeNET
rs117888135	10			
rs12940887	17	ZNF652	Arthritis, Rheumatoid, Sveinsson Chorioretinal Atrophy, Ataxia Telangiectasia	Malignant Neoplasms, Malignant Neoplasm Of Prostate, Prostate Carcinoma
rs34328549	19	INSR		Donohue Syndrome, Rabson-Mendenhall Syndrome, Diabetes Mellitus, Non-Insulin-Dependent
rs4932370	15		Schmid-Fraccaro syndrome, Ataxia Telangiectasia	
rs9647448	4	LCORL		Birth Weight, Body Height, Cardiovascular Diseases
rs4987082	17	PHB	Autistic Disorder	Malignant Neoplasm Of Breast, Mammary Neoplasms, Experimental, Breast Carcinoma
rs160889	5			
rs16834635	1	PBX1		Burkitt Lymphoma, Congenital Anomalies Of Kidney And Urinary Tract Syndrome With Or Without Hearing Loss, Abnormal Ears, Or Developmental Delay, Precursor B-Cell Lymphoblastic Leukemia
rs4766897	12	ACAD10		Age Related Macular Degeneration, Alcohol Consumption, Coronary Heart Disease
rs6848130	4			
rs52824916	12	FAM186B		Nephronophthisis, Systolic Pressure
rs2963446	5			
rs356986	2			
rs35208507	16	PDILT		Colorectal Carcinoma, Blood Urea Nitrogen Measurement, Cardiovascular Diseases
rs35112858	8	MSRA		Schizophrenia, Drug Abuse, Drug Habituation
rs7354757	21	MORC3		Body Height, White Blood Cell Count Procedure, Diastolic Blood Pressure
rs10004996	4	LINC02513		Lymphocyte Count Measurement
rs7561317	2		Obesity, Diabetes Mellitus, Type 2, Diabetes Mellitus	
rs13115333	4			
rs1054707	4	BDH2		Neoplasms, Iron Deficiency, Tumor Cell Invasion

Supplementary Table 9. Cross-reference of the 20 most highly important SNPs and known variant and gene associations for the GLN model trained on hypertension. The top 3 LitVar values were selected according to the most co-occuring disease entities. For DisGeNET, the top three scoring gene-disease DisGeNET association scores were used.

rsID	Chr.	Gene	Top 3 LitVar	Top 3 DisGeNET
rs733175	4		Alzheimer Disease, Parkinson Disease, Nephrolithiasis	
rs2231142	4	ABCG2	Gout, Hyperuricemia, Neoplasms	Uric Acid Concentration, Serum, Quantitative Trait Locus 1, Hyperuricemia, Gout
rs4148157	4	ABCG2	Brain Neoplasms, Properdin deficiency, X-linked	Uric Acid Concentration, Serum, Quantitative Trait Locus 1, Hyperuricemia, Gout
rs7671266	4		Ataxia Telangiectasia	
rs6855911	4	SLC2A9	Anxiety Disorders, Phobic Disorders, Metabolic Diseases	Hypouricemia, Renal, 2, Renal Hypouricemia, Hyperuricemia
rs13129697	4	SLC2A9	Diabetes Mellitus, Kidney Diseases, Hypertension	Hypouricemia, Renal, 2, Renal Hypouricemia, Hyperuricemia
rs737267	4	SLC2A9	Glycogen Storage Disease Type V, Parkinson Disease, Hypertension	Hypouricemia, Renal, 2, Renal Hypouricemia, Hyperuricemia
rs4698023	4			
rs4481233	4	SLC2A9	Familial benign hypercalcemia, type 3, Gout	Hypouricemia, Renal, 2, Renal Hypouricemia, Hyperuricemia
rs73203233	21			
rs16890979	4	SLC2A9	Gout, Zellweger Syndrome, Nephrolithiasis	Hypouricemia, Renal, 2, Renal Hypouricemia, Hyperuricemia
rs7671500	4			
rs11048289	12			
rs6834555	4		Alzheimer Disease, Psychoses, Substance-Induced, Properdin deficiency, X-linked	
rs7442295	4	SLC2A9	Glycogen Storage Disease Type V, Myocardial Ischemia, Gout	Hypouricemia, Renal, 2, Renal Hypouricemia, Hyperuricemia
rs995014	21	N6AMT1		Glomerular Filtration Rate, Malignant Neoplasms, Primary Malignant Neoplasm
rs4809565	20	KCNQ2		Seizures, Benign Familial Neonatal, 1, Epileptic Encephalopathy, Early Infantile, 7, Familial Benign Neonatal Epilepsy
rs4910001	11	GALNT18		Rheumatoid Arthritis, Serum Albumin Measurement, Gastric Adenocarcinoma
rs3114020	4	ABCG2	Carcinoma, Non-Small-Cell Lung, Properdin deficiency, X-linked, Adenocarcinoma	Uric Acid Concentration, Serum, Quantitative Trait Locus 1, Hyperuricemia, Gout
rs3760627	19	CLPTM1	Alzheimer Disease	Pancreatic Neoplasm, Malignant Neoplasm Of Pancreas, Alzheimer'S Disease

Supplementary Table 10. Cross-reference of the 20 most highly important SNPs and known variant and gene associations for the GLN model trained on gout. The top 3 LitVar values were selected according to the most co-occuring disease entities. For DisGeNET, the top three scoring gene-disease DisGeNET association scores were used.

rsID	Chr.	Gene	Top 3 LitVar	Top 3 DisGeNET
rs4506565	10	TCF7L2	Diabetes Mellitus, Diabetes Mellitus, Type 2, Obesity	Colorectal Carcinoma, Diabetes Mellitus, Non-Insulin-Dependent, Colorectal Neoplasms
rs12243326	10	TCF7L2	Polycystic Ovary Syndrome, Diabetes Mellitus, Hypoglycemia	Colorectal Carcinoma, Diabetes Mellitus, Non-Insulin-Dependent, Colorectal Neoplasms
rs12255372	10	TCF7L2	Diabetes Mellitus, Diabetes Mellitus, Type 2, Obesity	Colorectal Carcinoma, Diabetes Mellitus, Non-Insulin-Dependent, Colorectal Neoplasms
rs11196175	10	TCF7L2	Metabolic Diseases, Choroideremia, Ovarian Neoplasms	Colorectal Carcinoma, Diabetes Mellitus, Non-Insulin-Dependent, Colorectal Neoplasms
rs7903146	10	TCF7L2	Diabetes Mellitus, Diabetes Mellitus, Type 2, Obesity	Colorectal Carcinoma, Diabetes Mellitus, Non-Insulin-Dependent, Colorectal Neoplasms
rs2796441	9	LOC101927502	Diabetes Mellitus, Diabetes Mellitus, Type 2, Ataxia Telangiectasia	
rs62208714	20	PTPRT		Leukemia, Myelocytic, Acute, Colorectal Carcinoma, Malignant Neoplasm Of Lung
rs11257655	10		Properdin deficiency, X-linked, Diabetes Mellitus, Diabetes Mellitus, Type 2	
rs7069060	10		Retinoschisis	
rs7018475	9		Diabetes Mellitus, Obesity, Histidinemia	
rs9273363	6	HLA-DQB1-AS1	Diabetes Mellitus, Arthritis, Rheumatoid, Autoimmune Diseases	Chronic Obstructive Airway Disease
rs163177	11	KCNQ1	Properdin deficiency, X-linked, Coronary Artery Disease	Jervell-Lange Nielsen Syndrome, Long Qt Syndrome 1, Short Qt Syndrome 2 (Disorder)
rs17817449	16	FTO	Obesity, Breast Neoplasms, Diabetes Mellitus	Growth Retardation, Developmental Delay, Coarse Facies, And Early Death, Malignant Neoplasm Of Breast, Diabetes Mellitus, Non-Insulin-Dependent
rs388508	10	LYZL2		Dental Caries
rs35368011	5	C5orf67		Alcohol Consumption, Cardiovascular Diseases, Diabetes Mellitus, Non-Insulin-Dependent
rs12221133	10	CDC123		Diabetes Mellitus, Non-Insulin-Dependent, Fibrosarcoma, Forced Expiratory Volume Function
rs2484892	9			
rs62020600	16	TMEM114		Cholelithiasis, Vital Capacity, Cholecystolithiasis
rs1046320	4	WFS1	Diabetes Mellitus, Type 2, Diabetes Mellitus	Wolfram Syndrome 1, Wolfram Syndrome, Wolfram-Like Syndrome, Autosomal Dominant
rs1572053	20	EYA2		Alcohol Consumption, Diabetes Mellitus, Non-Insulin-Dependent, Eczema

Supplementary Table 11. Cross-reference of the 20 most highly important SNPs and known variant and gene associations for the GLN model trained on type 2 diabetes. The top 3 LitVar values were selected according to the most co-occuring disease entities. For DisGeNET, the top three scoring gene-disease DisGeNET association scores were used.

rsID	Chr.	Gene	Top 3 LitVar	Top 3 DisGeNET
rs231727	2		Polyendocrinopathies, Autoimmune, 211750, Ataxia Telangiectasia	
rs3184504	12	SH2B3	Diabetes Mellitus, Arthritis, Rheumatoid, Celiac Disease	Precursor Cell Lymphoblastic Leukemia Lymphoma, Thrombocythemia, Essential, Diabetes Mellitus, Insulin-Dependent
rs17364832	13	SPATA13		Autoimmune Diseases, Hypothyroidism, Respiratory Function Tests
rs653178	12	ATXN2	Arthritis, Rheumatoid, Diabetes Mellitus, Celiac Disease	Spinocerebellar Ataxia Type 2, Amyotrophic Lateral Sclerosis, Parkinson Disease, Late-Onset
rs10028213	4	LOC107986195	Hypothyroidism, Thyroid Neoplasms	
rs1348386	9	PTCSC2		Thyroid Carcinoma, Alopecia Areata, Autoimmune Diseases
rs7323885	13	SPATA13		Autoimmune Diseases, Hypothyroidism, Respiratory Function Tests
rs2412970	22	HORMAD2	Colitis, Crohn Disease, Isobutyryl-CoA dehydrogenase deficiency	Malignant Neoplasm Of Lung, Iga Glomerulonephritis, Ulcerative Colitis
rs925489	9	PTCSC2	Thyroid cancer, papillary, Autoimmune Diseases, Hypertension	Thyroid Carcinoma, Alopecia Areata, Autoimmune Diseases
rs310405	6		Meige Syndrome, Charcot-Marie-Tooth disease, Type 1D	
rs2412971	22	HORMAD2	Inflammatory Bowel Diseases, Isobutyryl-CoA dehydrogenase deficiency, Proteinuria	Malignant Neoplasm Of Lung, Iga Glomerulonephritis, Ulcerative Colitis
rs6679677	1	PHTF1	Arthritis, Rheumatoid, Diabetes Mellitus, Lupus Erythematosus, Systemic	Rheumatoid Arthritis, Diabetes Mellitus, Insulin-Dependent
rs7574865	2	STAT4	Lupus Erythematosus, Systemic, Arthritis, Rheumatoid, Autoimmune Diseases	Lupus Erythematosus, Systemic, Behcet Syndrome, Rheumatoid Arthritis
rs12575636	11		Gaucher Disease	
rs654537	6	BACH2		Crohn Disease, Diabetes Mellitus, Insulin-Dependent, Celiac Disease
rs1024161	2		Graves Disease, Diabetes Mellitus, Diabetes Mellitus, Type 1	
rs10452226	4	LOC105377483		
rs118173218	14	ESRRB		Deafness, Autosomal Recessive 35, Hearing Impairment, Nonsyndromic Deafness
rs2233955	6	C6orf15		Sarcoidosis, Human Immunodeficiency Virus Type 1, Susceptibility To, Hiv-1, Resistance To
rs4818324	21			

Supplementary Table 12. Cross-reference of the 20 most highly important SNPs and known variant and gene associations for the GLN model trained on hypothyroidism. The top 3 LitVar values were selected according to the most co-occuring disease entities. For DisGeNET, the top three scoring gene-disease DisGeNET association scores were used.

rsID	Chr.	Gene	Top 3 LitVar	Top 3 DisGeNET
rs532965	6			
rs6931277	6		Alzheimer Disease	
rs3104413	6		Autoimmune Diseases	
rs3830127	6	HLA-DRB1		Crohn Disease, Lupus Erythematosus, Systemic, Narcolepsy
rs34250758	6	HLA-DQA1		Esophageal Achalasia, Celiac Disease, Diabetes Mellitus, Insulin-Dependent
rs2760976	6			
rs34855541	6			
rs521539	6			
rs2858333	6		Testicular Neoplasms	
rs660895	6		Parkinson Disease, Arthritis, Rheumatoid, Lupus Erythematosus, Systemic	
rs2647087	6		Pancreatitis, Colonic Diseases, Testicular Neoplasms	
rs7745656	6		Pancreatitis	
rs35265698	6			
rs9268515	6		Parkinson Disease, Properdin deficiency, X-linked	
rs9275555	6			
rs5004277	6			
rs3793127	6	BTNL2	211750, Ataxia Telangiectasia	Sarcoidosis, Berylliosis, Beryllium Disease
Affx-28502467	6			
rs3104415	6			
rs111586361	6	HLA-DRB6		Child Development Disorders, Pervasive, Lymphoma, Follicular, Multiple Sclerosis

Supplementary Table 13. Cross-reference of the 20 most highly important SNPs and known variant and gene associations for the LASSO model trained on rheumatoid arthritis. The top 3 LitVar values were selected according to the most co-occuring disease entities. For DisGeNET, the top three scoring gene-disease DisGeNET association scores were used.

rsID	Chr.	Gene	Top 3 LitVar	Top 3 DisGeNET
rs474235	4	NSD2		Wolf-Hirschhorn Syndrome, Pitt-Rogers-Danks Syndrome, Microcephaly
rs55994383	15			
rs2395163	6		Parkinson Disease, Schizophrenia, Parkinson Disease, Secondary	
rs4309342	15			
rs7600206	2	PAX3		Alveolar Rhabdomyosarcoma, Waardenburg Syndrome Type 1, Waardenburg Syndrome
rs115079157	2			
rs3830127	6	HLA-DRB1		Crohn Disease, Lupus Erythematosus, Systemic, Narcolepsy
rs117290280	16	RAB11FIP3		Platelet Count Measurement, Mean Corpuscular Volume (Result), Finding Of Mean Corpuscular Hemoglobin
rs1273150	14	DAAM1		Body Height, Tumor Cell Invasion, Malignant Neoplasm Of Breast
rs75021444	8			
rs9928176	16			
rs660895	6		Parkinson Disease, Arthritis, Rheumatoid, Lupus Erythematosus, Systemic	
rs9272226	6		Lupus Erythematosus, Systemic	
rs262929	6			
rs2179685	20			
rs62033174	16			
rs9268645	6	HLA-DRA	Diabetes Mellitus, Lupus Erythematosus, Systemic, Arthritis, Rheumatoid	Parkinson Disease, Multiple Sclerosis, Alcoholic Intoxication, Chronic
rs73248761	13			
rs1320591	1			
rs12044963	1	KCND3	Atrial Fibrillation, Brain Stem Infarctions	Spinocerebellar Ataxia 19, Brugada Syndrome 9, Brugada Syndrome (Disorder)

Supplementary Table 14. Cross-reference of the 20 most highly important SNPs and known variant and gene associations for the GLN model trained on rheumatoid arthritis. The top 3 LitVar values were selected according to the most co-occuring disease entities. For DisGeNET, the top three scoring gene-disease DisGeNET association scores were used.

rsID	Chr.	Gene	Top 3 LitVar	Top 3 DisGeNET
rs12255372	10	TCF7L2	Diabetes Mellitus, Diabetes Mellitus, Type 2, Obesity	Colorectal Carcinoma, Diabetes Mellitus, Non-Insulin-Dependent, Colorectal Neoplasms
rs7903146	10	TCF7L2	Diabetes Mellitus, Diabetes Mellitus, Type 2, Obesity	Colorectal Carcinoma, Diabetes Mellitus, Non-Insulin-Dependent, Colorectal Neoplasms
rs76769781	20			
rs117697300	21			
rs12243326	10	TCF7L2	Polycystic Ovary Syndrome, Diabetes Mellitus, Hypoglycemia	Colorectal Carcinoma, Diabetes Mellitus, Non-Insulin-Dependent, Colorectal Neoplasms
rs1880008	22			
rs4506565	10	TCF7L2	Diabetes Mellitus, Diabetes Mellitus, Type 2, Obesity	Colorectal Carcinoma, Diabetes Mellitus, Non-Insulin-Dependent, Colorectal Neoplasms
rs4751323	10	DOCK1		Drug Abuse, Drug Habituation, Drug Use Disorders
rs7752155	6			
rs6882745	5			
rs251404	5	PIK3R1		Short Syndrome, Insulin Resistance, Agammaglobulinemia 7, Autosomal Recessive
rs13124487	4	PPP2R2C		Bipolar Disorder, Pathological Accumulation Of Air In Tissues, Diabetes Mellitus, Non-Insulin-Dependent
rs17065659	5			
rs4431094	3			
rs713928	22	TAFA5		Pancreatic Carcinoma, Malignant Neoplasm Of Pancreas, Adenovirus Infections
rs2869310	20			
rs1106440	7	CNTNAP2		Pitt-Hopkins-Like Syndrome 1, Cortical Dysplasia With Focal Epilepsy Syndrome, Autism Spectrum Disorders
rs3016382	11	OPCML		Ovarian Neoplasm, Malignant Neoplasm Of Ovary, Schizophrenia
rs11176379	12	GRIP1		Cryptophthalmos Syndrome, Fraser Syndrome 3, Schizophrenia
rs79768529	4	LOC105377567		

Supplementary Table 15. Cross-reference of the 20 most highly important SNPs and known variant and gene associations for the GLN model trained on type 2 diabetes, when including clinical and biochemical measurements. The top 3 LitVar values were selected according to the most co-occuring disease entities. For DisGeNET, the top three scoring gene-disease DisGeNET association scores were used.

DBDS Affiliations

Karina Banasik[1], Jakob Bay[2], Jens Kjærgaard Boldsen[3], Thorsten Brodersen[2], Søren Brunak[1], Kristoffer Burgdorf[1], Mona Ameri Chalmer[4], Maria Didriksen[5], Khoa Manh Dinh[3], Joseph Dowsett[5], Christian Erikstrup[3], Bjarke Feenstra[5], Frank Geller[5], Daniel Gudbjartsson[6], Thomas Folkmann Hansen[4], Lotte Hindhede[3], Henrik Hjalgrim[7], Rikke Louise Jacobsen[5], Gregor Jemec[8], Katrine Kaspersen[3], Bertram Dalskov Kjerulff[3], Lisette Kogelman [4], Margit Anita Hørup Larsen[5], Ioannis Louloudis [1], Agnete Lundgaard[1], Susan Mikkelsen[3], Christina Mikkelsen[5], Kaspar Rene Nielsen[9], Ioanna Nissen[5], Mette Nyegaard[10], Sisse Rye Ostrowski[5], Ole Birger Pedersen[2], Alexander Pil Henriksen[1], Palle Duun Rohde[10], Klaus Rostgaard[11], Michael Schwinn[5], Kari Stefansson[6], Hreinn Stefánsson[6], Erik Sørensen[5], Unnur Thorsteinsdóttir[6], Lise Wegner Thørner[5], Mie Topholm Bruun[12], Henrik Ullum[13], Thomas Werge[14], David Westergaard[1]

[1] Novo Nordisk Foundation Center for Protein Research, Faculty of Health and Medical Sciences, University of Copenhagen, Copenhagen, Denmark [2] Department of Clinical Immunology, Zealand University Hospital, Køge, Denmark [3] Department of Clinical Immunology, Aarhus University Hospital, Aarhus, Denmark [4] Danish Headache Center, Department of Neurology, Copenhagen University Hospital, Rigshospitalet-Glostrup, Copenhagen, Denmark [5] Department of Clinical Immunology, Copenhagen University Hospital, Rigshospitalet, Copenhagen, Denmark [6] deCODE Genetics, Reykjavik, Iceland [7] Danish Cancer Society Research Center, Copenhagen, Denmark [8] Department of Clinical Medicine, Zealand University hospital, Roskilde, Denmark [9] Department of Clinical Immunology, Aalborg University Hospital, Aalborg, Denmark [10] Department of Health Science and Technology, Faculty of Medicine, Aalborg Univeristy, Aalborg, Denmark [11] Danish Cancer Society, Copenhagen, Denmark [12] Department of Clinical Immunology, Odense University Hospital, Odense, Denmark [13] Statens Serum Institut, Copenhagen, Denmark [14] Institute of Biological Psychiatry, Mental Health Centre, Sct. Hans, Copenhagen University Hospital, Roskilde, Denmark
